# Long-read-based human genomic structural variation detection with cuteSV

**DOI:** 10.1186/s13059-020-02107-y

**Published:** 2020-08-03

**Authors:** Tao Jiang, Yongzhuang Liu, Yue Jiang, Junyi Li, Yan Gao, Zhe Cui, Yadong Liu, Bo Liu, Yadong Wang

**Affiliations:** 1 Center for Bioinformatics, School of Computer Science and Technology, Harbin Institute of Technology, Harbin, 150001 Heilongjiang China; 2 Nebula Genomics, Harbin, 150030 Heilongjiang China; 3 School of Computer Science and Technology, Harbin Institute of Technology (Shenzhen), Shenzhen, 518055 Guangdong China

**Keywords:** Structural variants detection, Long-read sequencing, Scaling performance

## Abstract

Long-read sequencing is promising for the comprehensive discovery of structural variations (SVs). However, it is still non-trivial to achieve high yields and performance simultaneously due to the complex SV signatures implied by noisy long reads. We propose cuteSV, a sensitive, fast, and scalable long-read-based SV detection approach. cuteSV uses tailored methods to collect the signatures of various types of SVs and employs a clustering-and-refinement method to implement sensitive SV detection. Benchmarks on simulated and real long-read sequencing datasets demonstrate that cuteSV has higher yields and scaling performance than state-of-the-art tools. cuteSV is available at https://github.com/tjiangHIT/cuteSV.

## Background

Structural variations (SVs) represent genomic rearrangements such as deletions, insertions, inversions, duplications, and translocations whose sizes are larger than 50 bp [1]. As the largest divergences across human genomes [2], SVs are closely related to human diseases (e.g., inherited diseases [3–5] and cancers [6]), evolution (e.g., gene losses and transposon activity [7, 8]), gene regulations (e.g., rearrangements of transcription factors [9]), and other phenotypes (e.g., mating and intrinsic reproductive isolation [10, 11]).

Efforts have been made to develop short-read-based SV calling approaches [12, 13]. Most of them use the methods such as read-depths [14], discordant read-pairs [15], split read alignments [16], local assembly [17], or their combinations [18–20], and they have played important roles in large-scale genomics studies such as 1000 Genomes Project [1]. However, the relatively low read length limits these tools to implement sensitive SV detection [21], and false positives exist as well [22].

With the rapid development of long-read sequencing technologies, such as Pacific Bioscience (PacBio) [23] and Oxford Nanopore Technology (ONT) [24] platforms, long-range spanning information provides the opportunity to more comprehensively detect SVs at a higher resolution [25]. However, novel computational approaches are required to well-handle the high sequencing error rates (typically 5–20%) and large lengths (over 10kbp on average) of the reads [26]. Mainly, two categories of approaches were employed in previous studies, i.e., de novo assembly-based and read alignment-based.

De novo assembly-based approaches [27–29] aim at assembling reads to longer genomic sequences (i.e., contigs and/or scaffolds) and discover SVs from the alignments between the assembled sequences and a reference. Such approaches are less influenced by the reference than that of read alignment-based approaches, especially free of read alignment artifacts. However, they are usually computational-intensive and still have some difficulties in the reconstruction of haplotype sequences of large genomes [30], which are also shortcomings to SV calling (a brief discussion is in the “Discussion” section).

Read alignment-based approaches directly align reads against the reference and detect SVs by analyzing the alignment results. Such approaches are more cost-effective to computational resources without lack of sensitivity and have been widely used in long read-based SV calling. Several read-alignment-based SV callers have been proposed, such as PB-Honey [31], SMRT-SV [32], Sniffles [33], PBSV (https://github.com/PacificBiosciences/pbsv), and SVIM [34]. They use various methods to find evidence of SVs implied by read alignments, such as the identification of local genomic regions with highly divergent alignments, the local assembly and re-alignment of clipped read parts, and the clustering of SV-spanning signatures [35]. Moreover, state-of-the-art long-read aligners, such as BLASR [36], NGMLR [33], Minimap2 [37], and PBMM2 (https://github.com/PacificBiosciences/pbmm2), were usually employed for read alignment.

However, read alignment-based SV calling is still non-trivial. Under the circumstance of high sequencing errors and complicated SVs, the alignment of the reads around SV breakpoints are chimeric and heterogeneous, usually less sensitive and accurate. Therefore, the SV signatures implied by read alignments are highly complicated, and it is difficult to collect and analyze them to implement sensitive detection for various kinds of SVs. Mainly, state-of-the-art tools have the following technical issues to be addressed: (1) overall, the sensitivity is still not satisfying (i.e., a high sequencing coverage is required and/or some SVs are still hard to detect); (2) some approaches (such as rMETL [38], rCANID [39], and npInv [40]) can only detect a subset or a particular class of SVs due to their specific designs; (3) some approaches (such as PBSV and SMRT-SV) are still time-consuming and do not have good scaling performance, which could be not suited to many large datasets; and (4) some approaches (such as SMRT-SV and PB-Honey) only support one type of sequencing data (e.g., for PacBio reads only), as they take the advantage of the characteristics of the data. These drawbacks are still the bottlenecks to the wide use of long-read sequencing data.

Herein, we present cuteSV, a versatile read-alignment-based SV detection approach having several beneficial features. (1) cuteSV has better SV detection yields than those of state-of-the-art SV callers. Especially, it has higher sensitivity for low coverage datasets without lack of accuracy. (2) cuteSV supports the datasets produced by mainstream long-read sequencing platforms with various error rates and can discover various types of SVs (including deletions, insertions, duplications, inversions, and translocations). (3) cuteSV has faster or comparable speed to state-of-the-art approaches with lower RAM usage. More importantly, it has outstanding scalability, i.e., enables to achieve almost linear speedup with the number of CPU threads. With these features, cuteSV is suited to large-scale data analysis tasks and has potentials to the cutting-edge genomics studies.

## Results

### Overview of cuteSV

Using sorted BAM file(s) as input, cuteSV extracts large insertions/deletions and split alignments in aligned reads as SV signatures and clusters and analyzes them to call SVs. The approach has three major steps as follows.

- Step 1: cuteSV uses multiple signature extraction methods to comprehensively collect the signatures of various types of SVs. Furthermore, the insertions and deletions are heuristically combined to recover the evidence of real SVs from fragile alignments.
- Step 2: cuteSV uses a specifically designed clustering-and-refinement approach to cluster the chimerically aligned reads in local regions and further refines the clusters to precisely distinguish the SV signatures from heterozygous SVs.
- Step 3: cuteSV uses several tailored rules to implement SV calling and genotyping based on the refined clusters of SV signatures.

**Fig. 1:**
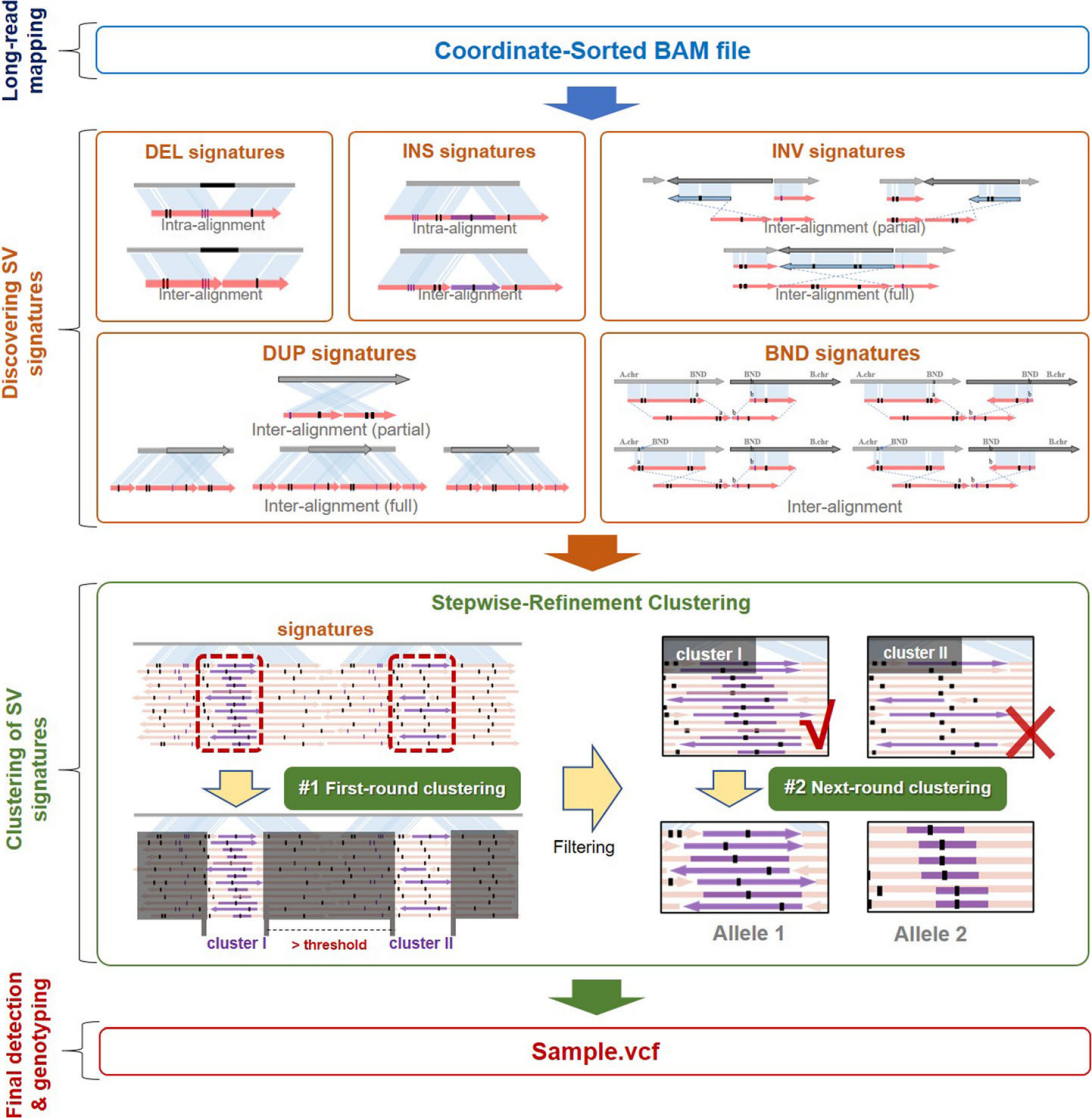
Schematic illustration of the cuteSV approach. cuteSV uses sorted BAM file as input to detect SVs in 3 major steps. In step 1 (“discovering SV signatures”), cuteSV collects various types of SV signatures comprehensively from inter- and intra-alignments. In step 2 (“clustering of SV signatures”), a heuristic clustering-and-refinement method is employed to sensitively discover accurate SV alleles. In step3 (“SV calling and genotyping”), cuteSV generates the SV callsets and assigns genotypes

A schematic illustration is in Fig. 1, and more details are in the “Materials and methods” section.

The major contribution of cuteSV approach is that it uses tailored heuristics to address three difficult technical issues in the alignment-based SV calling.

Firstly, due to the scoring systems of read aligners, some large SV events could be divided into several smaller insertions/deletions in a local region. Such cases are usually treated as multiple smaller SVs mistakenly. However, in step 1 of cuteSV, a heuristic method is used to combine insertions/deletions in nearby genomic regions to unbroken signatures of larger SVs. This method not only reduces the errors caused by the fragile read alignments, but also enables to produce more homogenous SV signatures from various reads, which is beneficial to the processing of later steps.

Secondly, reads spanning the same SV usually have heterogeneous breakpoints in their alignments, which also cause false-positive SV calls. In step 2 of cuteSV, the specifically designed clustering-and-refinement approach enables to adaptively cluster alignment breakpoints mapped to relatively large local regions but potentially belonging to identical SVs, so that heterogeneous breakpoints can be merged more effectively and more false positives can be prevented.

Thirdly, there are occasionally complex heterozygous SVs which multiple SV alleles are in same loci. Such SVs are difficult to detect and genotype. In step 2 of cuteSV, they are handled by a novel heuristic refinement on the SV signature clusters. That is, cuteSV investigates each of the clusters to check if there are heterogeneous signatures. If so, it implements a precise analysis to distinguish the reads potentially from various SV alleles and re-cluster them into more homogeneous sub-clusters. Taking the advantage of this approach, the complex SVs can be detected and genotyped correctly.

In addition, cuteSV uses a block division-based approach to process input data in a parallel way with multiple CPU threads. This implementation greatly helps it to achieve outstanding scaling performance, which has been demonstrated on several datasets in various sizes (see below for details).

### SV detection with simulated data

We used simulated datasets in various sequencing coverages to assess the “baseline” sensitivity and accuracy of cuteSV. More precisely, we collected 20,202 various types of known non-overlapping SVs, i.e., 6167, 9899, and 44 deletions, insertions, and inversions from CHM1 sample callsets [32] (nstd137 in dbVAR database), and 3712 and 380 duplications and translocations from KWS1 sample callsets [41] (nstd106 in dbVAR database), respectively. The various types of SVs were separately input to VISOR [42] with human reference genome (version: hs37d5) to generate five in silico donor genomes. And four PacBio-like datasets were simulated for each of the donor genomes (mean read length, 8000 bp; error model, default setting of PBSIM simulator [43]; coverages, 5×, 10×, 20×, and 30×, respectively); thus, 20 datasets were simulated in total. cuteSV and three state-of-the-art SV callers, i.e., Sniffles, PBSV, and SVIM, were implemented on the simulated datasets for comparison. Refer to the “Materials and methods” section for more details on the implementation of the simulation.

**Fig. 2:**
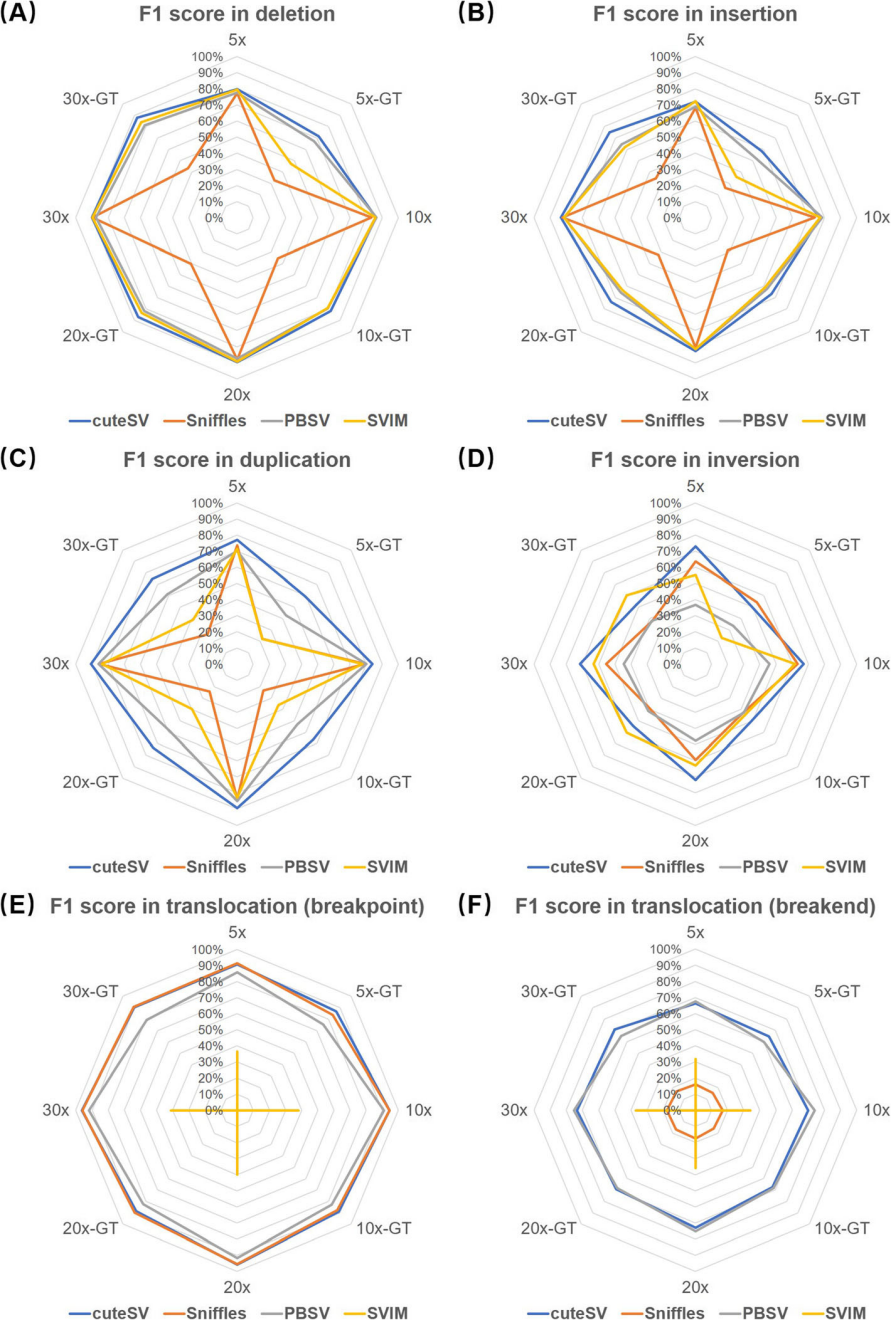
Benchmark results of the SV callers on various simulated datasets. F1 scores of **a** deletion, **b** insertion, **c** duplication, **d** inversion, **e** translocation at breakpoint level, and **f** translocation at breakend level, for the simulated datasets in various coverages and w/o genotyping. In the figure, “*N*×” and “*N*×-GT” indicate the statistics without and with genotyping, respectively

The benchmark results (Fig. 2a–c and Additional file 1: Table S1, S2, S3) indicate that cuteSV achieved highest F1 scores on the detection of deletions, insertions, and duplications for almost all the coverages w/o genotyping. It also had highest F1 scores for discovering inversions (Fig. 2d and Additional file 1: Table S4), but the best runner-up for genotyping due to slightly lower precision. For translocations, cuteSV and Sniffles were the best two callers at breakpoint level (Fig. 2e and Additional file 1: Table S5); meanwhile, cuteSV and PBSV showed higher F1 scores at breakend level (Fig. 2f and Additional file 1: Table S6) (refer to the “Materials and methods” section for the method of breakpoint- and breakend-level assessment for translocations). It is worth noting that SVIM did not assign genotypes for all the translocations and most of the duplications, which resulted in lower statistics.

Moreover, we assessed the accuracy of genotyping on the 30× simulated datasets with genotype confusion matrices (Additional file 1: Table S7). It is observed that cuteSV, PBSV, and SVIM recognized heterozygous and homozygous variants more accurately. Sniffles often misclassified homozygous variants as heterozygous especially for deletions, insertions, and duplications.

We also investigated the distinction of accurate calls of cuteSV made by various kinds of signatures (on the 30× simulated datasets, Additional file 1: Table S8). It shows that most of accurate insertion (93.59%) and deletion (96.18%) calls are produced with only CIGAR signatures, and the proportions of accurate calls produced with only split-alignment signatures and mixed signatures (CIGAR and split alignment) are low. This indicates that the employed aligner has a strong ability to handle deletions and insertions in reads to make informative CIGARs. It is also worth noting that this assessment was only done for insertions and deletions, since other types of SVs (inversions, duplications, and translocations) can only be detected based on split-alignment signatures.

Overall, the results suggest that cuteSV is a good versatile SV caller, i.e., it is able to sensitively detect various types of SVs without lack of accuracy, and their genotypes can be correctly recognized as well.

### SV detection with HG002 PacBio data

**Fig. 3:**
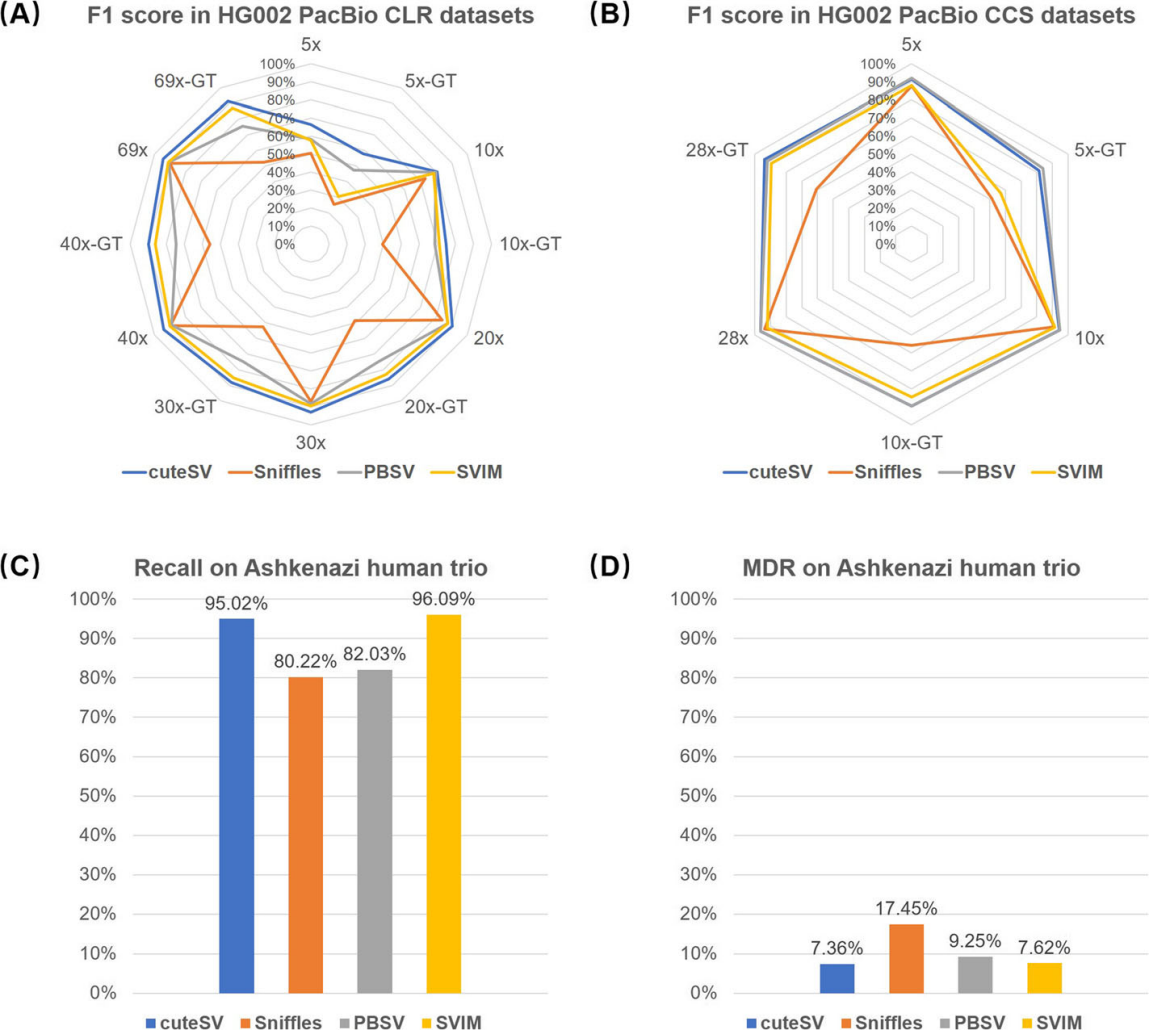
Benchmark results of the SV callers on various HG002 PacBio sequencing datasets. **a** Precisions, recalls, and F1 scores on the whole and down-sampled HG002 PacBio CLR datasets. **b** Precisions, recalls, and F1 scores on the whole and down-sampled HG002 PacBio CCS datasets. **c** Recall rate of homozygous parental variants. **d** Mendelian-Discordance-Rates (MDRs) for the variants unique to the offspring

We further benchmarked cuteSV, Sniffles, PBSV, and SVIM with several real sequencing datasets. The SV callers were implemented on a 69× HG002 PacBio CLR dataset [44] (mean read length, 7938 bp) at first. A high-confidence insertion and deletion callset for this sample made by Genome in a Bottle Consortium (GIAB) [45] was employed as the ground truth (since the callset of all types of SVs has still not been published by GIAB during the submission of the manuscript). Truvari (https://github.com/spiralgenetics/truvari) was used to assess the precision, recall, and F1 score of the callsets produced by various callers. Their yields are shown in Fig. 3a and Additional file 1: Table S9. cuteSV simultaneously achieved the highest precision, recall, and F1 score, all of which were > 94% in absolute terms, which is feasible for practical use. The F1 scores of SVIM, PBSV, and Sniffles were slightly lower, mainly due to their lower recall statistics (i.e., 89.56%, 88.42%, and 86.27%, respectively). For SV genotyping, cuteSV achieved > 90% recall and F1 score. SVIM was the best runner-up, and the statistics of PBSV and Sniffles were obviously lower.

We further randomly down-sampled the dataset to 5×, 10×, 20×, 30×, and 40× to assess the ability of the SV callers on lower coverage datasets (Fig. 3a and Additional file 1: Table S9). cuteSV still achieved higher precisions, recalls, and F1 scores on almost all the datasets. Especially, it achieved > 90% F1 score and > 85% GT-F1 score at 20×, indicating that more cost-effective sequencing plans (lower coverages) could be feasible with cuteSV. However, this is still difficult for other callers since their precisions, recalls, and F1 scores were decreased on the down-sampled datasets.

We also assessed the ability of the callers on a PacBio CCS dataset of the same sample [29, 46] (coverage: 28×, mean read length: 13478 bp). The results of cuteSV and PBSV were very close to each other (Fig. 3b and Additional file 1: Table 10) (precision, 94.6%; recall, 98.0%; F1 score, 96.3%), and they outperformed Sniffles and SVIM by 1 to 6% on various statistics. cuteSV also outperformed the other three callers by 2 to 33% on GT-F1 score. We randomly down-sampled this dataset to 5× and 10× for further assessment (Fig. 3b and Additional file 1: Table S10). On the 10× dataset, the F1 score of PBSV is 0.37% higher than that of cuteSV due to its highest recall (96.70% vs. 93.79%). The lower recall of cuteSV was mainly due to that the minimal signature size parameter setting of cuteSV (default value, 30 bp) was larger than that of PBSV (default value: 20 bp), which hindered cuteSV in identifying more SVs having smaller sizes. Moreover, it was observed that both of cuteSV and PBSV achieved > 90% precision, recall, and F1 score at 5×, suggesting that the required coverage for SV calling can be lower with high sequencing quality.

To assess the ability to detect various types of SVs (i.e., insertions, deletions, inversions, duplications, and translocations) more comprehensively, we further employed GIAB Ashkenazi Trio PacBio CLR datasets (HG002, HG003, and HG004) to assess the recall rates and Mendelian-Discordance-Rates (MDRs). cuteSV and SVIM obtained > 95% mean recall rate, i.e., more than 95% homozygous parental SVs has been confirmed in the offspring (Fig. 3c and Additional file 1: Table S11). cuteSV was 1% lower than SVIM on recall rate; however, we realized that this does not mean a lower sensitivity of cuteSV, but due to that about 15% SVs in parental callsets discovered by SVIM had no genotypes so that they cannot be assessed and decreased the total number of homozygous parental SVs. Meanwhile, the MDR of cuteSV is lowest (7.36%, Fig. 3d and Additional file 1: Table S11), indicating that its callsets were more plausible. The MDRs of SVIM (7.62%) and PBSV (9.25%) are also comparable and much lower than that of Sniffles (17.45%).

### SV detection with HG002 ONT PromethION data

**Fig. 4:**
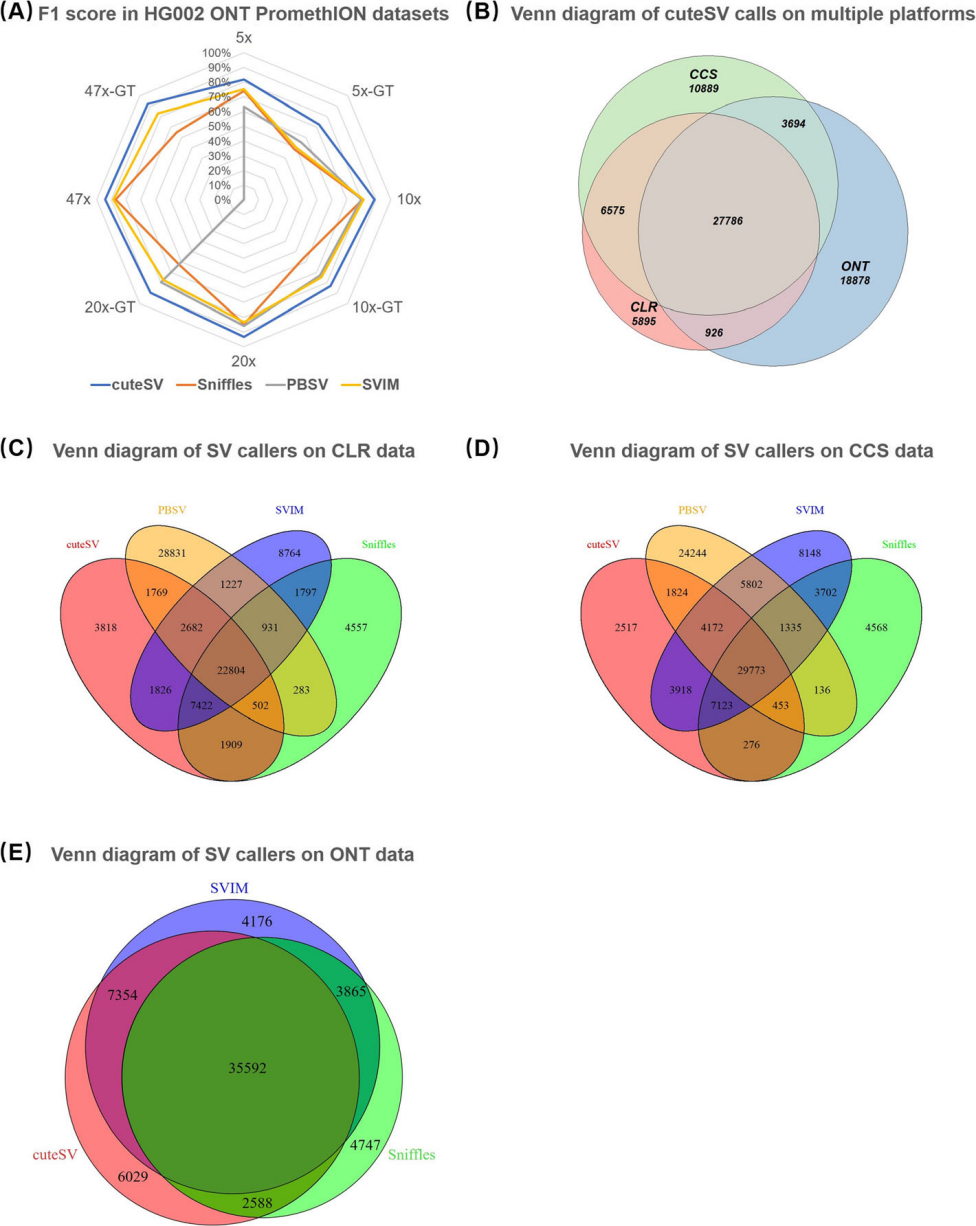
Benchmark results of the SV callers on various of HG002 ONT sequencing datasets. **a** Precisions, recalls, and *F*-scores on the whole and down-sampled HG002 ONT datasets. **b** The Venn diagram of SV calls produced by cuteSV from HG002 PacBio CLR, CCS, and ONT PromethION datasets (indicated by “CLR”, “CCS,” and “ONT”, respectively). **c** The Venn diagram of SV calls produced by different tools on HG002 PacBio CLR data. **d** The Venn diagram of SV calls produced by different tools on HG002 PacBio CCS data. **e** The Venn diagram of SV calls produced by different tools on HG002 ONT PromethION data

A newly published ONT PromethION dataset of the HG002 sample is used for benchmarking the SV callers on ONT data (mean read length, 17335 bp; coverage, 47×, available at ftp://ftp.ncbi.nlm.nih.gov/giab/ftp/data/AshkenazimTrio/HG002_NA24385_son/UCSC_Ultralong_OxfordNanopore_Promethion/). The results (Fig. 4a and Additional file 1: Table S12) suggest that the outperformance of cuteSV (precision, 92.14%; recall, 96.61%; and F1 score, 94.32%) was more obvious than that of the PacBio CLR dataset. It is also worth noting that PBSV crashed for this dataset. On the randomly down-sampled datasets (5×, 10×, and 20×), the outperformance of cuteSV was notable as well. Particularly, cuteSV detected most (85%) of the ground truth SVs only at 10× coverage with high precision (93.07%) and F1 score (88.85%), while the F1 scores of Sniffles, SVIM, and PBSV were at least 7% lower at the same coverage. Moreover, the GT-F1 score of cuteSV was at least 9% higher as well.

The SV callsets produced by cuteSV from the HG002 PacBio CLR, PacBio CCS and ONT PromethION datasets were compared (having 42,732, 50,056, and 51,563 > 30 bp SV calls, respectively, Fig. 4b, Additional file 1: Fig. S1 and Table S13). Five thousand eight hundred ninety-five SVs (13.80%) of the PacBio CLR callset, 10,889 SVs (21.75%) of the PacBio CCS, and 18,878 SVs (36.61%) of the ONT callset are unique. It is worth noting that 67.18% (3960 of 5895) CLR-only calls and 82.34% (15,545 of 18,878) ONT-only calls are insertions and deletions, respectively. The numbers coincide with previous studies [23] that more false-positive insertion and deletion calls are likely to be produced with PacBio CLR and ONT datasets, respectively [24]. Moreover, it is also observed that ONT data is more useful to discover large insertions than PacBio CLR data, mainly because of larger read lengths. An example is shown in Additional file 1: Fig. S2. A 6481-bp insertion (breakpoint at chr1:9683994) was only detected in the ONT reads, possibly due to that the PacBio reads in this region are shorter and the aligners cannot align them with such a large insertion, while a large proportion of ONT reads carry significant insertion signals in their CIGARs.

Moreover, Venn diagrams of the SV callsets produced by various approaches on the HG002 PacBio CLR, PacBio CCS, and ONT PromethION datasets were plotted (Fig. 4c–e and Additional file 1: Table S14). For PacBio CLR and CCS data, there are 22,804 and 29,733 > 30-bp SV calls made by all the approaches, respectively, and cuteSV has fewer unique calls (3818 for CLR and 2517 for CCS data) than other tools. For ONT PromethION data, only the callsets of cuteSV, Sniffles, and SVIM were considered, and 35,592 > 30-bp SV calls were made by all of the three tools; the trend is similar to that of PacBio data. However, the number of unique calls by cuteSV (i.e., 6029 of 51,563) is slightly higher than that of the other two methods. This is mainly due to that cuteSV detected more deletions.

### The performance of the SV callers

**Fig. 5:**
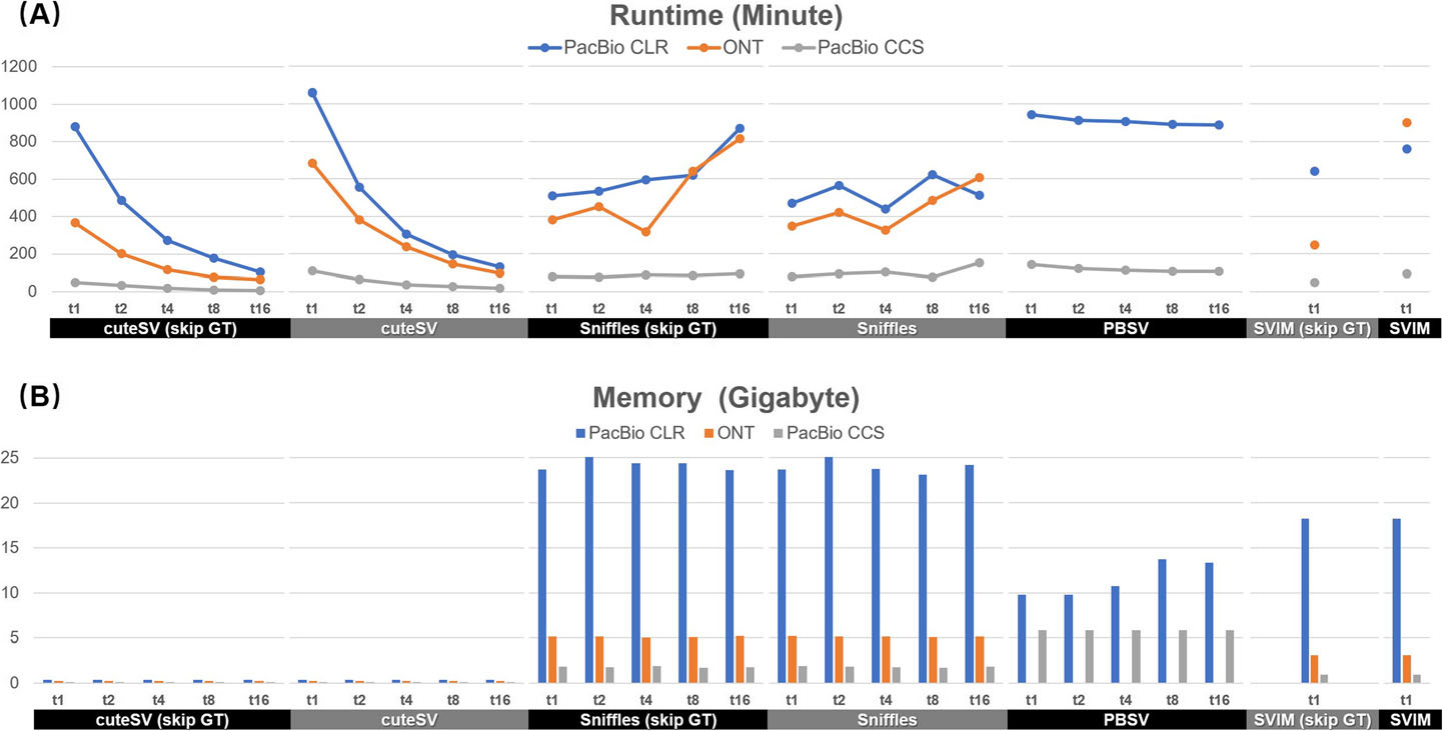
Performance of the benchmarked SV callers. The **a** runtimes and **b** memory footprints of cuteSV, cuteSV, Sniffles, and PBSV with 1, 2, 4, 8, and 16 CPU threads. “Skip GT” indicates the statistics without genotyping. SVIM was benchmarked with single CPU thread only since it does not support multiple thread computing

We assessed the speed and memory footprint of the SV callers on the three HG002 datasets (Fig. 5 and Additional file 1: Table S15). Generally, using a single CPU thread, Sniffles with genotyping was faster than other benchmarked SV callers, i.e., Sniffles (without genotyping), SVIM (w/o genotyping), cuteSV (w/o genotyping), and PBSV (genotyping cannot be skipped). It is also worth noting that the genotyping step of cuteSV costs 17 to 55% of the time on various datasets, since it needs to re-check all the reads around the detected SV breakpoints to infer SV genotypes. When using multiple CPU threads, only cuteSV has high scaling performance, i.e., it achieved a quasi linear speedup with the number of CPU threads and its wall clock time was greatly reduced. Neither Sniffles nor PBSV had an obvious speedup with more CPU threads while SVIM does not support multiple-thread computing. In this situation, cuteSV is faster than all the other callers when using 4 or more threads. On memory footprint, cuteSV (about 0.1GB to 0.4GB) was smaller than that of other approaches by one or two orders of magnitude. With its quasi linear multiple-threads speedup and low memory footprint, we realized that cuteSV is a highly scalable SV detection tool, which is suited to high-performance computing platforms and large-scale data analysis tasks such as SV detection in many samples.

### The effects of various read aligners on SV calling

We further employed PBMM2 and NGMLR to separately align the reads of the 69× HG002 PacBio CLR dataset to produce various callsets. Mainly, two issues were observed.

1. The false negatives (FNs) of the callsets with various aligners are shown in Additional file 1: Fig. S3A and Table S16. With PBMM2, cuteSV, Sniffles, PBSV, and SVIM discovered 134, 200, 404, and 55 more < 1-kbp SVs, respectively, indicating that PBMM2 is more helpful to the detection of < 1-kbp insertions and deletions. Notably, PBSV discovered 369 more insertions (size between 200 and 700 bp) with PBMM2, which are coincided with the sequences of the Alu family. On the other hand, NGMLR seems better for the detection of > 1-kbp SVs, as 109, 283, 127, and 113 more such SVs were discovered by the four callers respectively.
2. The false positives (FPs) of the callsets with PBMM2 and NGMLR are shown in Additional file 1: Fig. S3B and Table S17. cuteSV, Sniffles, and PBSV with PBMM2 had slightly higher numbers (155, 54, and 43, respectively) of < 1-kbp FP insertions and deletions, while SVIM had a higher number (45) of FPs with NGMLR. For > 1-kbp SVs, the numbers of FPs were very close for the corresponding callsets of cuteSV, PBSV, and SVIM, but there were 32 more FPs in the callset of Sniffles with NGMLR than that of PBMM2.

### The results of cuteSV with various configurations of parameters

We assessed the yields of cuteSV with various configurations of two critical parameters, i.e., --min_support and --min_size (-s and -l in the software, respectively), on various coverages (5×, 10×, 20×, 30×, 40×, and 69×) of HG002 CLR datasets.

The --min_support parameter indicates the minimal number of supporting reads to make an SV call. As the results displayed in Additional file 1: Fig. S4A and Table S18 show, with the default setting of --min_size (--min_size = 30), cuteSV achieved the best yields when --min_support was configured as 1 to 10 for the various coverages. And there is an obvious trade-off between precision and recall, that is, setting a smaller --min_support value might result in higher sensitivity but lower precision, and vice versa.

The --min_size parameter indicates the minimal size of SV signature considered in clustering. Keeping the --min_support parameter fixed, we investigated the results of cuteSV with two different settings for --min_size (i.e., --min_size = 30 and --min_size = 50, Additional file 1: Fig. S4B). At various coverages, the accuracies of cuteSV were 0.12 to 1.38% higher with the setting of --min_size = 50, while the recall rates were − 0.06 to 1.09% higher with --min_size = 30. This indicates that setting --min_size with smaller numbers might result in higher sensitivity but lower accuracy, and vice versa. It is also worth noting that, although the trade-off exists, for each coverage, the F1 scores of cuteSV with various settings are quite close to each other (the difference is less than 1%).

### SV detection with NA19240 PacBio CLR data

A PacBio CLR dataset from another well-studied human sample (NA19240) [47] was employed (mean read length, 6503 bp; coverage, 40×) to benchmark the SV callers more comprehensively. We collected a callset from a previous study [48] for this sample and considered it as the ground truth. It contains a total number of 37,657 SVs (size > 50 bp), including 17,950 deletions, 19,482 insertions/duplications, and 225 inversions.

The precisions, recalls, and F1 scores of the benchmarked SV callers are shown in Additional file 1: Fig. S5 and Table S19. cuteSV had the highest F1 scores for all types of SVs (i.e., DEL, 63.37%; INS/DUP, 56.50%; INV, 13.22%; and all types, 59.36%). Sniffles and PBSV had lower sensitivities or/and accuracies than that of cuteSV. SVIM outperformed cuteSV on sensitivity by 6% for all types. However, this is partially due to its larger number of predictions (30,024 vs. 21,721, surpassed cuteSV by 38%); meanwhile, cuteSV showed a 9% higher precision than that of SVIM.

## Discussion

Long-read sequencing technologies are promising to discover the SVs of sequenced sample comprehensively. However, it is still non-trivial to exert the advantages of long reads due to the high sequencing errors and the complexity of SVs. Herein, we propose cuteSV, a novel read alignment-based SVs calling approach, to show how to achieve higher yields, speed, and scaling performance in SV calling with tailored signature extraction, clustering, and refinement methods.

Mainly, the proposed approach has four advantages as follows.

1. The heuristic SV signature extraction methods enable to well-handle the fragile read alignments around SV breakpoints, which helps to build high-quality SV evidence. This is one of the major reasons that cuteSV outperforms state-of-the-art tools. An example is in Additional file 1: Fig. S6. It shows how a 272-bp insertion event was successfully discovered by cuteSV, while other benchmarked SV callers were affected by the multiple insertions and deletions in read alignments.
2. The clustering-and-refinement method enables to distinguish the reads from the multiple SV alleles in the same loci and generate correct signature clusters. Thus, complex heterozygous SVs can be detected and genotyped sensitively and accurately, which also outperforms state-of-the-art tools. Two examples are shown in Additional file 1: Fig. S7. One is a heterozygous insertion event (a 108-bp and a 36-bp insertion in the same region), and the other is a heterozygous deletion event (a 123-bp and a 37-bp deletion in the same region). Only cuteSV and PBSV successfully discovered them.
3. With its novel heuristic methods, cuteSV improves the sensitivity of SV calling especially for lower coverage datasets. The benchmark results suggest that cuteSV can discover most of the SVs in 20× coverage datasets for human samples without loss of accuracy; meanwhile, the performance of genotyping is consistent with its yields as well. This is helpful to make more flexible and cost-effective sequencing plans in large-scale genomics studies.
4. With its block division-based implementation, cuteSV has outstanding scaling performance, which enables to achieve a nearly linear multiple-thread speedup during data processing. This is very suited to modern HPC resources and helpful for large-scale genomics studies.

However, from the view of alignment-based SV calling, there are still a few shortcomings for cuteSV as follows, which are also important future works to further improve the approach.

1. There are still some SVs that cannot be successfully detected by cuteSV. We investigated the intermediate results of cuteSV and found that most of the false-negative calls were due to the read alignments being inaccurate or not informative enough. Two typical examples are shown in Additional file 1: Fig. S8 and S9. In Additional file 1: Fig. S8, a 98-bp deletion case is shown in which deletion signatures emerge in nearly all the reads around the event. However, the size of the deletions in the alignment are not correct (i.e., most of them are around 50 bp). In Additional file 1: Fig. S9, a 707-bp insertion case is shown in which insertion signatures also emerge and their sizes are close to the SV event, but the positions of the breakpoints in the reads are quite far from the ground truth breakpoint. Under such circumstances, all the benchmarked SV callers made SV calls, but the sizes and/or positions are incorrect since the read alignments are misleading.
2. The current version of cuteSV is tailored for human genomes or diploid genomes. For multi-ploidy genomes such as many plant genomes, cuteSV can also extract the signatures to detect SVs; however, genotyping is still implemented with the diploid model. Such tasks are still non-trivial to cuteSV (as well as other state-of-the-art SV calling approaches), since there could be more heterozygous SVs and less SV supporting reads (as more alleles exist), and the genotypes of SVs are also more complicated as well. With the current implementation, cuteSV could miss some SV alleles in multi-ploidy genomes due to low numbers of supporting reads; meanwhile, it may make mistakes in genotyping. This is an important work for us to develop more sensitive SV signature extraction methods as well as specifically designed genotyping model to the SV detection of multi-ploidy genomes.
3. Although it has higher scaling performance, the speed of cuteSV in single thread is still slower than some of state-of-the-art approaches. This is mainly due to that the SV signature extraction of cuteSV is more complicated. An optimization on the data structures and operations of cuteSV could be feasible to further reduce the time cost.

Moreover, from a higher-level view, de novo assembly-based approaches have their own advantages to cuteSV as well as other alignment-based SV callers. Firstly, although the mappability of long reads is much higher than that of short reads, their alignments are still heterogeneous and have potential errors due to many factors, such as sequencing errors, SV complexity, and repetitive sequences. However, de novo assembly-based approaches are free of such alignment artifacts so that they could have less systematic errors. Secondly, de novo assembly-based approaches are able to well-handle large novel insertions by assembling them as distinctive contigs; however, this type of SV is still non-trivial to alignment-based approaches. Thirdly, haplotype-resolved assembly inherently helps to unravel the haplotype configuration of SVs, which is useful to many downstream analyses.

However, de novo assembly-based approaches also have some non-neglectable bottlenecks. Firstly, the assembly could be not complete, i.e., some of the haplotype sequences (especially contigs of SV alleles) are missing, which may affect the sensitivity of SV calling. Secondly, assembly mistakes still exist, which may cause false positives. Thirdly, de novo assembly is still computational-intensive, which is not cost-effective for large-scale data analysis tasks. Fourthly, it usually needs high-coverage sequencing or the combination of multiple types of data (such as short reads, long reads, Hi-C and optical map) to implement haplotype-resolved assembly [49]; however, most of SVs can be detected with lower coverage data by alignment-based approaches like cuteSV. Considering their own advantages and shortcomings, we consider that alignment- and assembly-based approaches are complementary to each other, and it could be useful to integrate these two approaches to produce SV callsets with higher quality.

## Conclusion

In this article, we propose a novel long-read-based SV detection approach, cuteSV. It enables the thorough analysis of the complex signatures of SVs implied by read alignments. Benchmark results demonstrate that cuteSV achieves good yields and performance simultaneously. Especially, it has good sensitivity to detect SVs, even with low coverage sequencing data, and it also has outstanding scaling performance which is suited to handle many large datasets. We believe that cuteSV has the potentials to cutting-edge genomics studies.

## Materials and methods

Some details on the implementations of cuteSV approach and the benchmark are as follows. Please also refer to Additional file 1: Table S20 for a nomenclature.

### Read alignment

cuteSV uses sorted BAM files as input, and it is supported by employing state-of-the-art long-read aligners to compose SV detection pipelines. Aligners with a good ability to handle large insertions/deletions in reads and/or produce accurate split alignments are preferred, since cuteSV extracts important SV signatures from such alignments. In the “Results” section, it is demonstrated that state-of-the-art aligners such as PBMM2, NGMLR, and Minimap2 are suited to cuteSV.

### Extraction of SV signatures implied by CIGARs

Given a set of aligned reads, cuteSV separately analyzes the detailed alignment of each read. Mainly, it extracts two categories of SV signatures, i.e., long insertions/deletions in CIGARs and split alignments. The signatures of SVs are represented as 3-tuples (i.e., Sig = (Ref_s_, SV_*L*_, Read_ID_), where Ref_s_ indicates the starting coordinate on the reference genome, SV_*L*_ indicates the size of SV, and Read_ID_ indicates the unique read ID). cuteSV clusters and analyzes Sigs of various reads to call SVs.

cuteSV uses a heuristic method to extract and merge the long insertions/deletions in CIGARs during SV signature extraction. In details, cuteSV extracts insertions/deletions > 30 bp in size as described by the CIGARs of the reads and composes them into Sigs with their positions, lengths, and read IDs. For two signatures, *Sig*_1_ and *Sig*_2_, cuteSV merges them if they meet the following condition:

1 $$ \left\{\begin{array}{c}{\mathrm{Ref}}_{2s}-{\mathrm{Ref}}_{1s}\le {\mathrm{Sig}}_{\mathrm{dis}}\ \mathrm{if}\ \mathrm{INS}\\ {}{\mathrm{Ref}}_{2s}-\left({\mathrm{Ref}}_{1s}+{\mathrm{SV}}_{1L}\right)\le {\mathrm{Sig}}_{\mathrm{dis}}\ \mathrm{if}\ \mathrm{DEL}\end{array}\right. $$

where Sig_dis_ is a threshold of the distance between the two Sigs. The two signatures are then merged *as* Sig_M_ = (Ref_1s_, SV_1*L*_ + SV_2*L*_, Read_ID_). This method enables to recover the SV signatures of long insertions/deletions which are initially partitioned as multiple trivial indels by read aligners. Further, all the remaining signatures after merging are used as informative signatures.

### Extraction of SV signatures implied by split alignments

For a read having split alignments (described by its primary and supplementary alignments), cuteSV records each split by a 6-tuple Seg = (Read_s_, Read_e_, Ref_s_, Ref_e_, Chr, Strand) (also termed as a “segment”), where Read_s_, Read_e_, Ref_s_, Ref_e_ respectively indicate the starting and end coordinates on the read and reference genome, and *Chr* and *Strand* respectively indicate its chromosome and orientation. cuteSV uses the following heuristic rules to recover SV signatures from Segs.

1. Extraction of deletion/insertion signatures. If two segments, Seg_1_ and Seg_2_, are adjacent on the read and aligned to the same chromosome with identical orientations, cuteSV computes Diff_dis_ = (Ref_2s_ − Ref_1e_) − (Read_2s_ − Read_1e_) and Diff_olp_ = Ref_1e_ − Ref_2s_. If Diff_olp_ < 30 bp and Diff_dis_ ≥ 30 *bp*, cuteSV considers that the two segments indicate a deletion event and composes a deletion signature:

2 $$ {\mathrm{Sig}}_{\mathrm{DEL}}=\left({\mathrm{Ref}}_{1\mathrm{e}},{\mathrm{Diff}}_{\mathrm{dis}},{\mathrm{Read}}_{\mathrm{ID}}\right) $$

Moreover, if Diff_olp_ < 30 bp and Diff_dis_ ≤  − 30 *bp*, cuteSV considers it as an insertion event and an insertion signature is composed:

3 $$ {\mathrm{Sig}}_{\mathrm{INS}}=\left(\frac{{\mathrm{Ref}}_{1\mathrm{e}}+{\mathrm{Ref}}_{2\mathrm{s}}}{2},-{\mathrm{Diff}}_{\mathrm{dis}},{\mathrm{Read}}_{\mathrm{ID}}\right) $$

(2) Extraction of duplication signatures. If two adjacent segments are mapped to similar positions (i.e., Diff_olp_ ≥ 30 bp) and in identical orientations, cuteSV composes a duplication signature:

4 $$ {\mathrm{Sig}}_{\mathrm{DUP}}=\left({\mathrm{Ref}}_{2\mathrm{s}},{\mathrm{Ref}}_{1\mathrm{e}},{\mathrm{Read}}_{\mathrm{ID}}\right) $$

(3) Extraction of inversion signatures. If two adjacent segments are mapped to the same chromosome but to different strands, cuteSV composes an inversion signature:

5 $$ {\mathrm{Sig}}_{\mathrm{INV}}=\left\{\begin{array}{c}\left(\min \left({\mathrm{Ref}}_{1\mathrm{e}},{\mathrm{Ref}}_{2\mathrm{e}}\right),\max \left({\mathrm{Ref}}_{1\mathrm{e}},{\mathrm{Ref}}_{2\mathrm{e}}\right),{\mathrm{INV}}_{\mathrm{hh}},{\mathrm{Read}}_{\mathrm{ID}}\right),\mathrm{if}\ {\mathrm{Strand}}_1\ \mathrm{is}+\\ {}\left(\min \left({\mathrm{Ref}}_{1\mathrm{s}},{\mathrm{Ref}}_{2\mathrm{s}}\right),\max \left({\mathrm{Ref}}_{1\mathrm{s}},{\mathrm{Ref}}_{2\mathrm{s}}\right),{\mathrm{INV}}_{\mathrm{tt}},{\mathrm{Read}}_{\mathrm{ID}}\right),\mathrm{if}\ {\mathrm{Strand}}_1\ \mathrm{is}-\end{array}\right. $$

where INV_hh_ and INV_tt_ indicate head-to-head inversion signal and tail-to-tail inversion signal, respectively.

(4) Extraction of translocation signatures. If two adjacent segments are mapped to different chromosomes, and the two segments are < 100 bp distant on the reads, cuteSV composes a translocation signature:

6 $$ {\mathrm{Sig}}_{\mathrm{BND}}=\left\{\begin{array}{c}\left({\mathrm{Chr}}_1,{\mathrm{Ref}}_{1\mathrm{e}},{\mathrm{Chr}}_2,{\mathrm{Ref}}_{2\mathrm{s}},{\mathrm{Read}}_{\mathrm{ID}}\right),\mathrm{if}\ {\mathrm{Chr}}_1<{\mathrm{Chr}}_2\ \mathrm{and}++\\ {}\left({\mathrm{Chr}}_2,{\mathrm{Ref}}_{2\mathrm{s}},{\mathrm{Chr}}_1,{\mathrm{Ref}}_{1\mathrm{e}},{\mathrm{Read}}_{\mathrm{ID}}\right),\mathrm{if}\ {\mathrm{Chr}}_2<{\mathrm{Chr}}_1\ \mathrm{and}++\\ {}\left({\mathrm{Chr}}_1,{\mathrm{Ref}}_{1\mathrm{e}},{\mathrm{Chr}}_2,{\mathrm{Ref}}_{2\mathrm{e}},{\mathrm{Read}}_{\mathrm{ID}}\right),\mathrm{if}\ {\mathrm{Chr}}_1<{\mathrm{Chr}}_2\ \mathrm{and}+-\\ {}\left({\mathrm{Chr}}_2,{\mathrm{Ref}}_{2\mathrm{e}},{\mathrm{Chr}}_1,{\mathrm{Ref}}_{1\mathrm{e}},{\mathrm{Read}}_{\mathrm{ID}}\right),\mathrm{if}\ {\mathrm{Chr}}_2<{\mathrm{Chr}}_1\ \mathrm{and}+-\\ {}\left({\mathrm{Chr}}_1,{\mathrm{Ref}}_{1\mathrm{s}},{\mathrm{Chr}}_2,{\mathrm{Ref}}_{2\mathrm{s}},{\mathrm{Read}}_{\mathrm{ID}}\right),\mathrm{if}\ {\mathrm{Chr}}_1<{\mathrm{Chr}}_2\ \mathrm{and}-+\\ {}\left({\mathrm{Chr}}_2,{\mathrm{Ref}}_{2\mathrm{s}},{\mathrm{Chr}}_1,{\mathrm{Ref}}_{1\mathrm{s}},{\mathrm{Read}}_{\mathrm{ID}}\right),\mathrm{if}\ {\mathrm{Chr}}_2<{\mathrm{Chr}}_1\ \mathrm{and}-+\\ {}\left({\mathrm{Chr}}_1,{\mathrm{Ref}}_{1\mathrm{s}},{\mathrm{Chr}}_2,{\mathrm{Ref}}_{2\mathrm{e}},{\mathrm{Read}}_{\mathrm{ID}}\right),\mathrm{if}\ {\mathrm{Chr}}_1<{\mathrm{Chr}}_2\ \mathrm{and}--\\ {}\left({\mathrm{Chr}}_2,{\mathrm{Ref}}_{2\mathrm{e}},{\mathrm{Chr}}_1,{\mathrm{Ref}}_{1\mathrm{s}},{\mathrm{Read}}_{\mathrm{ID}}\right),\mathrm{if}\ {\mathrm{Chr}}_2<{\mathrm{Chr}}_1\ \mathrm{and}--\end{array}\right. $$

where < means the first chromosome is alphabetically smaller than the second chromosome and “++”, “+−”, “−+”, “--” indicate the combination of strands for an inter-chromosomal SV.

(5) cuteSV uses a specifically designed method to extract the signatures of a complex kind of SV, when there is a mobile insertion in between two duplicated sequences. An example is shown in Additional file 1: Fig. S10, in which there are two duplicated local sequences (both of their mapped positions are around Chr1:73594981), and there is another local sequence within them in the read (whose mapped position is in a decoy sequence of hs37d5). cuteSV extracts a series of signatures, including a duplication (as shown in Eq.4), a translocation (as shown in Eq.6), and an insertion (as shown in Eq.7), to describe such complex SV events.

7 $$ {\mathrm{Sig}}_{\mathrm{INS}}=\left(\min \left({\mathrm{Ref}}_{1\mathrm{e}},{\mathrm{Ref}}_{2\mathrm{s}}\right),-{\mathrm{Diff}}_{\mathrm{dis}},{\mathrm{Read}}_{\mathrm{ID}}\right) $$

### Clustering-and-refinement of SV signatures

cuteSV uses a two-step approach to cluster SV signatures into bins, where each bin has a set of SIGs belonging to a specific SV allele. In the first step, it clusters the signatures by their genomic positions and types in order to bin the signatures in various local regions. In the second step, it refines the clusters of signatures by their length in order to distinguish the signatures of the various alleles of complex heterozygous SVs (i.e., a complex heterozygous SV has multiple similar SV alleles at the same locus).

In the first step, cuteSV sorts all the SV signatures by their genomic coordinates and types (i.e., deletions, insertions, duplications, inversions, and translocations). For each category, cuteSV initially creates a new cluster and scans all the signatures from upstream to downstream and adds them into the cluster using an iterative approach. More precisely, for a newly scanned SV signature SIG_*i*_, cuteSV adds it into the cluster if there is at least one signature SIG_*j*_ in the cluster which meets the following condition:

8 $$ {\displaystyle \begin{array}{c}{\mathrm{Sig}}_i\left(\mathrm{pos}\right)-{\mathrm{Sig}}_{\mathrm{j}}\left(\mathrm{pos}\right)\le {\mathrm{TH}}_{\mathrm{type}}\ \mathrm{or}\\ {}\left|\left({\mathrm{Sig}}_i\left(\mathrm{pos}\right)+{\mathrm{Sig}}_i\left(\mathrm{len}\right)\right)-\left({\mathrm{Sig}}_{\mathrm{j}}\left(\mathrm{pos}\right)+{\mathrm{Sig}}_{\mathrm{j}}\left(\mathrm{len}\right)\right)\right|\le {\mathrm{TH}}_{\mathrm{type}}\end{array}} $$

where TH_type_ is a threshold of the distances among the clustered signatures, and different values are used for various types of SVs (TH_type_ is typically configured to between 50 and 500 bp). If Sig_*i*_ cannot be added into the cluster, cuteSV creates a new cluster only having Sig_*i*_ and goes to the next SV signature.

In the second step, cuteSV discards the clusters with too few signatures, and the remaining clusters are refined by various methods according to their SV type.

1. Refinement of deletion/insertion clusters. Given a deletion or insertion cluster, cuteSV sorts all its signatures by their sizes and computes a parameter, Bias_*L*_, with the following equation:

9 $$ {\mathrm{Bias}}_L=\alpha \times \frac{1}{\left|{\mathrm{Group}}_{\mathrm{Sig}}\right|}{\sum}_{1\le k\le \left|{\mathrm{Group}}_{\mathrm{Sig}}\right|}{\mathrm{Len}}_k $$

where *α* is a weighting parameter, |Group_Sig_| is the number of the signatures in the cluster, and Len_*k*_ is the length of the *k*-th longest signature in Group_Sig_. The configuration of *α* depends on SV types, i.e., the default value of *α* is 0.2 for an insertion cluster and high error rate reads (PacBio CLR and ONT reads), *α* = 0.65 for low error rate reads (e.g., PacBio CCS reads), and *α* = 0.3 for a deletion cluster (regardless of error rate).

Using Bias_*L*_, cuteSV divides the cluster into sub-clusters of which each is a potential SV allele in a local genomic region. It initially adds the signatures with the largest size into a new sub-cluster and then iteratively scans the signatures by size (from largest to smallest). A newly scanned signature is added into the sub-cluster if the difference between its size and that of the last signature added to the sub-cluster is smaller than Bias_*L*_. Otherwise, a new sub-cluster is created.

cuteSV recognizes the generated sub-clusters with the highest number of signatures as a “major allele” sub-cluster if it meets the following conditions:

10 $$ \mathrm{SR}\ge {\mathrm{SR}}_{\mathrm{min}}\&\&\mathrm{SR}>\mu \times \left|{\mathrm{Group}}_{\mathrm{Sig}}\right| $$

where *SR* and *SR*_*min*_ are the number of its supporting reads and the threshold of the minimum number of supporting reads, respectively. *μ* is a weighting parameter. For an insertion cluster, its default value is 0.6 (for PacBio CLR or ONT reads) or 0.65 (for PacBio CCS reads). For a deletion cluster, the default values are 0.7 and 0.35 for the data types mentioned above, respectively. If a major allele sub-cluster exists, cuteSV recognizes the remaining sub-clusters having more than SR_min_ signatures as “minor allele” sub-clusters.

There is occasionally no major allele sub-cluster due to lack of enough supporting reads. cuteSV uses another heuristic rule in which it recognizes the two largest sub-clusters SR_first_ and SR_second_ as major allele and minor allele sub-clusters if they meet the following conditions:

11 $$ \left\{\begin{array}{c}{\mathrm{SR}}_{\mathrm{min}}\le {\mathrm{SR}}_{\mathrm{first}}\le \mu \times \left|{\mathrm{Group}}_{\mathrm{Sig}}\right|\\ {}0.4\times \left|{\mathrm{Group}}_{\mathrm{Sig}}\right|\le {\mathrm{SR}}_{\mathrm{second}}\le {\mathrm{SR}}_{\mathrm{min}}\\ {}0.95\times \left|{\mathrm{Group}}_{\mathrm{Sig}}\right|\le {\mathrm{SR}}_{\mathrm{first}}+{\mathrm{SR}}_{\mathrm{second}}\end{array}\right. $$

This rule indicates that almost all of the SV signatures support the two alleles; meanwhile, both of them occupy > 40% of the supporting signatures in the cluster.

(2) Refinement of duplication/inversion clusters. Given a duplication or inversion cluster, cuteSV initially creates one or more sub-clusters such that the breakpoints of all the signatures in the same sub-cluster are within 500 bp. cuteSV recognizes the major allele and minor allele sub-clusters with a heuristic rule similar to Eq.10 but sets *μ* = 1/3.
(3) Refinement of translocation clusters. Given a translocation cluster, cuteSV initially creates one or more sub-clusters such that the breakpoints of all the signatures in the same sub-cluster are within 50 bp. Furthermore, cuteSV recognizes the major allele and minor allele sub-clusters with a heuristic rule similar to Eq.10 but sets *μ* = 0.6 and *SR*_*min*_ as half of the value used for deletion/insertion clusters. This setting helps to fully consider the diverse combinations of chromosomes and orientations of translocation events to achieve higher sensitivity.

### SV calling and genotyping

For each cluster of signatures, cuteSV computes the weighted average of the positions and sizes to predict the breakpoint(s) and size of the corresponding SV and removes the predicted SVs of < 30 bp in size.

cuteSV employs a bi-allelic assumption to perform SV genotyping. If an SV site has more than one alternative alleles, each alternative allele is treated respectively. All possible bi-allelic genotypes are kept. It uses a local genomic region for a predicted SV to analyze the likelihood of various zygosity of SVs by their supporting reads, which is defined as:

12 $$ \left\{\begin{array}{c}\mathcal{L}\left(0/0\right)=\frac{1}{3}\times {\left(1-\varepsilon \right)}^{{\mathrm{SR}}_{\mathrm{Ref}}}\times {\varepsilon}^{{\mathrm{SR}}_{\mathrm{ALT}}}\\ {}\mathcal{L}\left(0/1\right)=\frac{1}{3}\times {\left(\frac{1}{2}\right)}^{{\mathrm{SR}}_{\mathrm{Ref}}+{\mathrm{SR}}_{\mathrm{ALT}}}\\ {}\mathcal{L}\left(1/1\right)=\frac{1}{3}\times {\left(1-\upvarepsilon \right)}^{{\mathrm{SR}}_{\mathrm{ALT}}}\times {\upvarepsilon}^{{\mathrm{SR}}_{\mathrm{Ref}}}\end{array}\right. $$

where *ε* is the probability that a read is being mapped to a given zygosity erroneously (default value, 0.1), assuming it is constant and independent between all observations, SR_Ref_ and SR_ALT_ are the numbers of supporting reads for reference and SV allele, respectively. After the calculation of the original likelihoods, cuteSV normalizes these values by computing the log sum of exponentials. The final genotype is determined by the zygosity with the maximum genotype likelihood. After genotyping, cuteSV produces the phred-scaled genotype likelihoods, conditional genotype quality, and phred-scaled quality score of SV in order to further quality control and higher accuracy callsets’ generation.

### Implementation of simulation

In total, 6167, 9899, and 44 deletions, insertions, and inversions were extracted from CHM1 sample callsets (nstd137 in dbVAR database). The extracted SVs are non-overlapping and meet the condition that experiment IDs are 1 or 2 and all of ACs, ANs, and AFs are higher than 0. Similarly, 3712 and 380 non-overlapping duplications and translocations were extracted from KWS1 sample callsets (nstd106 in dbVAR database), respectively. Then, the five types of SVs were respectively integrated into human reference genome (hs37d5) to build five in silico donor genomes to generate simulated datasets with VISOR simulator. For a specific type of SVs, the simulation is done by the following two steps.

1. The SVs were transformed into BED format according to the requirement of VISOR HACk. Deletions, insertions, or inversions were directly spiked into the reference to generate haplotype in silico donor genomes correspondingly. Employed translocations and duplications were preprocessed as reciprocal translocations and tandem duplications with CN = 2 before input to VISOR, respectively. It is also worth noting that translocations in same strand orientations (e.g., forward-forward and reverse-reverse reciprocal translocations) have 2 kinds of breakpoint combinations and 4 kinds of breakend combinations, and translocations in different strand orientations (e.g., forward-reverse and reverse-forward reciprocal translocations) have 4 kinds of breakpoint combinations and 4 kinds of breakend combinations. A schematic illustration is in Additional file 1: Fig. S11.
2. For a given donor genome, its in silico chromosomes were randomly selected as “homozygous” and “heterozygous” chromosomes to mimic homozygous and heterozygous SVs. For a homozygous chromosome, all the reads were simulated from the in silico chromosome with SVs. For a heterozygous chromosome, 50% of the reads were simulated from the in silico chromosome and 50% from the corresponding reference chromosome. With this rule, four PacBio-like datasets in various coverages (5×, 10×, 20×, and 30×) were produced by VISOR LASeR (using PBSIM and minimap2 with default setting). Thus, there are 20 datasets produced for the five donor genomes and the specific sets of SVs and their genotypes were used as ground truth.

The used command lines for data simulation are in the Additional file 1: Supplementary Notes.

### Implementation of long-read mapping and SV calling

PBMM2 (version 1.0.0), NGMLR (version 0.2.3), and Minimap2 (version 2.17) were employed to implement the read alignment of the benchmarking datasets. The parameter “--preset” of long-read aligners was tuned for various types of sequencing data. Samtools (version 1.9) was employed for read extraction, sorted BAM generation, and sequencing data down-sampling.

For Sniffles (version 1.0.11), the configuration “-l 30 -s 1/2/3/4 --genotype” was used for simulated datasets, “-l 30 -s 2/3/4/4/5/10 --genotype” was used for PacBio CLR datasets, “-l 30 -s 1/2/3 --genotype” for was used for PacBio CCS reads, and “-l 30 -s 2/3/4/10 --genotype” was used for ONT PromethION reads.

For PBSV (version 2.2.0), default settings were used for simulated, PacBio CLR and ONT PromethION data, and “--preset CCS” was used for PacBio CCS data.

For SVIM (version 0.4.3), the configuration “--min_sv_size 30” was employed for all datasets in this study.

For cuteSV (version 1.0.6), the configuration “-l 30 -s 1/2/3/4 --genotype” was used for simulations, “-l 30 --genotype” was used for PacBio CLR (“-s 2/3/4/4/5/10”) and ONT PromethION reads (“-s 2/3/4/10”), and “-l 30 -s 1/2/3 --genotype --max_cluster_bias_INS 200 --diff_ratio_merging_INS 0.65 --diff_ratio_filtering_INS 0.65 --diff_ratio_filtering_DEL0.35” was used for PacBio CCS reads.

The used command lines for the tools are in the Additional file 1: Supplementary Notes.

### Evaluation of SV callsets

The SV calls from simulated data were assessed based on the ground truths in the following approach. For deletions, insertions, duplications, and inversions, a prediction is determined as a true-positive (TP) when meeting the following conditions:

13 $$ \left\{\begin{array}{c}\max \left({\mathrm{comp}}_{\mathrm{s}}-1\mathrm{kbp},{\mathrm{base}}_s\right)\le \min \left({\mathrm{comp}}_{\mathrm{e}}+1\mathrm{kbp},{\mathrm{base}}_{\mathrm{e}}\right)\ \\ {}\min \left({\mathrm{comp}}_L,{\mathrm{base}}_{\mathrm{L}}\right)/\max \left({\mathrm{comp}}_L,{\mathrm{base}}_{\mathrm{L}}\right)\ge 0.7\\ {}{\mathrm{comp}}_t={\mathrm{base}}_t\end{array}\right. $$

where comp_s_, comp_e_, comp_*L*_, and comp_*t*_ indicate start coordinate, stop coordinate, size, and SV class of a prediction, and base_s_, base_e_, base_*L*_, and base_*t*_ are starting coordinate, end coordinate, size, and SV class of a SV recorded in the ground truth, respectively. In terms of translocations, a prediction is determined as a TP call at breakpoint level if it meets the following conditions:

14 $$ \left\{\begin{array}{c}\left|{\mathrm{comp}}_{\mathrm{BK}1}-{\mathrm{base}}_{\mathrm{BK}1}\right|\le 1\mathrm{kbp}\ \\ {}\left|{\mathrm{comp}}_{\mathrm{BK}2}-{\mathrm{base}}_{\mathrm{BK}2}\right|\le 1\mathrm{kbp}\ \\ {}{\mathrm{comp}}_{\mathrm{Chr}1}={\mathrm{base}}_{\mathrm{Chr}1}\\ {}{\mathrm{comp}}_{\mathrm{Chr}2}={\mathrm{base}}_{\mathrm{Chr}2}\end{array}\right. $$

where BK1, BK2, Chr1, and Chr2 are the combination of breakpoints and chromosomes of a call on the prediction and base, respectively. Moreover, a prediction is determined as a TP call at breakend level if meeting the following conditions:

15 $$ \left\{\begin{array}{c}\left|{\mathrm{comp}}_{\mathrm{BK}1}-{\mathrm{base}}_{\mathrm{BK}1}\right|\le 1\mathrm{kbp}\ \\ {}\left|{\mathrm{comp}}_{\mathrm{BK}2}-{\mathrm{base}}_{\mathrm{BK}2}\right|\le 1\mathrm{kbp}\ \\ {}{\mathrm{comp}}_{\mathrm{Chr}1}={\mathrm{base}}_{\mathrm{Chr}1}\\ {}{\mathrm{comp}}_{\mathrm{Chr}2}={\mathrm{base}}_{\mathrm{Chr}2}\\ {}{\mathrm{comp}}_{\mathrm{cnt}}={\mathrm{base}}_{\mathrm{cnt}}\end{array}\right. $$

where comp_cnt_ and base_cnt_ are the connectivity of breakends on the prediction and base, respectively. A prediction is determined as a false positive (FP) if it does not satisfy Eqs.13–15. A ground truth SV is assigned as a false negative (FN) if there is no SV call satisfies Eq.13–15 with it. With the above definition, precision (or the ratio of TPs to total calls in predictions) is defined as

16 $$ \mathrm{Precision}=\frac{\mathrm{TPs}}{\mathrm{TPs}+\mathrm{FPs}} $$

Similarly, recall (or the ratio of TPs to total calls in the truth set) is defined as:

17 $$ \mathrm{Recall}=\frac{\mathrm{TPs}}{\mathrm{TPs}+\mathrm{FNs}} $$

F1 score is defined as

18 $$ \mathrm{F}1=\frac{2\times \mathrm{Precision}\times \mathrm{Recall}}{\mathrm{Precision}+\mathrm{Recall}} $$

Moreover, when considering the zygosity, if the prediction satisfies Eqs.13–15 and its genotype is same as corresponding base call, it will be a TP-GT call. Hence, we reuse Eq.16–18 to calculate the statistics under genotyping.

The evaluation of the HG002 human sample was done by Truvari (version 1.2), and the high confidence insertion and deletion callsets (available at https://ftp-trace.ncbi.nlm.nih.gov/giab/ftp/data/AshkenazimTrio/analysis/NIST_SVs_Integration_v0.6/HG002_SVs_Tier1_v0.6.vcf.gz) and high confidence regions (https://ftp-trace.ncbi.nlm.nih.gov/giab/ftp/data/AshkenazimTrio/analysis/NIST_SVs_Integration_v0.6/HG002_SVs_Tier1_v0.6.bed) published by GAIB consortium were used as ground truth. Before evaluation, we preprocessed the SV calls of each tool. For Sniffles, we discarded inversions and translocations, and transform duplications to insertions. For SVIM, we transformed duplications to insertions as well, deleted SV calls with a quality score of less than 5, and kept the supporting reads consistent with Sniffles and cuteSV in each corresponding dataset. For PBSV and cuteSV, we only selected insertions and deletions for evaluation. Then, BGZIP and TABIX were employed to compress and index the processed VCF files. In addition, only SV calls between 50 bp and 10 kbp being within the GIAB high confidence regions were considered for evaluation.

To benchmark the NA19240 human sample, we select SV calls in each tool with 50 bp to 10 k bp in size and with 5 supporting reads at least, besides, we discard low quality (QUAL below 5) calls in cuteSV and SVIM. We used Eq.13 to assess every call against the deletion, insertion (duplication regarded as a subset of insertion), and inversion in the callsets generated from the study [48], and Eqs. 16 to 18 were employed to summarize the performance of SV calling.

For the evaluation of the Ashkenazi trio, we used the homozygous SV calls in parents (i.e., HG003 and HG004) to measure the recall rate via Eq.13–15 as follows:

19 $$ \mathrm{Recall}\_\mathrm{trio}=\frac{\sum \mathrm{SVs}\ \mathrm{in}\ \mathrm{the}\ \mathrm{offspring}}{\sum \mathrm{homozygous}\ \mathrm{SVs}\ \mathrm{in}\ \mathrm{parents}} $$

We also used all parental SV calls to assess MDR, i.e., the percentage of SV calls of the son (HG002) that cannot be detected in its parents:

20 $$ \mathrm{MDR}=\frac{\sum \mathrm{offsprin}{\mathrm{g}}^{\prime}\mathrm{s}\ \mathrm{SVs}\ \mathrm{not}\ \mathrm{reidentified}\ \mathrm{in}\ \mathrm{parents}}{\sum \mathrm{offsprin}{\mathrm{g}}^{\prime}\mathrm{s}\ \mathrm{SVs}} $$

To evaluate the computational performance of the SV callers with various numbers of CPU threads w/o genotyping, runtime and memory footprint were assessed by using the “/usr/bin/time -v” command of the Linux Operating System. In the output results of the command, “Elapsed (wall clock) time” and “Maximum resident set size” indicate the elapsed runtime and memory consumption, respectively. It is worth noting that we used the sum of the wall clock time of both steps as the final elapsed runtime, because SV calling performed by PBSV involves two steps (i.e., discover and call). Meanwhile, the memory footprint depends on the maximum memory usage of the two runs.

Refer to Additional file 1: Supplementary Notes for the used command lines of benchmark.

## Supplementary information

**Additional file 1: Figs. S1-S11.** Supplemental Figures. **Tables S1-S20.** Supplemental Tables. **Supplementary Notes.** Command lines for evaluation.

**Additional file 2.** Review history.

## Data Availability

The source code of cuteSV and the scripts of data simulations and benchmarking are available at https://github.com/tjiangHIT/cuteSV [50] under MIT open source license. The cuteSV release used in this article is deposited on Zenodo with doi:10.5281/zenodo.3911487 [51]. The hs37d5 human reference genome is downloaded from IGSR [52]: ftp://ftp.1000genomes.ebi.ac.uk/vol1/ftp/technical/reference/phase2_reference_assembly_sequence/hs37d5.fa.gz. The Ashkenazim Trio (including HG002, HG003, and HG004) raw sequence data, alignments, and corresponding ground truth sets for evaluation are available at https://ftp.ncbi.nih.gov/giab/ftp/data/AshkenazimTrio/. The NA19240 sequencing dataset is available at ftp://ftp.1000genomes.ebi.ac.uk/vol1/ftp/data_collections/hgsv_sv_discovery/working/20160905_smithm_pacbio_aligns/NA19240_bwamem_GRCh38DH_YRI_20160905_pacbio.bam, and its ground truth sets is downloaded from NCBI dbVAR [53]: ftp://ftp.ncbi.nlm.nih.gov/pub/dbVar/data/Homo_sapiens/by_study/vcf/nstd152.GRCh37.variant_call.vcf.gz. The CHM1 human sample SV callsets used for generation of simulated deletions, insertions, and inversions are available at https://ftp.ncbi.nlm.nih.gov/pub/dbVar/data/Homo_sapiens/by_study/vcf/nstd137.GRCh37.variant_call.vcf.gz. The KWS1 human sample SV callsets used for generation of simulated duplications and translocations are available at https://ftp.ncbi.nlm.nih.gov/pub/dbVar/data/Homo_sapiens/by_study/vcf/nstd106.GRCh37.variant_call.vcf.gz.
