# Supplementary material for: Long-read-based human genomic structural variation detection with cuteSV
Source: Genome Biol. 2020 Aug 3;21:189. doi: 10.1186/s13059-020-02107-y (PMC7477834; doi:10.1186/s13059-020-02107-y)
Supplement: Supplementary file 1 — Additional file 1: Figs. S1-S11. Supplemental Figures. Tables S1-S20. Supplemental Tables. Supplementary Notes. Command lines for evaluation. [file 13059_2020_2107_MOESM1_ESM.docx]

Long-read-based human genomic structural variation detection with cuteSV

Tao Jiang^1,#^, Yongzhuang Liu^1,#^, Yue Jiang^2^, Junyi Li^3^, Yan Gao^1^, Zhe Cui^1^, Yadong Liu^1^, Bo Liu^1,*^ and Yadong Wang^1,*^

^1^ Center for Bioinformatics, School of Computer Science and Technology, Harbin Institute of Technology, Harbin, Heilongjiang 150001, China

^2^ Nebula Genomics, Harbin, Heilongjiang 150030, China

^3^ School of Computer Science and Technology, Harbin Institute of Technology (Shenzhen), Shenzhen, Guangdong 518055, China

^#^ Tao Jiang and Yongzhuang Liu contributed equally to this work.

^*^ Correspondence: [bo.liu@hit.edu.cn](mailto:bo.liu@hit.edu.cn) and [ydwang@hit.edu.cn](mailto:ydwang@hit.edu.cn)

**Content**

[Fig. S1. The Venn diagram of various SV types identified by cuteSV on the HG002 human sample datasets with multiple long-read sequencing technologies. 3](#_Toc44492245)

[Fig. S2. An example of an insertion only being detected with ONT PromethION data 5](#_Toc44492246)

[Fig. S3. Comparison of the callsets of the PacBio CLR dataset produced with various long-read aligners. 7](#_Toc44492247)

[Fig. S4. The results of cuteSV with various configurations on the parameters *--min_support* and *–min_size* 8](#_Toc44492248)

[Fig. S5. The results of the SV callers on the PacBio CLR dataset from NA19240 sample 9](#_Toc44492249)

[Fig. S6. An example of fragile read alignments around insertion events 10](#_Toc44492250)

[Fig. S7. Two examples of the read alignments around multi-allelic SVs 13](#_Toc44492251)

[Fig. S8. An example of a false-negative deletion 15](#_Toc44492252)

[Fig. S9. An example of a false-negative insertion 17](#_Toc44492253)

[Fig. S10. An example of the alignments of a “copy-and-paste” insertion with duplication 19](#_Toc44492254)

[Fig. S11. The illustration of reciprocal translocation at breakpoint and breakend level 21](#_Toc44492255)

[Table S1. Benchmark results of simulated deletions 22](#_Toc44492256)

[Table S2. Benchmark results of simulated insertions 23](#_Toc44492257)

[Table S3. Benchmark results of simulated duplications 24](#_Toc44492258)

[Table S4. Benchmark results of simulated inversions 25](#_Toc44492259)

[Table S5. Benchmark results of simulated reciprocal translocations (breakpoint level) 26](#_Toc44492260)

[Table S6. Benchmark results of simulated reciprocal translocations (breakend level) 27](#_Toc44492261)

[Table S7. Genotype confusion matrices of callsets with different SV callers on 30× simulated datasets 28](#_Toc44492262)

[Table S8. The distinction of accuracy between cigar-only calls and split-alignment-only calls on 30× simulated datasets 29](#_Toc44492263)

[Table S9. Benchmark results on the 69× HG002 PacBio CLR datasets 30](#_Toc44492264)

[Table S10. Benchmark results on the 28× HG002 PacBio CCS datasets 31](#_Toc44492265)

[Table S11. Benchmark results on Ashkenazi human trio 32](#_Toc44492266)

[Table S12. Benchmark results on the 47× HG002 ONT PromethION datasets 33](#_Toc44492267)

[Table S13. Benchmark results of cuteSV callsets with various sequencing technologies datasets on HG002 34](#_Toc44492268)

[Table S14. Benchmark results of callsets from different tools on various sequencing technologies 35](#_Toc44492269)

[Table S15. The results of runtime and memory footprint of various sequencing technologies datasets on HG002 36](#_Toc44492270)

[Table S16. The number of false negative calls of HG002 sample under different methods 37](#_Toc44492271)

[Table S17. The number of false positive calls of HG002 sample under different methods 38](#_Toc44492272)

[Table S18. The precision and recall of HG002 sample for various sequencing coverages and the configurations of *--min_support* parameter 39](#_Toc44492273)

[Table S19. Benchmark results on the 40× NA19240 PacBio CLR dataset 40](#_Toc44492274)

[Table S20. Nomenclature 41](#_Toc44492275)

[Supplementary Notes 42](#_Toc44492276)


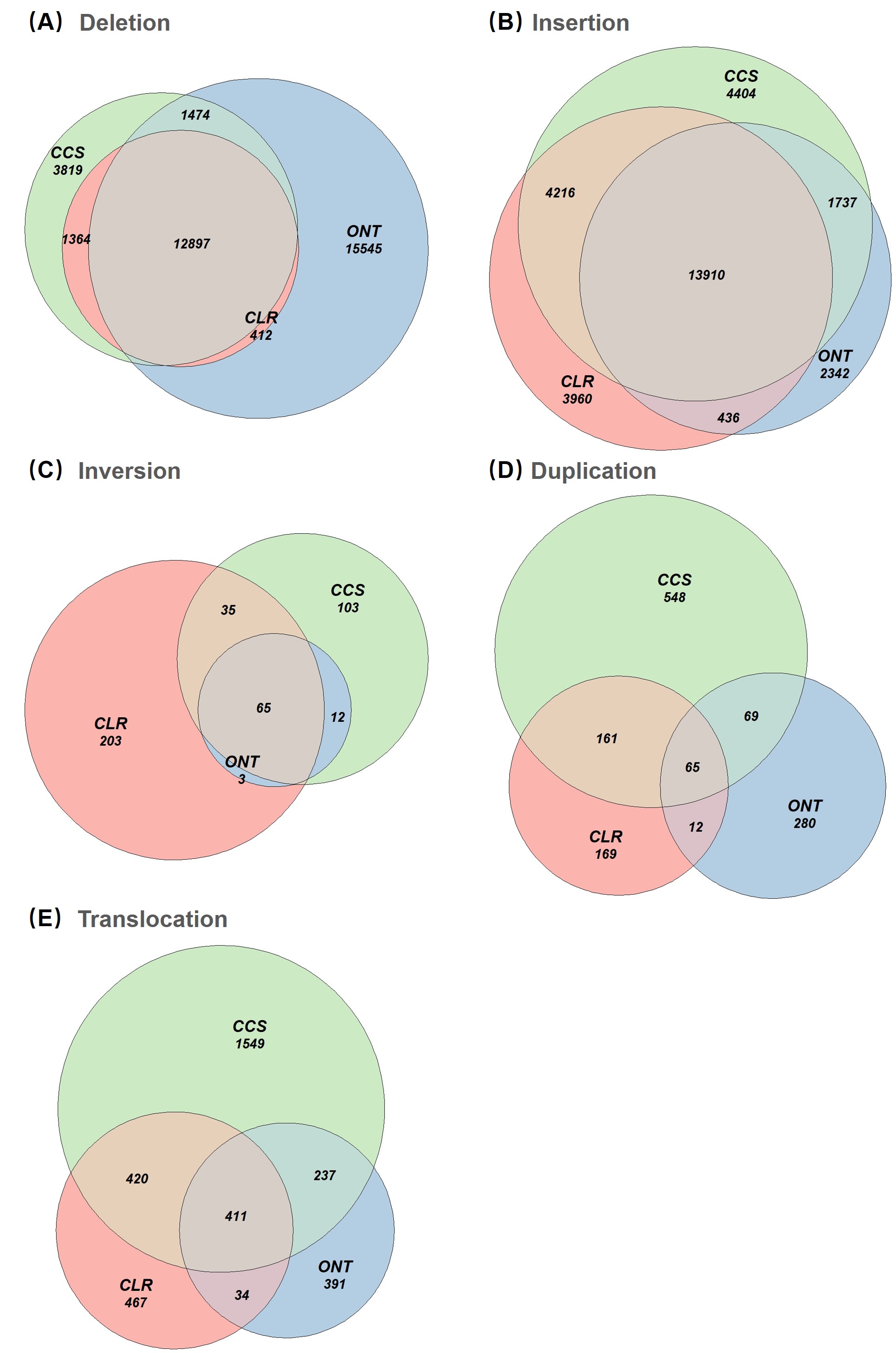


**Fig. S1. The Venn diagram of various SV types identified by cuteSV on the HG002 human sample datasets with multiple long-read sequencing technologies.**

(**A**) The Venn diagram of deletion. (**B**) The Venn diagram of insertion. (**C**) The Venn diagram of inversion. (**D**) The Venn diagram of duplication. (**A**) The Venn diagram of translocation. CLR, CCS and ONT indicate PacBio CLR, CCS and ONT PromethION sequencing data.


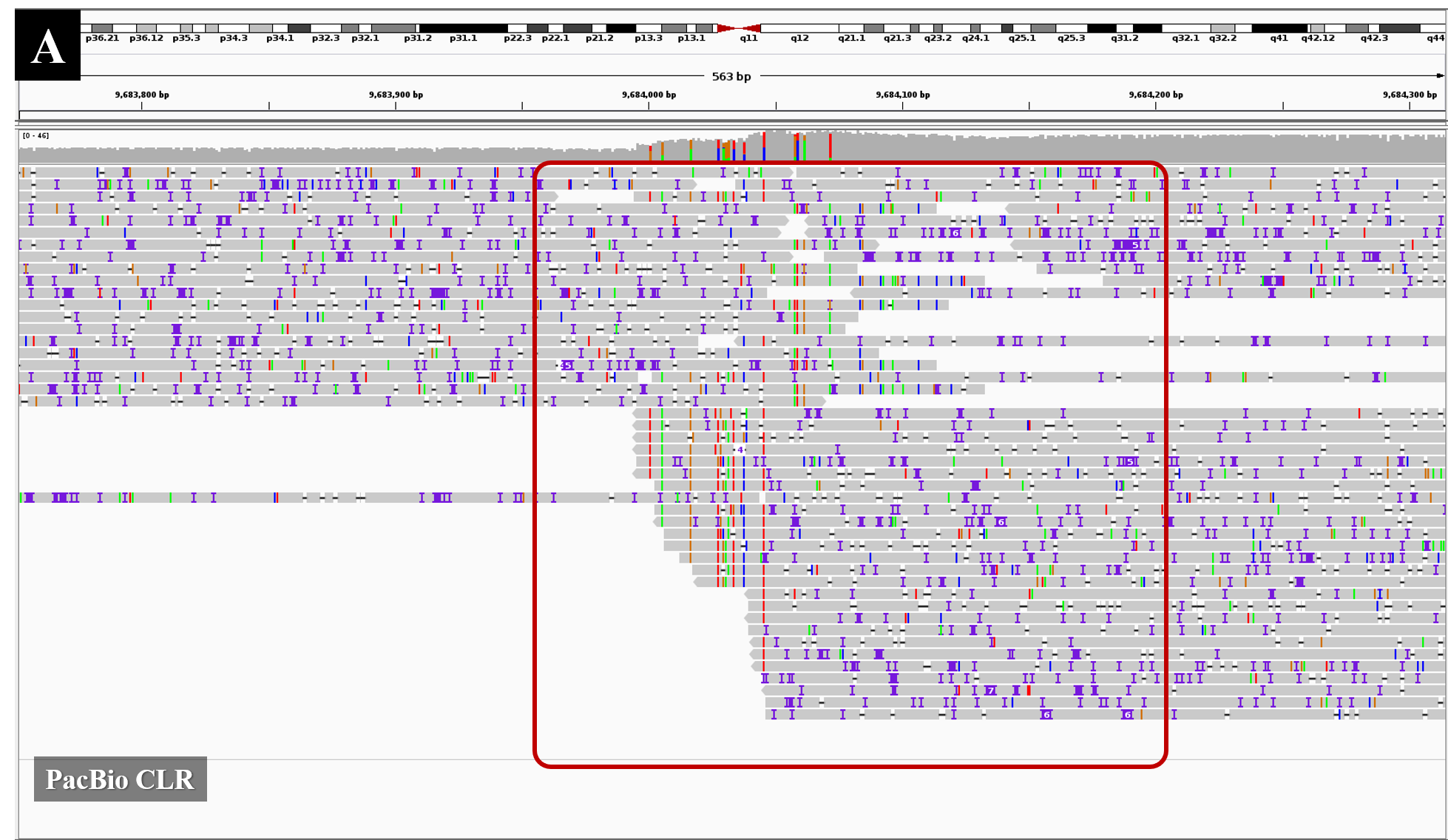


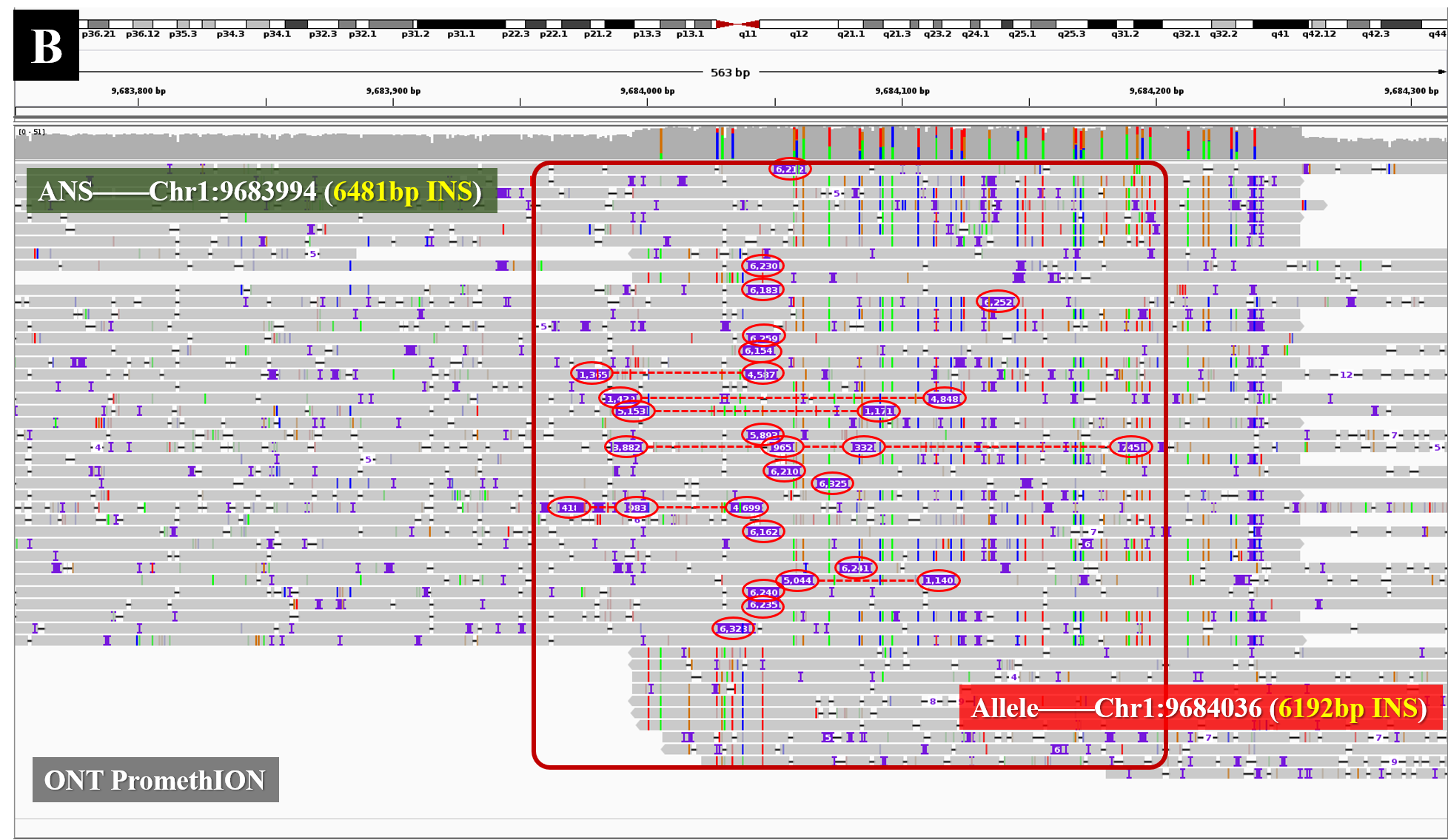


**Fig. S2. An example of an insertion only being detected with ONT PromethION data**

According to the ground truth, there is a 6481 bp insertion at chr1:9683994. (A) The Integrated Genomics Viewer (IGV) snapshot of the PacBio CLR read alignments at chr1:9683700-9684300 indicates that nearly 50% of the reads mapped to this region are about 5000 bp long and few of them are longer than 10 kbp. Due to the limited read length, it is difficult to produce alignments with such a large insertion, so none of the PacBio reads have an obvious insertion signature and cuteSV (as well as other SV callers) failed for this SV event. (B) The IGV snapshot of the ONT read alignments indicates that the minimum and average lengths of the reads mapped to this region are 18kbp and 58kbp, respectively. With superior read lengths, 20 reads were aligned with >6000 bp insertion in their CIGARs and they are very useful SV signatures. cuteSV captured these signatures successfully and made a 6192 bp insertion at chr1:9684036, which highly coincides with the ground truth.


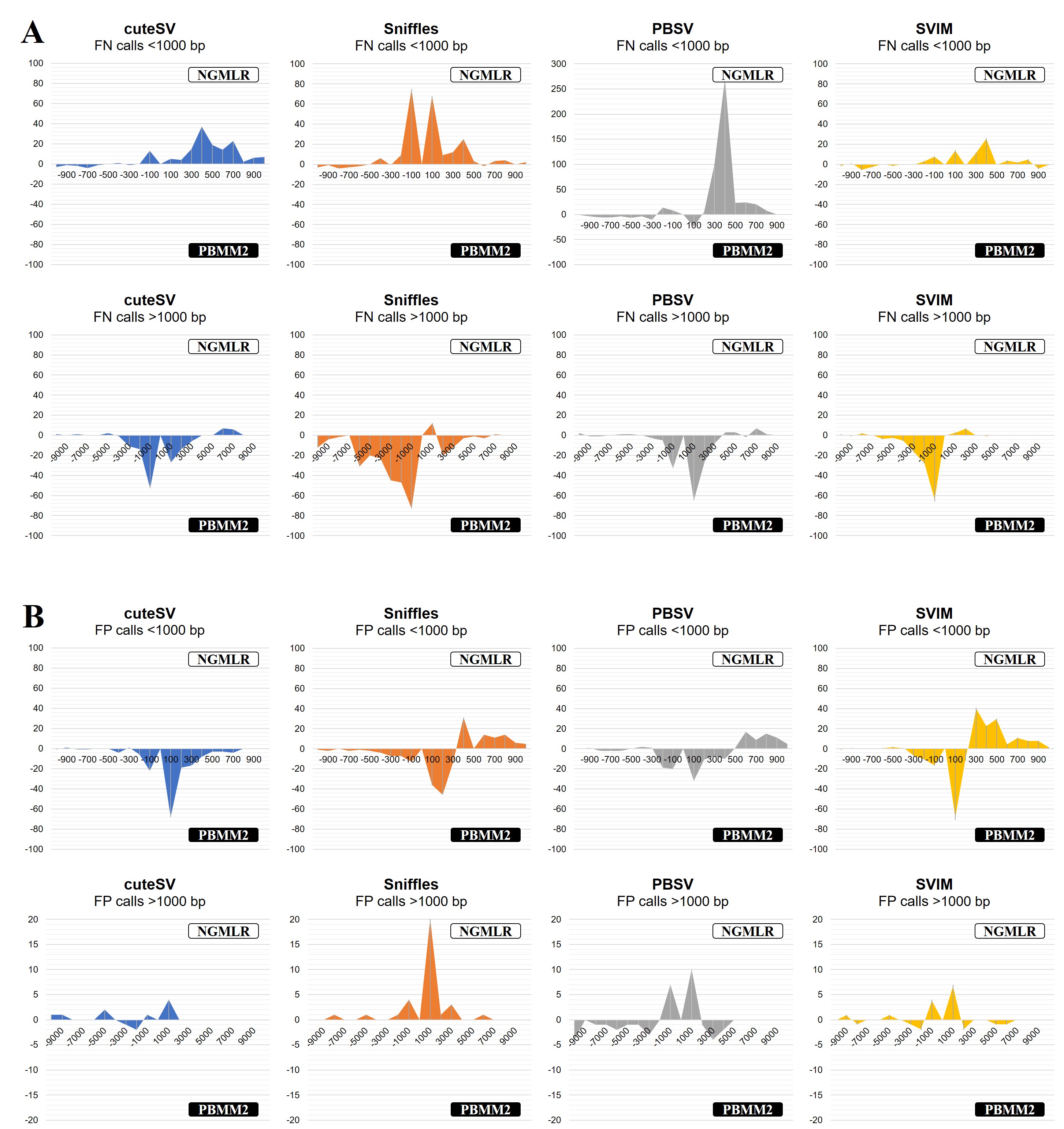


**Fig. S3. Comparison of the callsets of the PacBio CLR dataset produced with various long-read aligners.**

(**A**) The difference between the false-negative (FN) calls in various SV sizes with NGMLR and PBMM2. (**B**) The difference between the false-positive (FP) calls in various SV sizes with NGMLR and PBMM2. It is worth noting that a positive value indicates the occurrence of more corresponding calls (e.g., FN and FP) when using NGMLR, whereas a negative value means the generation of more corresponding calls with PBMM2. The smaller the overall difference, the better the compatibility with the long-read aligners.


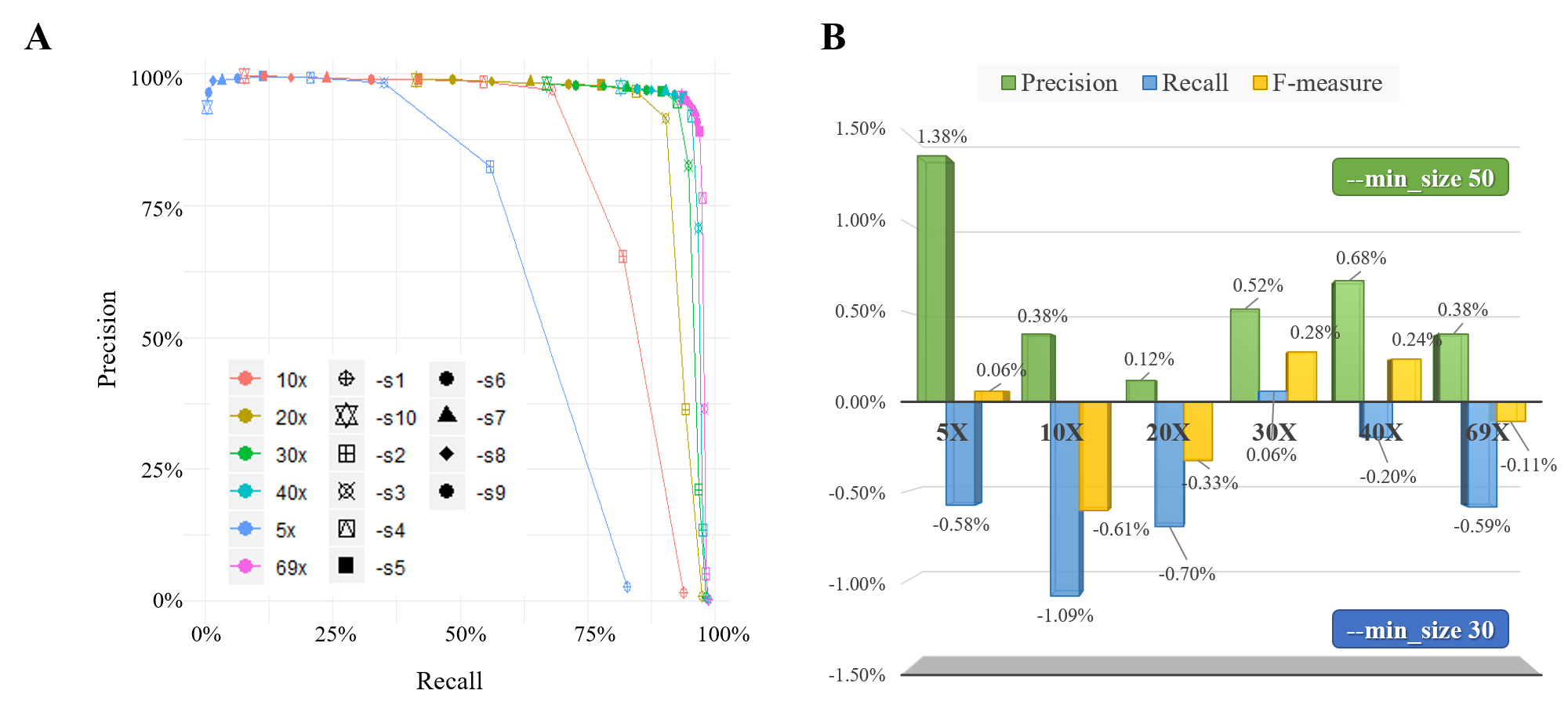


**Fig. S4. The results of cuteSV with various configurations on the parameters *--min_support* and *–min_size***

(A) The precision and recall for various sequencing coverages and the configurations of *--min_support* parameter. (B) The differences in precision, recall, and F-measure between two different configurations of *–min_size* parameter (*–min_size* = 30 and *–min_size* = 50). It is worth noting that the positive values indicate that cuteSV achieved higher statistics with *–min_size* = 50, and the negative values indicate higher statistics with *–min_size* = 30.


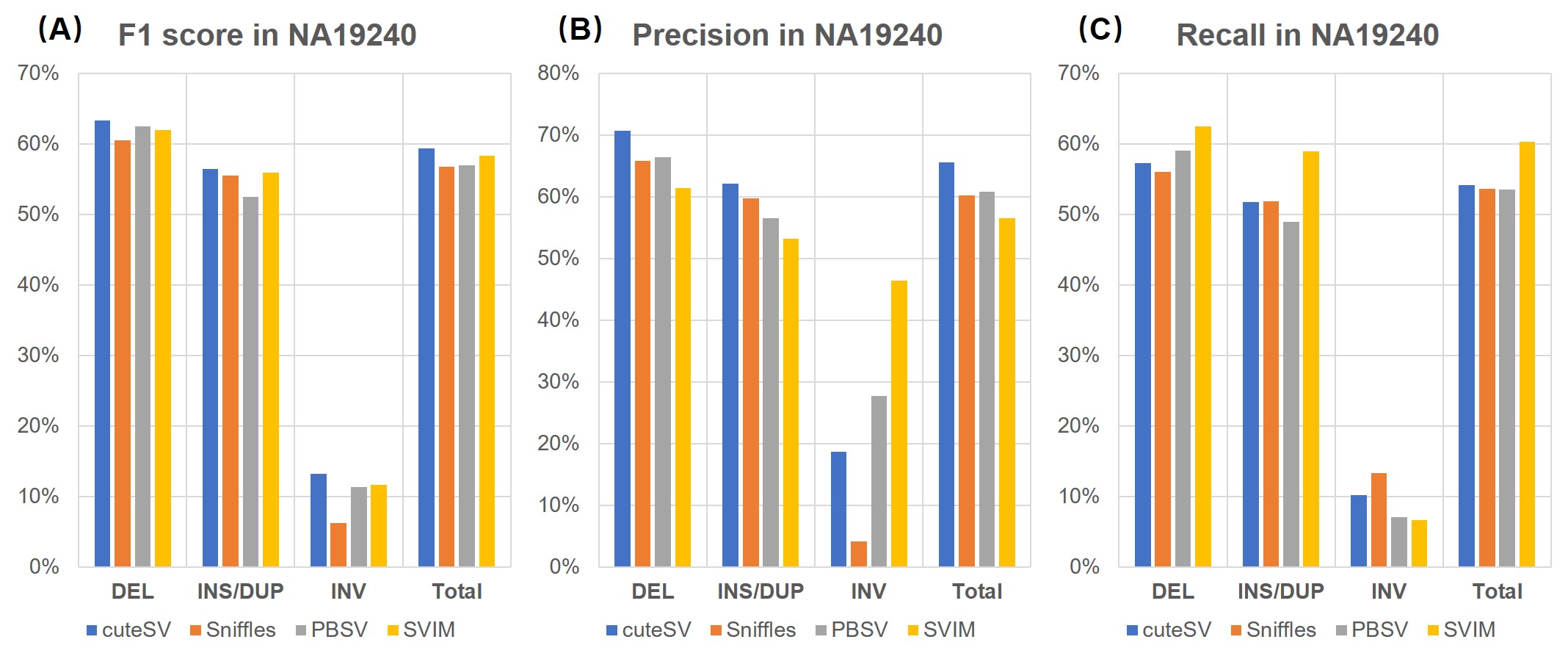


**Fig. S5. The results of the SV callers on the PacBio CLR dataset from NA19240 sample**

The subplots depict the (**A**) F1 score, (**B**) precision and (**C**) recalls of the benchmarked SV callers for various types of SVs.


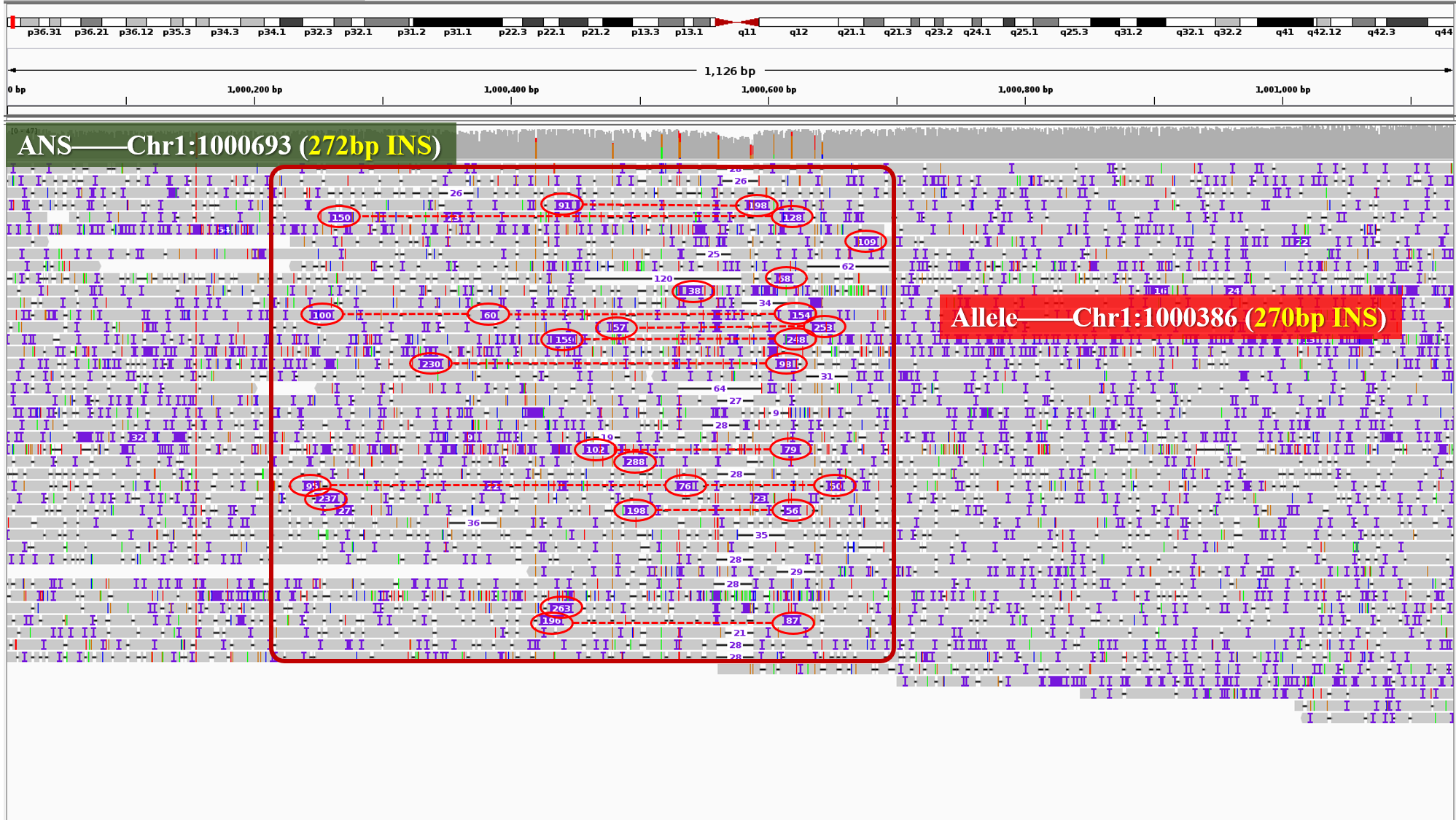


**Fig. S6. An example of fragile read alignments around insertion events**

According to the ground truth, there is a 272 bp insertion whose breakpoint is at chr1:1000693. The IGV snapshot of the PacBio CLR read alignments around this event is shown in the figure. It can be seen that many of the reads mapped to this region have insertion signatures. However, most of them have two nearby large insertions in their CIGARs instead of a large insertion (indicated by the red circles and dashed lines in the figure). In this case, cuteSV recognized 28 insertion signatures longer than 30 bp in length, and 22 of them satisfied the signature merging rule. cuteSV merged the split signatures to recover the signatures of the real larger insertion, which helps to make an insertion call (270 bp insertion at chr1:1000386) coinciding with the ground truth.


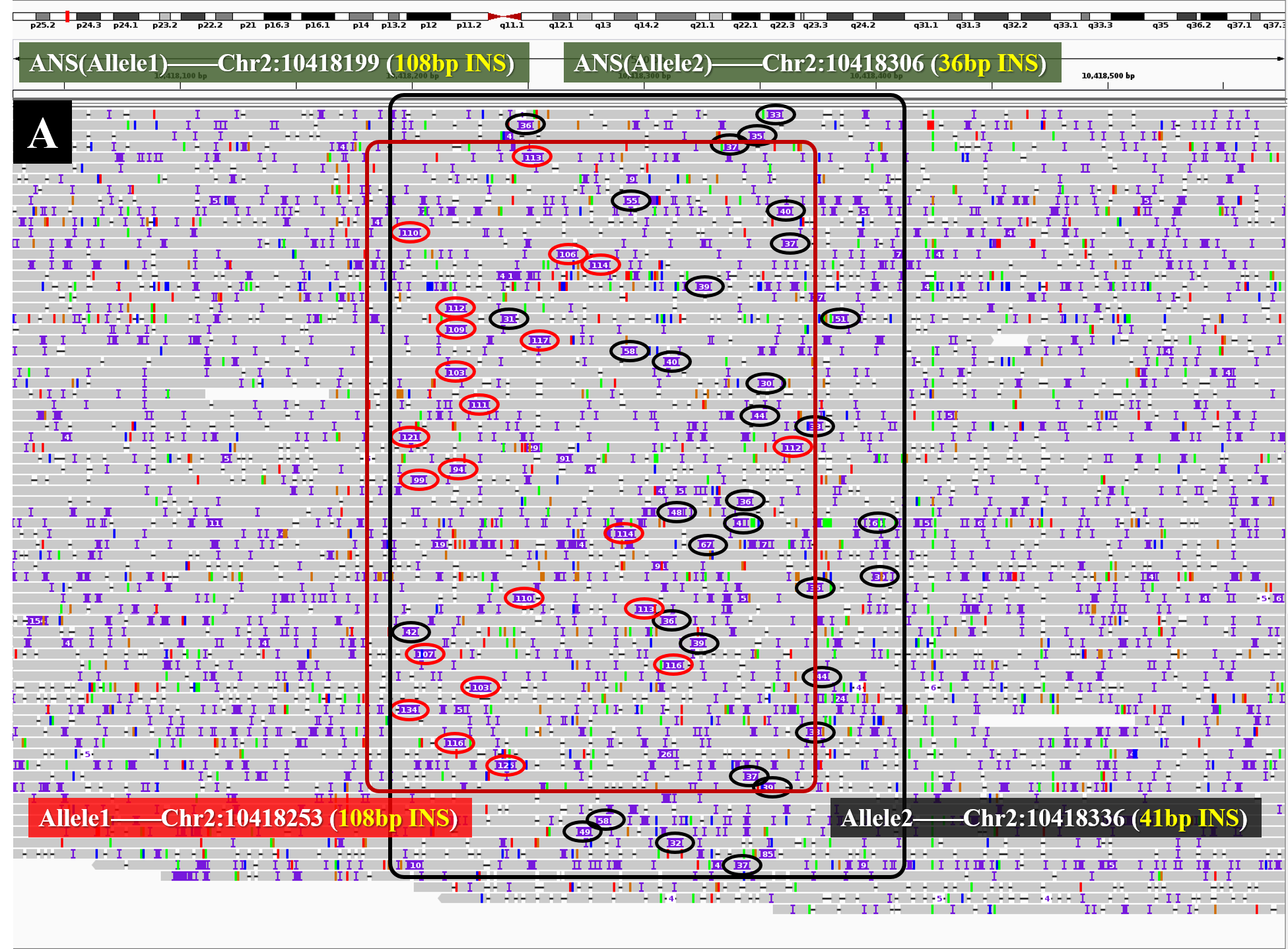


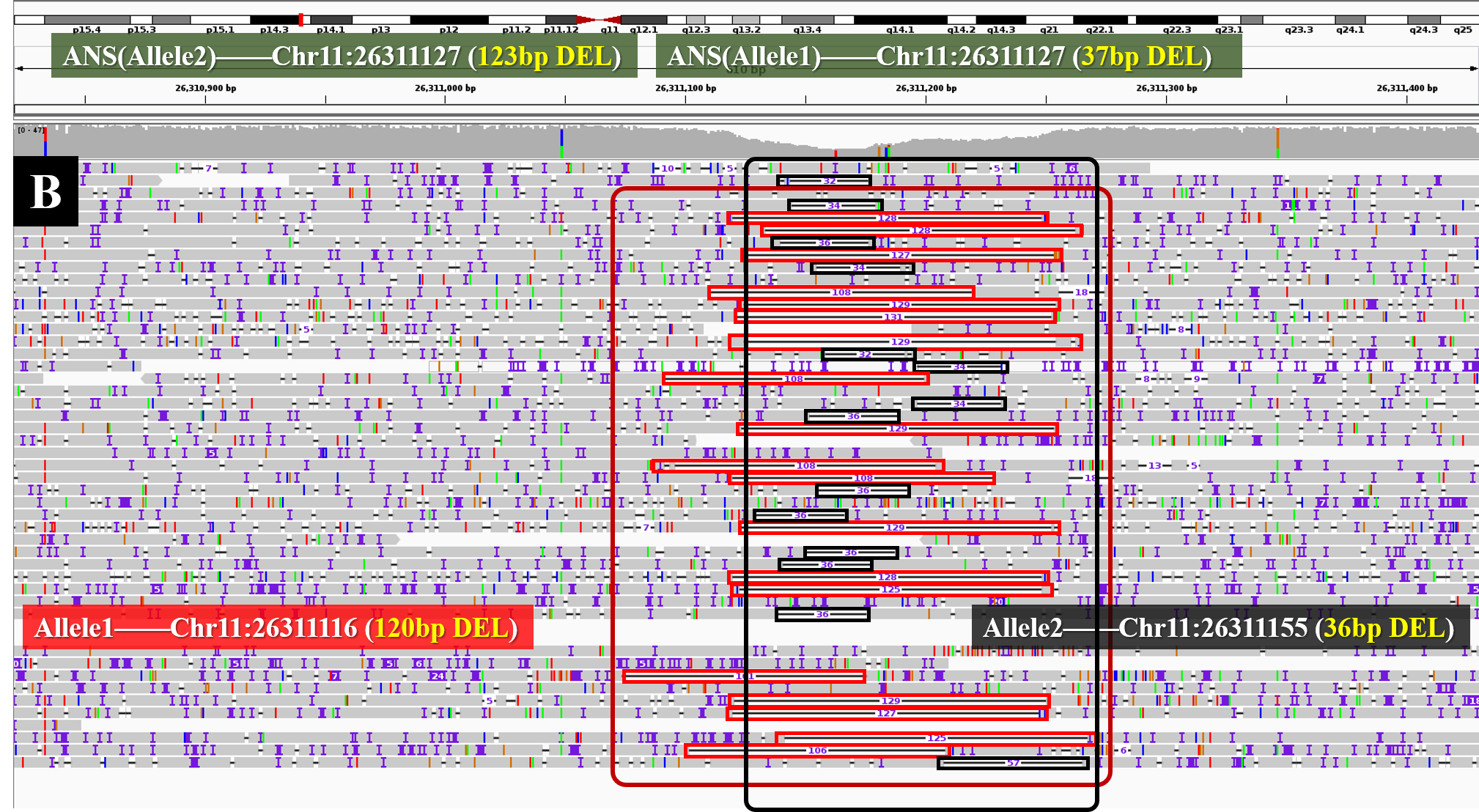


**Fig. S7. Two examples of the read alignments around multi-allelic SVs**

(A) According to the ground truth, there are two different insertions, i.e., a 108 bp insertion at chr2:10418199 and a 36 bp insertion at chr2:10418306. The IGV snapshot of the PacBio CLR read alignments around these events is shown in the figure. Many reads can be found mapped to this region with insertions of about 100 bp (marked with the red circle) and 40 bp (marked with the black circle) in their CIGARs. In this case, cuteSV called two different SVs, i.e., a 108 bp insertion at chr2:10418253 and a 41 bp insertion at chr2:10418336. (B) There are two ground truth deletions, i.e., a 123 bp deletion at chr11:26311127 and a 37 bp deletion at chr11:26311127. cuteSV recognized one set of deletion signatures around 120 bp (marked with the red box) and one set of deletion signatures around 36 bp (marked with the black box). As a result, cuteSV made two deletion calls, i.e., a 120 bp at chr11:26311116 and a 36 bp at chr11:26311155. All these SV calls coincide with the ground truth.


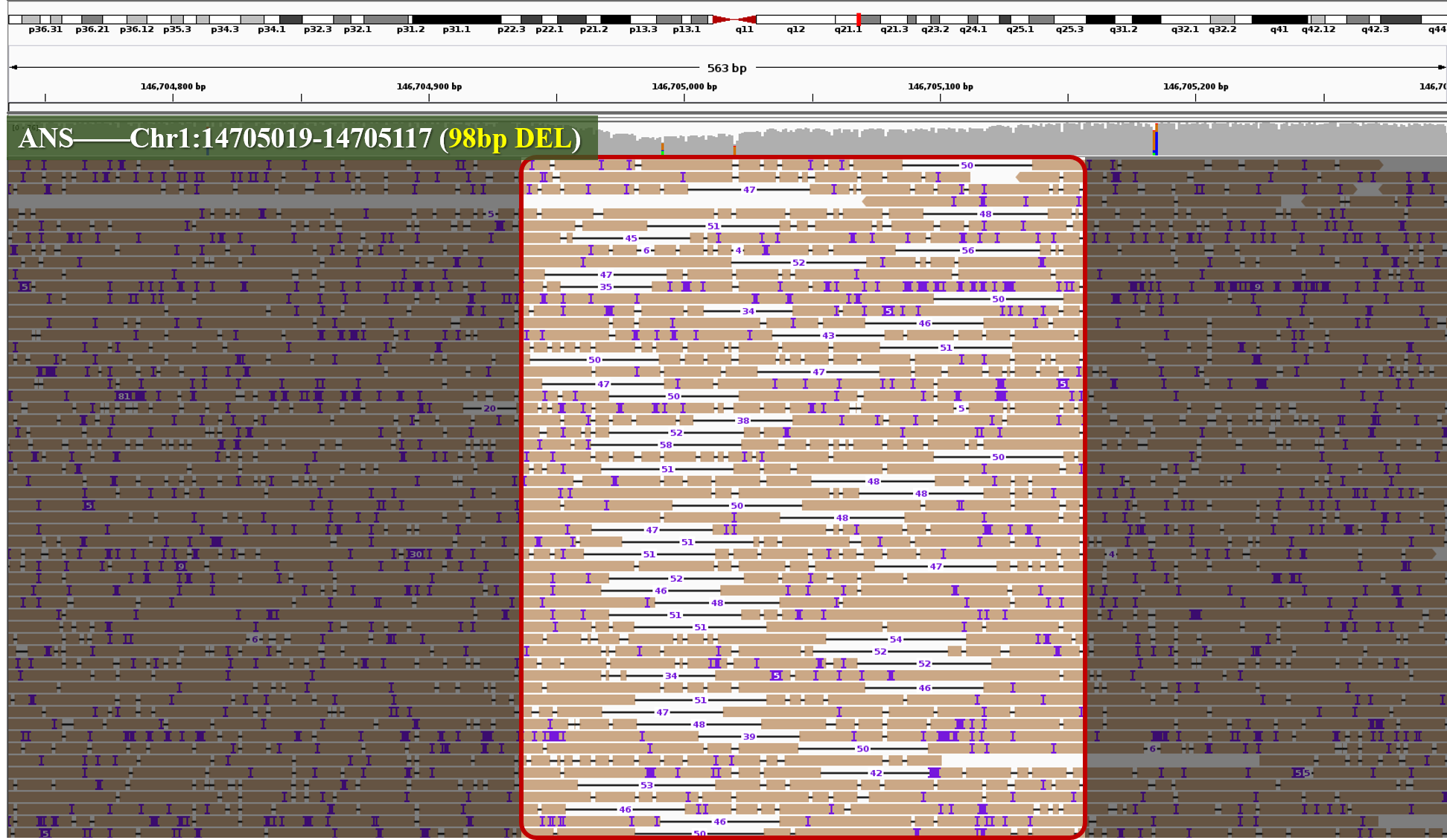


**Fig. S8. An example of a false-negative deletion**

According to the ground truth, there is a 98 bp deletion at chr1:14705019-14705117. The IGV snapshot of the PacBio CLR read alignments around this event is shown in the figure. In this region, there are only reads with 30 to 50 bp deletion in their CIGARs. Hence, cuteSV made a deletion call of about 50 bp. Meanwhile, none of the other benchmarked SV callers detected the 98bp deletion.


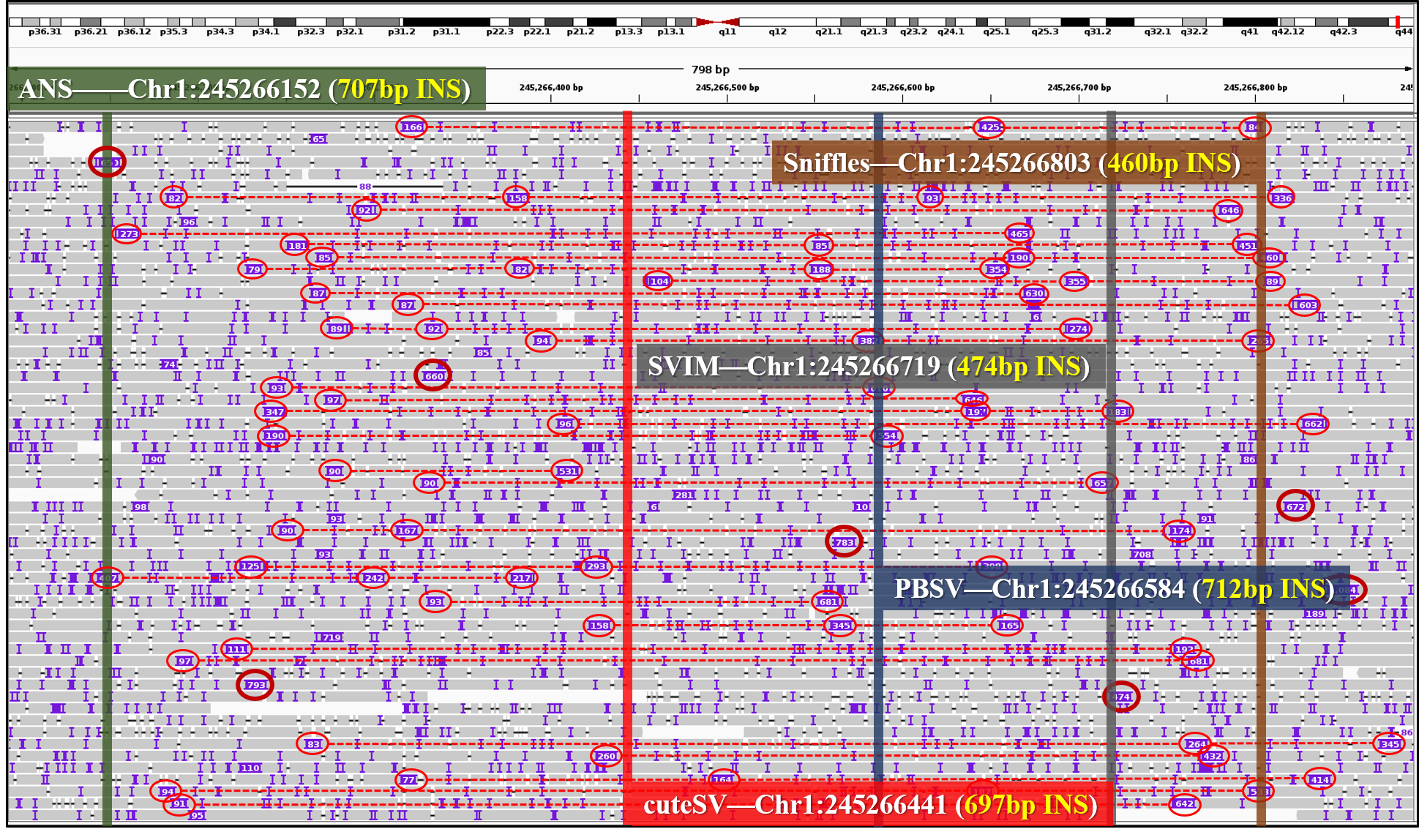


**Fig. S9. An example of a false-negative insertion**

According to the ground truth, there is a 707 bp insertion at chr1:245266152. The IGV snapshot of the PacBio CLR read alignments around this event is shown in the figure. In this region, cuteSV recognized a lot of linked fragile insertion signatures (marked by red dashed lines in the figure) and used them to make a 697 bp insertion at chr1:245266441. However, the breakpoint of this call is 289 bp away from the breakpoint of a nearby ground truth call, so Truvari determined that the ground truth call is a false negative one. Moreover, PBSV made a 712 bp insertion at chr1:245266584, which is 432 bp away from the ground truth. For Sniffles and SVIM, their calls are even more different from the ground truth.

**
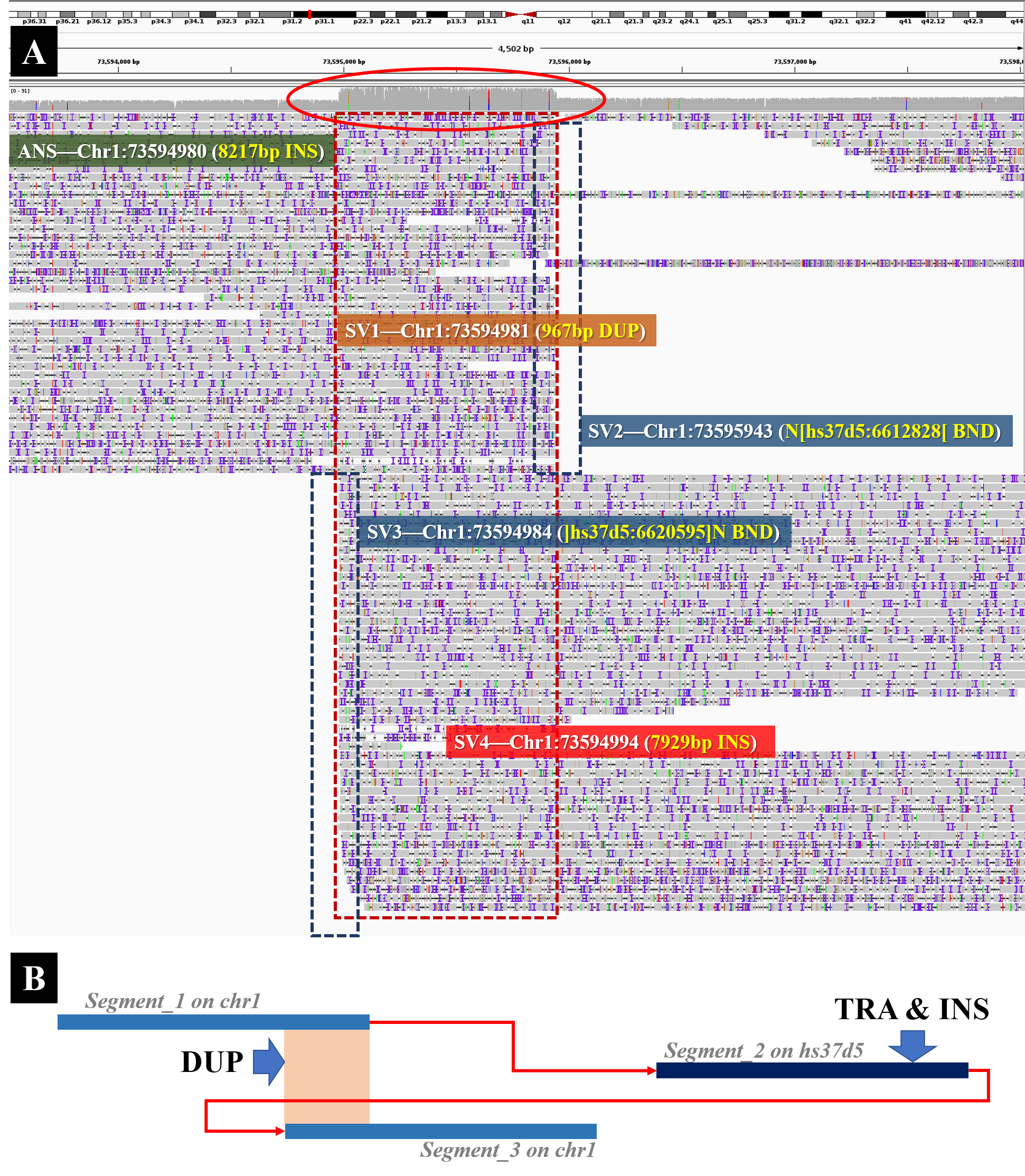
**

**Fig. S10. An example of the alignments of a “copy-and-paste” insertion with duplication**

(A) The snapshot of IGV for the alignments of ONT PromethION reads around a ground truth insertion (8217 bp insertion, breakpoint at chr1:73594980). In this local region, cuteSV recognized the signatures of translocation (marked by the deep blue dashed box) and made two BND calls, i.e., chr1:73595943(N[hs37d5:6612828) and chr1:73594984(]hs37d5:6620595]N). Moreover, cuteSV also recognized the signatures of duplication (marked by the red dotted box) and made a duplication call (duplication at chr1:73594981). (B) A schematic illustration of the complex SV event. This event can be seen as a local arrangement of three genomic segments (marked as segment_1, segment_2 and segment_3 in the figure). It is observed that segment_2 is a segment which can be aligned to a decoy sequence of the reference (hs37d5).This case can be considered as a copy-and-paste event, i.e., segment_2 is copied from the decoy sequence and pasted in between segment_1 and segment_3. In this situation, cuteSV skips the translocated segment (segment_2) and sees it as an insertion (7929 bp insertion at chr1:73594994) between segment_1 and segment_3.


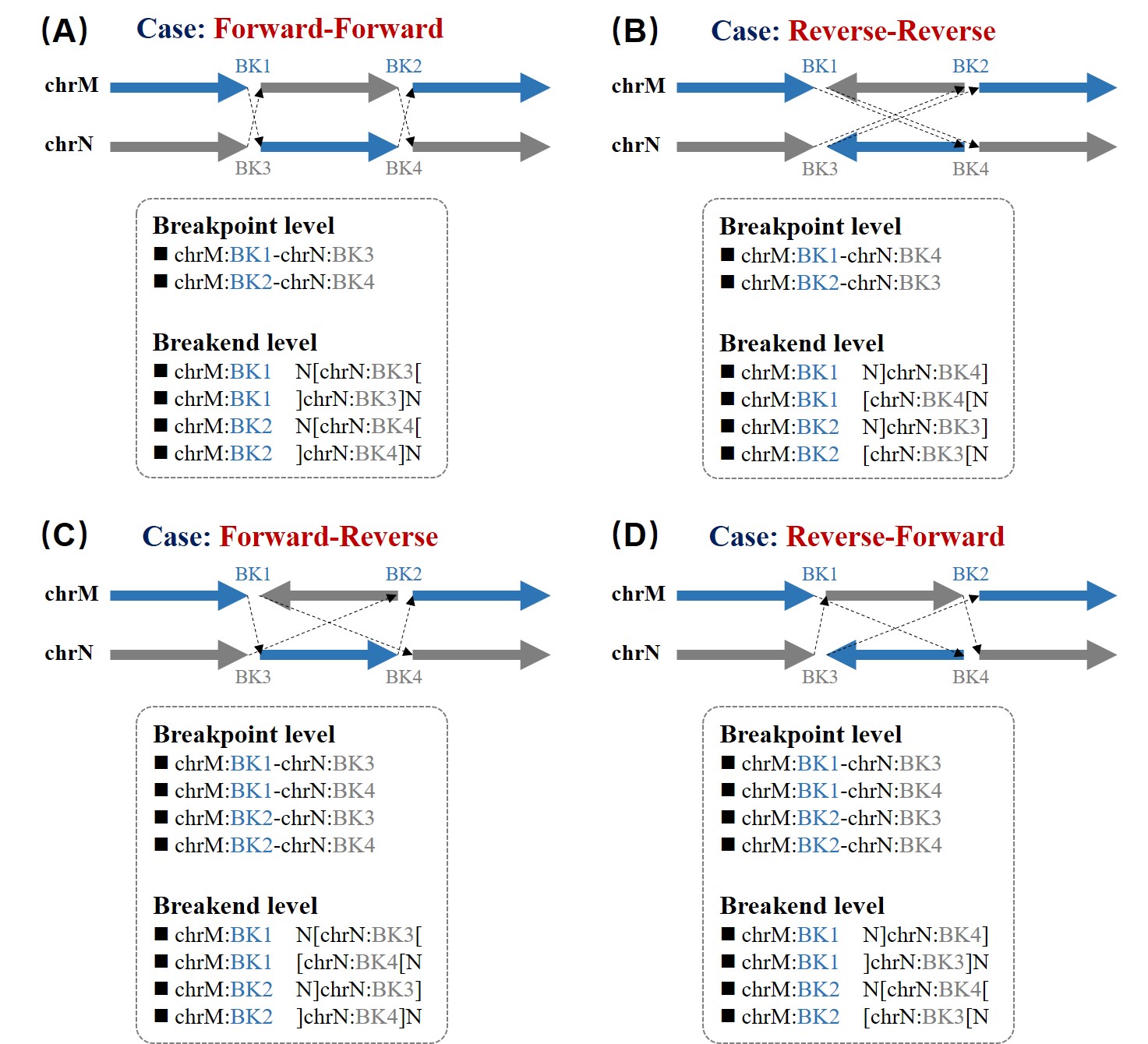


**Fig. S11. The illustration of reciprocal translocation at breakpoint and breakend level**

(A) Forward-forward reciprocal translocation. (B) Reverse-reverse reciprocal translocation. (C) Forward-reverse reciprocal translocation. (D) Reverse-forward reciprocal translocation. BK indicates the coordinate at each breakpoint. Black dashed arrow means the connection of the breakend partners. It worth noting that chrM is smaller than chrN in alphabetically.

**Table S1. Benchmark results of simulated deletions**

| **Number of simulated deletions = 6167** | | | | | | |
| --- | --- | --- | --- | --- | --- | --- |
|  | **TP-call** | **TP-base** | **Total prediction** | **Precision** | **Recall** | **F1** |
| **Coverage = 5×** | | | | | | |
| cuteSV | 4198 | 4249 | 4415 | 95.08% | 68.90% | **79.90%** |
| Sniffles | 3948 | 4015 | 4138 | **95.41%** | 65.10% | 77.40% |
| PBSV | 4031 | 4085 | 4281 | 94.16% | 66.24% | 77.77% |
| SVIM | 4223 | **4267** | 4506 | 93.72% | **69.19%** | 79.61% |
| cuteSV (GT) | 3758 | **3805** | 4415 | **85.12%** | **61.70%** | **71.54%** |
| Sniffles (GT) | 1663 | 1698 | 4138 | 40.19% | 27.53% | 32.68% |
| PBSV (GT) | 3493 | 3531 | 4281 | 81.59% | 57.26% | 67.29% |
| SVIM (GT) | 2511 | 2542 | 4506 | 55.73% | 41.22% | 47.39% |
| **Coverage = 10×** | | | | | | |
| cuteSV | 4793 | 4848 | 5034 | 95.21% | 78.61% | 86.12% |
| Sniffles | 4467 | 4545 | 4673 | **95.59%** | 73.70% | 83.23% |
| PBSV | 4905 | **4959** | 5241 | 93.59% | **80.41%** | **86.50%** |
| SVIM | 4814 | 4862 | 5138 | 93.69% | 78.84% | 85.63% |
| cuteSV (GT) | 4564 | **4616** | 5034 | **90.66%** | **74.85%** | **82.00%** |
| Sniffles (GT) | 1907 | 1942 | 4673 | 40.81% | 31.49% | 35.55% |
| PBSV (GT) | 4509 | 4552 | 5241 | 86.03% | 73.81% | 79.46% |
| SVIM (GT) | 4455 | 4499 | 5138 | 86.71% | 72.95% | 79.24% |
| **Coverage = 20×** | | | | | | |
| cuteSV | 5176 | 5234 | 5456 | 94.87% | 84.87% | **89.59%** |
| Sniffles | 4978 | 5069 | 5231 | **95.16%** | 82.20% | 88.21% |
| PBSV | 5030 | 5082 | 5384 | 93.42% | 82.41% | 87.57% |
| SVIM | 5314 | **5325** | 5742 | 92.55% | **86.35%** | 89.34% |
| cuteSV (GT) | 5026 | **5078** | 5456 | **92.12%** | **82.34%** | **86.96%** |
| Sniffles (GT) | 2263 | 2331 | 5231 | 43.26% | 37.80% | 40.35% |
| PBSV (GT) | 4694 | 4732 | 5384 | 87.18% | 76.73% | 81.62% |
| SVIM (GT) | 4977 | 5002 | 5742 | 86.68% | 81.11% | 83.80% |
| **Coverage = 30×** | | | | | | |
| cuteSV | 5243 | 5310 | 5546 | **94.54%** | 86.10% | **90.12%** |
| Sniffles | 5194 | 5269 | 5495 | 94.52% | 85.44% | 89.75% |
| PBSV | 5024 | 5078 | 5370 | 93.56% | 82.34% | 87.59% |
| SVIM | 5414 | **5417** | 5906 | 91.67% | **87.84%** | 89.71% |
| cuteSV (GT) | 5106 | **5172** | 5546 | **92.07%** | **83.87%** | **87.77%** |
| Sniffles (GT) | 2495 | 2550 | 5495 | 45.40% | 41.35% | 43.28% |
| PBSV (GT) | 4667 | 4699 | 5370 | 86.91% | 76.20% | 81.20% |
| SVIM (GT) | 5055 | 5084 | 5906 | 85.59% | 82.44% | 83.99% |

The best results have been marked in bold respectively. The evaluation was performed by using *eval_sim.py* available at <https://github.com/tjiangHIT/cuteSV>.

**Table S2. Benchmark results of simulated insertions**

| **Number of simulated insertions = 9899** | | | | | | |
| --- | --- | --- | --- | --- | --- | --- |
|  | **TP-call** | **TP-base** | **Total prediction** | **Precision** | **Recall** | **F1** |
| **Coverage = 5×** | | | | | | |
| cuteSV | 5848 | 5934 | 6459 | 90.54% | 59.95% | 72.13% |
| Sniffles | 5157 | 5358 | 5552 | **92.89%** | 54.13% | 68.40% |
| PBSV | 5561 | 5731 | 6533 | 85.12% | 57.89% | 68.92% |
| SVIM | 6266 | **6360** | 7557 | 82.92% | **64.25%** | **72.40%** |
| cuteSV (GT) | 4729 | **4808** | 6459 | **73.22%** | **48.57%** | **58.40%** |
| Sniffles (GT) | 1951 | 2037 | 5552 | 35.14% | 20.58% | 25.96% |
| PBSV (GT) | 4234 | 4360 | 6533 | 64.81% | 44.04% | 52.45% |
| SVIM (GT) | 3090 | 3144 | 7557 | 40.89% | 31.76% | 35.75% |
| **Coverage = 10×** | | | | | | |
| cuteSV | 6551 | 6646 | 7170 | 91.37% | 67.14% | 77.40% |
| Sniffles | 5836 | 6045 | 6237 | **93.57%** | 61.07% | 73.90% |
| PBSV | 7075 | **7290** | 8363 | 84.60% | **73.64%** | **78.74%** |
| SVIM | 7102 | 7181 | 8541 | 83.15% | 72.54% | 77.49% |
| cuteSV (GT) | 5640 | 5728 | 7170 | **78.66%** | 57.86% | **66.68%** |
| Sniffles (GT) | 2250 | 2340 | 6237 | 36.08% | 23.64% | 28.56% |
| PBSV (GT) | 5639 | **5787** | 8363 | 67.43% | **58.46%** | 62.62% |
| SVIM (GT) | 5563 | 5664 | 8541 | 65.13% | 57.22% | 60.92% |
| **Coverage = 20×** | | | | | | |
| cuteSV | 7447 | 7538 | 8186 | 90.97% | 76.15% | **82.90%** |
| Sniffles | 6863 | 7067 | 7434 | **92.32%** | 71.39% | 80.52% |
| PBSV | 7564 | 7805 | 8929 | 84.71% | 78.85% | 81.67% |
| SVIM | 8360 | **8324** | 10532 | 79.38% | **84.09%** | 81.67% |
| cuteSV (GT) | 6644 | **6738** | 8186 | **81.16%** | **68.07%** | **74.04%** |
| Sniffles (GT) | 2757 | 2862 | 7434 | 37.09% | 28.91% | 32.49% |
| PBSV (GT) | 6082 | 6256 | 8929 | 68.12% | 63.20% | 65.56% |
| SVIM (GT) | 6479 | 6579 | 10532 | 61.52% | 66.46% | 63.89% |
| **Coverage = 30×** | | | | | | |
| cuteSV | 7582 | 7679 | 8383 | 90.44% | 77.57% | **83.52%** |
| Sniffles | 7214 | 7447 | 7921 | **91.07%** | 75.23% | 82.40% |
| PBSV | 7585 | 7808 | 9040 | 83.90% | 78.88% | 81.31% |
| SVIM | 8675 | **8540** | 11202 | 77.44% | **86.27%** | 81.62% |
| cuteSV (GT) | 6814 | **6925** | 8383 | **81.28%** | **69.96%** | **75.20%** |
| Sniffles (GT) | 3001 | 3160 | 7921 | 37.89% | 31.92% | 34.65% |
| PBSV (GT) | 6013 | 6186 | 9040 | 66.52% | 62.49% | 64.44% |
| SVIM (GT) | 6497 | 6562 | 11202 | 58.00% | 66.29% | 61.87% |

The best results have been marked in bold respectively. The evaluation was performed by using *eval_sim.py* available at <https://github.com/tjiangHIT/cuteSV>.

**Table S3. Benchmark results of simulated duplications**

| **Number of simulated duplications = 3712** | | | | | | |
| --- | --- | --- | --- | --- | --- | --- |
|  | **TP-call** | **TP-base** | **Total prediction** | **Precision** | **Recall** | **F1** |
| **Coverage = 5×** | | | | | | |
| cuteSV | 2415 | **2400** | 2527 | 95.57% | **64.66%** | **77.13%** |
| Sniffles | 2195 | 2212 | 2283 | **96.15%** | 59.59% | 73.58% |
| PBSV | 2077 | 2089 | 2242 | 92.64% | 56.28% | 70.02% |
| SVIM | 2149 | 2152 | 2318 | 92.71% | 57.97% | 71.34% |
| cuteSV (GT) | 1864 | **1864** | 2527 | **73.76%** | **50.22%** | **59.75%** |
| Sniffles (GT) | 653 | 664 | 2283 | 28.60% | 17.89% | 22.01% |
| PBSV (GT) | 1266 | 1277 | 2242 | 56.47% | 34.40% | 42.76% |
| SVIM (GT) | 664 | 663 | 2318 | 28.65% | 17.86% | 22.00% |
| **Coverage = 10×** | | | | | | |
| cuteSV | 2757 | **2744** | 2849 | 96.77% | **73.92%** | **83.82%** |
| Sniffles | 2451 | 2469 | 2508 | **97.73%** | 66.51% | 79.15% |
| PBSV | 2572 | 2576 | 2721 | 94.52% | 69.40% | 80.03% |
| SVIM | 2449 | 2444 | 2597 | 94.30% | 65.84% | 77.54% |
| cuteSV (GT) | 2183 | **2187** | 2849 | **76.62%** | **58.92%** | **66.61%** |
| Sniffles (GT) | 710 | 726 | 2508 | 28.31% | 19.56% | 23.13% |
| PBSV (GT) | 1696 | 1707 | 2721 | 62.33% | 45.99% | 52.92% |
| SVIM (GT) | 1136 | 1138 | 2597 | 43.74% | 30.66% | 36.05% |
| **Coverage = 20×** | | | | | | |
| cuteSV | 3114 | **3099** | 3241 | 96.08% | **83.49%** | **89.34%** |
| Sniffles | 2736 | 2749 | 2839 | **96.37%** | 74.06% | 83.75% |
| PBSV | 2867 | 2866 | 3022 | 94.87% | 77.21% | 85.13% |
| SVIM | 2788 | 2774 | 3000 | 92.93% | 74.73% | 82.84% |
| cuteSV (GT) | 2557 | **2561** | 3241 | **78.90%** | **68.99%** | **73.61%** |
| Sniffles (GT) | 788 | 791 | 2839 | 27.76% | 21.31% | 24.11% |
| PBSV (GT) | 1988 | 1995 | 3022 | 65.78% | 53.74% | 59.16% |
| SVIM (GT) | 1324 | 1325 | 3000 | 44.13% | 35.70% | 39.47% |
| **Coverage = 30×** | | | | | | |
| cuteSV | 3240 | **3202** | 3391 | 95.55% | **86.26%** | **90.67%** |
| Sniffles | 2859 | 2869 | 2984 | **95.81%** | 77.29% | 85.56% |
| PBSV | 2908 | 2920 | 3076 | 94.54% | 78.66% | 85.87% |
| SVIM | 2877 | 2841 | 3110 | 92.51% | 76.54% | 83.77% |
| cuteSV (GT) | 2648 | **2652** | 3391 | **78.09%** | **71.44%** | **74.62%** |
| Sniffles (GT) | 864 | 873 | 2984 | 28.95% | 23.52% | 25.95% |
| PBSV (GT) | 2073 | 2091 | 3076 | 67.39% | 56.33% | 61.37% |
| SVIM (GT) | 1328 | 1325 | 3110 | 42.70% | 35.70% | 38.88% |

The best results have been marked in bold respectively. The evaluation was performed by using *eval_sim.py* available at <https://github.com/tjiangHIT/cuteSV>.

**Table S4. Benchmark results of simulated inversions**

| **Number of simulated inversions = 44** | | | | | | |
| --- | --- | --- | --- | --- | --- | --- |
|  | **TP-call** | **TP-base** | **Total prediction** | **Precision** | **Recall** | **F1** |
| **Coverage = 5×** | | | | | | |
| cuteSV | 29 | **28** | 34 | **85.29%** | **63.64%** | **72.89%** |
| Sniffles | 27 | 27 | 41 | 65.85% | 61.36% | 63.53% |
| PBSV | 11 | 11 | 16 | 68.75% | 25.00% | 36.67% |
| SVIM | 19 | 19 | 25 | 76.00% | 43.18% | 55.07% |
| cuteSV (GT) | 20 | 19 | 34 | 58.82% | 43.18% | 49.80% |
| Sniffles (GT) | 23 | **23** | 41 | 56.10% | **52.27%** | **54.12%** |
| PBSV (GT) | 10 | 10 | 16 | **62.50%** | 22.73% | 33.33% |
| SVIM (GT) | 8 | 8 | 25 | 32.00% | 18.18% | 23.19% |
| **Coverage = 10×** | | | | | | |
| cuteSV | 28 | **26** | 36 | 77.78% | **59.09%** | **67.16%** |
| Sniffles | 28 | **26** | 41 | 68.29% | **59.09%** | 63.36% |
| PBSV | 14 | 14 | 17 | 82.35% | 31.82% | 45.90% |
| SVIM | 21 | 21 | 24 | **87.50%** | 47.73% | 61.76% |
| cuteSV (GT) | 21 | **19** | 36 | 58.33% | **43.18%** | **49.63%** |
| Sniffles (GT) | 18 | 18 | 41 | 43.90% | 40.91% | 42.35% |
| PBSV (GT) | 13 | 13 | 17 | **76.47%** | 29.55% | 42.62% |
| SVIM (GT) | 15 | 15 | 24 | 62.50% | 34.09% | 44.12% |
| **Coverage = 20×** | | | | | | |
| cuteSV | 33 | **28** | 40 | **82.50%** | **63.64%** | **71.85%** |
| Sniffles | 29 | 27 | 50 | 58.00% | 61.36% | 59.63% |
| PBSV | 15 | 15 | 19 | 78.95% | 34.09% | 47.62% |
| SVIM | 23 | 23 | 29 | 79.31% | 52.27% | 63.01% |
| cuteSV (GT) | 24 | **22** | 40 | 60.00% | **50.00%** | 54.55% |
| Sniffles (GT) | 19 | 18 | 50 | 38.00% | 40.91% | 39.40% |
| PBSV (GT) | 13 | 13 | 19 | 68.42% | 29.55% | 41.27% |
| SVIM (GT) | 22 | **22** | 29 | **75.86%** | **50.00%** | **60.27%** |
| **Coverage = 30×** | | | | | | |
| cuteSV | 36 | **30** | 48 | 75.00% | **68.18%** | **71.43%** |
| Sniffles | 32 | 29 | 67 | 47.76% | 65.91% | 55.39% |
| PBSV | 14 | 14 | 19 | 73.68% | 31.82% | 44.44% |
| SVIM | 23 | 23 | 29 | **79.31%** | 52.27% | 63.01% |
| cuteSV (GT) | 26 | **22** | 48 | 54.17% | **50.00%** | 52.00% |
| Sniffles (GT) | 21 | 21 | 67 | 31.34% | 47.73% | 37.84% |
| PBSV (GT) | 12 | 12 | 19 | 63.16% | 27.27% | 38.10% |
| SVIM (GT) | 22 | **22** | 29 | **75.86%** | **50.00%** | **60.27%** |

The best results have been marked in bold respectively. The evaluation was performed by using *eval_sim.py* available at <https://github.com/tjiangHIT/cuteSV>.

**Table S5. Benchmark results of simulated reciprocal translocations (breakpoint level)**

| **Number of breakpoints of simulated reciprocal translocation = 1008** | | | | | | |
| --- | --- | --- | --- | --- | --- | --- |
|  | **TP-call** | **TP-base** | **Total prediction** | **Precision** | **Recall** | **F1** |
| **Coverage = 5×** | | | | | | |
| cuteSV | 1189 | 839 | 1198 | 99.25% | 83.23% | 90.54% |
| Sniffles | 857 | **849** | 862 | **99.42%** | **84.23%** | **91.19%** |
| PBSV | 2096 | 783 | 2202 | 95.19% | 77.68% | 85.55% |
| SVIM | 544 | 228 | 580 | 93.79% | 22.62% | 36.45% |
| cuteSV (GT) | 1139 | **805** | 1198 | **95.08%** | **79.86%** | **86.81%** |
| Sniffles (GT) | 788 | 780 | 862 | 91.42% | 77.38% | 83.81% |
| PBSV (GT) | 1846 | 691 | 2202 | 83.83% | 68.55% | 75.43% |
| SVIM (GT) | -- | -- | -- | -- | -- | -- |
| **Coverage = 10×** | | | | | | |
| cuteSV | 1326 | 902 | 1329 | **99.77%** | 89.48% | 94.35% |
| Sniffles | 916 | **907** | 920 | 99.57% | **89.98%** | **94.53%** |
| PBSV | 2544 | 874 | 2658 | 95.71% | 86.71% | 90.99% |
| SVIM | 607 | 241 | 642 | 94.55% | 23.91% | 38.17% |
| cuteSV (GT) | 1251 | **849** | 1329 | **94.13%** | **84.23%** | **88.90%** |
| Sniffles (GT) | 852 | 843 | 920 | 92.61% | 83.63% | 87.89% |
| PBSV (GT) | 2327 | 796 | 2658 | 87.55% | 78.97% | 83.04% |
| SVIM (GT) | -- | -- | -- | -- | -- | -- |
| **Coverage = 20×** | | | | | | |
| cuteSV | 1445 | **934** | 1457 | **99.18%** | **92.66%** | **95.81%** |
| Sniffles | 950 | 929 | 959 | 99.06% | 92.16% | 95.49% |
| PBSV | 2622 | 885 | 2742 | 95.62% | 87.80% | 91.54% |
| SVIM | 666 | 254 | 702 | 94.87% | 25.20% | 39.82% |
| cuteSV (GT) | 1340 | 859 | 1457 | 91.97% | 85.22% | 88.47% |
| Sniffles (GT) | 893 | **876** | 959 | **93.12%** | **86.90%** | **89.90%** |
| PBSV (GT) | 2376 | 794 | 2742 | 86.65% | 78.77% | 82.52% |
| SVIM (GT) | -- | -- | -- | -- | -- | -- |
| **Coverage = 30×** | | | | | | |
| cuteSV | 1461 | **940** | 1470 | **99.39%** | **93.25%** | **96.22%** |
| Sniffles | 956 | 937 | 967 | 98.86% | 92.96% | 95.82% |
| PBSV | 2643 | 891 | 2766 | 95.55% | 88.39% | 91.83% |
| SVIM | 702 | 263 | 738 | 95.12% | 26.09% | 40.95% |
| cuteSV (GT) | 1378 | 880 | 1470 | **93.74%** | 87.30% | 90.41% |
| Sniffles (GT) | 905 | **889** | 967 | 93.59% | **88.19%** | **90.81%** |
| PBSV (GT) | 2279 | 767 | 2766 | 82.39% | 76.09% | 79.12% |
| SVIM (GT) | -- | -- | -- | -- | -- | -- |

The best results have been marked in bold respectively. The evaluation was performed by using *eval_sim.py* available at <https://github.com/tjiangHIT/cuteSV>.

**Table S6. Benchmark results of simulated reciprocal translocations (breakend level)**

| **Number of breakends of simulated reciprocal translocation = 1520** | | | | | | |
| --- | --- | --- | --- | --- | --- | --- |
|  | **TP-call** | **TP-base** | **Total prediction** | **Precision** | **Recall** | **F1** |
| **Coverage = 5×** | | | | | | |
| cuteSV | 937 | 877 | 1198 | **78.21%** | 57.70% | 66.41% |
| Sniffles | 195 | 193 | 862 | 22.62% | 12.70% | 16.27% |
| PBSV | 1677 | **922** | 2202 | 76.16% | **60.66%** | **67.53%** |
| SVIM | 475 | 300 | 580 | 81.90% | 19.74% | 31.81% |
| cuteSV (GT) | 910 | **857** | 1198 | **75.96%** | **56.38%** | **64.72%** |
| Sniffles (GT) | 183 | 181 | 862 | 21.23% | 11.91% | 15.26% |
| PBSV (GT) | 1482 | 824 | 2202 | 67.30% | 54.21% | 60.05% |
| SVIM (GT) | -- | -- | -- | -- | -- | -- |
| **Coverage = 10×** | | | | | | |
| cuteSV | 1034 | 965 | 1329 | 77.80% | 63.49% | 69.92% |
| Sniffles | 210 | 205 | 920 | 22.83% | 13.49% | 16.96% |
| PBSV | 2024 | **1093** | 2658 | 76.15% | **71.91%** | **73.97%** |
| SVIM | 534 | 324 | 642 | **83.18%** | 21.32% | 33.94% |
| cuteSV (GT) | 996 | 936 | 1329 | **74.94%** | 61.58% | 67.61% |
| Sniffles (GT) | 200 | 195 | 920 | 21.74% | 12.83% | 16.14% |
| PBSV (GT) | 1870 | **1012** | 2658 | 70.35% | **66.58%** | **68.41%** |
| SVIM (GT) | -- | -- | -- | -- | -- | -- |
| **Coverage = 20×** | | | | | | |
| cuteSV | 1135 | 1042 | 1457 | 77.90% | 68.55% | 72.93% |
| Sniffles | 224 | 213 | 959 | 23.36% | 14.01% | 17.52% |
| PBSV | 2093 | **1122** | 2742 | 76.33% | **73.82%** | **75.05%** |
| SVIM | 588 | 346 | 702 | **83.76%** | 22.76% | 35.80% |
| cuteSV (GT) | 1072 | 996 | 1457 | **73.58%** | 65.53% | **69.32%** |
| Sniffles (GT) | 216 | 206 | 959 | 22.52% | 13.55% | 16.92% |
| PBSV (GT) | 1904 | **1028** | 2742 | 69.44% | **67.63%** | 68.52% |
| SVIM (GT) | -- | -- | -- | -- | -- | -- |
| **Coverage = 30×** | | | | | | |
| cuteSV | 1149 | 1054 | 1470 | 78.16% | 69.34% | 73.49% |
| Sniffles | 227 | 216 | 967 | 23.47% | 14.21% | 17.70% |
| PBSV | 2108 | **1130** | 2766 | 76.21% | **74.34%** | **75.27%** |
| SVIM | 620 | 361 | 738 | **84.01%** | 23.75% | 37.03% |
| cuteSV (GT) | 1102 | **1021** | 1470 | **74.97%** | **67.17%** | **70.85%** |
| Sniffles (GT) | 214 | 204 | 967 | 22.13% | 13.42% | 16.71% |
| PBSV (GT) | 1820 | 983 | 2766 | 65.80% | 64.67% | 65.23% |
| SVIM (GT) | -- | -- | -- | -- | -- | -- |

The best results have been marked in bold respectively. The evaluation was performed by using *eval_sim.py* available at <https://github.com/tjiangHIT/cuteSV>.

**Table S7. Genotype confusion matrices of callsets with different SV callers on 30× simulated datasets**

| **Deletion** | | | | | | | | | | | | | |
| --- | --- | --- | --- | --- | --- | --- | --- | --- | --- | --- | --- | --- | --- |
|  | | cuteSV  calls = 5546 | | | Sniffles  calls = 5495 | | | PBSV  calls = 5370 | | | SVIM  calls = 5906 | | |
|  |  | 0/0 | 0/1 | 1/1 | 0/0 | 0/1 | 1/1 | 0/0 | 0/1 | 1/1 | 0/0 | 0/1 | 1/1 |
| Ground-truth | 0/0 | 5 | 165 | 133 | 74 | 193 | 34 | 0 | 206 | 140 | 20 | 264 | 208 |
|  | 0/1 | 6 | 2751 | 4 | 532 | 2154 | 147 | 0 | 2555 | 25 | 34 | 2757 | 0 |
|  | 1/1 | 1 | 126 | 2355 | 7 | 2013 | 341 | 0 | 332 | 2112 | 26 | 299 | 2298 |
| **Insertion** | | | | | | | | | | | | | |
|  | | cuteSV  calls = 8383 | | | Sniffles  calls = 7921 | | | PBSV  calls = 9040 | | | SVIM  calls = 11202 | | |
|  |  | 0/0 | 0/1 | 1/1 | 0/0 | 0/1 | 1/1 | 0/0 | 0/1 | 1/1 | 0/0 | 0/1 | 1/1 |
| Ground-truth | 0/0 | 62 | 520 | 219 | 226 | 360 | 121 | 0 | 1218 | 237 | 940 | 1451 | 136 |
|  | 0/1 | 65 | 3918 | 2 | 1054 | 2527 | 189 | 0 | 3766 | 11 | 299 | 4034 | 2 |
|  | 1/1 | 11 | 690 | 2896 | 87 | 2883 | 474 | 0 | 1561 | 2247 | 190 | 1687 | 2463 |
| **Duplication** | | | | | | | | | | | | | |
|  | | cuteSV  calls = 3391 | | | Sniffles  calls = 2984 | | | PBSV  calls = 3076 | | | SVIM  calls = 3110 | | |
|  |  | 0/0 | 0/1 | 1/1 | 0/0 | 0/1 | 1/1 | 0/0 | 0/1 | 1/1 | 0/0 | 0/1 | 1/1 |
| Ground-truth | 0/0 | 60 | 84 | 7 | 47 | 62 | 16 | 0 | 97 | 11 | 132 | 97 | 0 |
|  | 0/1 | 27 | 1127 | 367 | 462 | 821 | 27 | 0 | 1434 | 17 | 26 | 789 | 0 |
|  | 1/1 | 27 | 171 | 1521 | 124 | 1382 | 43 | 0 | 818 | 699 | 19 | 165 | 539 |
| **Inversion** | | | | | | | | | | | | | |
|  | | cuteSV  calls = 48 | | | Sniffles  calls = 67 | | | PBSV  calls = 19 | | | SVIM  calls = 29 | | |
|  |  | 0/0 | 0/1 | 1/1 | 0/0 | 0/1 | 1/1 | 0/0 | 0/1 | 1/1 | 0/0 | 0/1 | 1/1 |
| Ground-truth | 0/0 | 0 | 11 | 1 | 4 | 20 | 11 | 0 | 4 | 1 | 0 | 5 | 0 |
|  | 0/1 | 0 | 13 | 9 | 5 | 14 | 2 | 0 | 9 | 1 | 0 | 16 | 0 |
|  | 1/1 | 0 | 1 | 13 | 1 | 3 | 7 | 0 | 1 | 3 | 1 | 0 | 7 |
| **Reciprocal translocation (breakpoint level)** | | | | | | | | | | | | | |
|  | | cuteSV  calls = 1470 | | | Sniffles  calls = 967 | | | PBSV  calls = 2766 | | | SVIM  calls = 738 | | |
|  |  | 0/0 | 0/1 | 1/1 | 0/0 | 0/1 | 1/1 | 0/0 | 0/1 | 1/1 | 0/0 | 0/1 | 1/1 |
| Ground-truth | 0/0 | 5 | 14 | 0 | 2 | 7 | 2 | 0 | 33 | 90 | -- | -- | -- |
|  | 0/1 | 33 | 1107 | 0 | 8 | 422 | 38 | 0 | 964 | 222 | -- | -- | -- |
|  | 1/1 | 9 | 31 | 271 | 0 | 5 | 483 | 0 | 142 | 1315 | -- | -- | -- |
| **Reciprocal translocation (breakend level)** | | | | | | | | | | | | | |
|  | | cuteSV  calls = 1470 | | | Sniffles  calls = 967 | | | PBSV  calls = 2766 | | | SVIM  calls = 738 | | |
|  |  | 0/0 | 0/1 | 1/1 | 0/0 | 0/1 | 1/1 | 0/0 | 0/1 | 1/1 | 0/0 | 0/1 | 1/1 |
| Ground-truth | 0/0 | 11 | 266 | 44 | 5 | 327 | 408 | 0 | 283 | 375 | -- | -- | -- |
|  | 0/1 | 29 | 875 | 0 | 5 | 106 | 7 | 0 | 759 | 191 | -- | -- | -- |
|  | 1/1 | 7 | 11 | 227 | 0 | 1 | 108 | 0 | 97 | 1061 | -- | -- | -- |

For column, 0/0, 0/1, 1/1 indicate the call determined as the homozygous reference variant, heterozygous variant and homozygous alternative variant by SV caller, respectively. For row, 0/0 means false positive call, and 0/1, 1/1 indicate the heterozygous variant and homozygous alternative variant in the ground truth. In these matrices, a figure in a specific position indicates the number of calls which are determined as column-genotype by SV caller but are the row-genotype actually in the ground truth. It is worth noting that SVIM does not assign genotypes for all the translocations and most of the duplications, we skipped these calls for zygosity analysis.

**Table S8. The distinction of accuracy between cigar-only calls and split-alignment-only calls on 30× simulated datasets**

|  | **Cigar-only** | **Split-alignment-only** | **Complete** | **Cigar-only / Complete** | **Split-alignment-only / Complete** |
| --- | --- | --- | --- | --- | --- |
| **Number of simulated deletions = 6167** | | | | | |
| TP-base | 4969 | 109 | 5310 | 93.59% | 2.05% |
| TP-base (GT) | 4851 | 100 | 5172 | 93.80% | 1.94% |
| **Number of simulated insertions = 9899** | | | | | |
| TP-base | 7386 | 254 | 7679 | 96.18% | 3.31% |
| TP-base (GT) | 6749 | 75 | 6925 | 97.47% | 1.08% |

TP-base and TP-base (GT) indicate true positive ground truth sets without and with genotype, respectively. Column 2 to 4 indicate the numbers of correctly detected ground truth sets with the corresponding types of signatures. Column 5 and 6 indicate the ratios of true positive calls produced by the corresponding types of signatures to all the true positive calls.

**Table S9. Benchmark results on** **the 69× HG002 PacBio CLR datasets**

| **Tool** | **cuteSV** | **Sniffles** | **PBSV** | **SVIM** | **cuteSV (GT)** | **Sniffles (GT)** | **PBSV (GT)** | **SVIM (GT)** |
| --- | --- | --- | --- | --- | --- | --- | --- | --- |
| Coverage = 5x | | | | | | | | |
| Precision | 80.50% | 48.65% | **94.55%** | 58.00% | 66.35% | 21.18% | **72.00%** | 25.86% |
| Recall | 56.45% | 52.54% | 41.93% | **57.46%** | **51.65%** | 32.52% | 35.47% | 37.59% |
| F1 | **66.36%** | 50.52% | 58.09% | 57.73% | **58.08%** | 25.65% | 47.53% | 30.64% |
| Coverage = 10x | | | | | | | | |
| Precision | **96.19%** | 90.10% | 93.78% | 91.01% | **85.33%** | 38.76% | 73.46% | 78.15% |
| Recall | 69.12% | 61.49% | **69.48%** | 68.70% | **66.51%** | 40.72% | 64.07% | 65.33% |
| F1 | **80.44%** | 73.09% | 79.83% | 78.30% | **74.76%** | 39.71% | 68.48% | 71.17% |
| Coverage = 20x | | | | | | | | |
| Precision | **96.21%** | 94.88% | 93.45% | 92.06% | **87.84%** | 42.06% | 72.07% | 84.29% |
| Recall | **85.21%** | 75.02% | 81.90% | 83.25% | **84.03%** | 57.11% | 77.73% | 81.98% |
| F1 | **90.37%** | 83.79% | 87.30% | 87.43% | **85.89%** | 48.44% | 74.79% | 83.12% |
| Coverage = 30x | | | | | | | | |
| Precision | **93.50%** | 89.29% | **93.50%** | 91.87% | **84.80%** | 41.63% | 70.93% | 84.53% |
| Recall | **92.55%** | 84.56% | 83.77% | 87.32% | **91.85%** | 71.85% | 79.65% | 86.37% |
| F1 | **93.03%** | 86.86% | 88.37% | 89.54% | **88.18%** | 52.72% | 75.04% | 85.44% |
| Coverage = 40x | | | | | | | | |
| Precision | **94.18%** | 92.46% | 93.10% | 91.33% | **86.23%** | 43.87% | 69.27% | 83.76% |
| Recall | **94.05%** | 87.25% | 85.01% | 88.97% | **93.53%** | 76.46% | 80.84% | 88.10% |
| F1 | **94.11%** | 89.78% | 88.87% | 90.14% | **89.73%** | 55.75% | 74.61% | 85.87% |
| Coverage = 69x | | | | | | | | |
| Precision | **94.78%** | 93.68% | 93.14% | 92.70% | **89.24%** | 40.92% | 68.13% | 85.22% |
| Recall | **94.09%** | 86.27% | 88.42% | 89.56% | **93.74%** | 73.29% | 84.82% | 88.74% |
| F1 | **94.43%** | 89.82% | 90.72% | 91.10% | **91.44%** | 52.52% | 75.56% | 86.94% |

The best results have been marked in bold respectively. The evaluation was performed by using *Truvari* available at https://github.com/spiralgenetics/truvari.

**Table S10. Benchmark results on** **the 28× HG002 PacBio CCS datasets**

| **Tool** | **cuteSV** | **Sniffles** | **PBSV** | **SVIM** | **cuteSV (GT)** | **Sniffles (GT)** | **PBSV (GT)** | **SVIM (GT)** |
| --- | --- | --- | --- | --- | --- | --- | --- | --- |
| Coverage = 5x | | | | | | | | |
| Precision | 90.87% | 88.07% | **92.28%** | 85.86% | 74.32% | 39.16% | **78.83%** | 43.52% |
| Recall | **91.87%** | 86.71% | 91.40% | 89.92% | **90.23%** | 74.37% | 90.08% | 81.89% |
| F1 | 91.37% | 87.39% | **91.84%** | 87.84% | 81.51% | 51.30% | **84.08%** | 56.83% |
| Coverage = 10x | | | | | | | | |
| Precision | **95.27%** | 94.14% | 93.15% | 91.15% | **86.31%** | 43.65% | 83.53% | 78.95% |
| Recall | 93.79% | 88.08% | **96.70%** | 91.70% | 93.19% | 77.41% | **96.34%** | 90.54% |
| F1 | 94.52% | 91.01% | **94.89%** | 91.43% | **89.62%** | 55.83% | 89.48% | 84.35% |
| Coverage = 28x | | | | | | | | |
| Precision | 94.59% | 93.65% | **94.62%** | 88.89% | **90.18%** | 45.44% | 86.62% | 84.54% |
| Recall | **98.02%** | 93.78% | **98.02%** | 95.60% | **97.92%** | 88.20% | 97.84% | 95.39% |
| F1 | 96.28% | 93.71% | **96.29%** | 92.12% | **93.89%** | 60.84% | 91.89% | 89.64% |

The best results have been marked in bold respectively. The evaluation was performed by using *Truvari* available at https://github.com/spiralgenetics/truvari.

**Table S11. Benchmark results on** **Ashkenazi human trio**

|  | **cuteSV** | **Sniffles** | **PBSV** | **SVIM** |
| --- | --- | --- | --- | --- |
| **Statistics of HG003** | | | | |
| homozygote | 11431 | 9314 | 12449 | 8182 |
| Proven calls | 10882 | 7416 | 9911 | 7812 |
| Recall | 95.20% | 79.62% | 79.61% | **95.48%** |
| **Statistics of HG004** | | | | |
| homozygote | 10759 | 9659 | 10447 | 7682 |
| Proven calls | 10204 | 7805 | 8871 | 7431 |
| Recall | 94.84% | 80.81% | 84.91% | **96.73%** |
| **Statistics of HG002** | | | | |
| Total calls | 33060 | 36117 | 22935 | 33786 |
| Not in parents | 2432 | 6302 | 2122 | 2576 |
| MDR | **7.36%** | 17.45% | 9.25% | 7.62% |

The best results have been marked in bold respectively. The evaluation was performed by using *eval_trio.py* available at <https://github.com/tjiangHIT/cuteSV>.

**Table S12. Benchmark results on** **the 47× HG002 ONT PromethION datasets**

| **Tool** | **cuteSV** | **Sniffles** | **PBSV** | **SVIM** | **cuteSV (GT)** | **Sniffles (GT)** | **PBSV (GT)** | **SVIM (GT)** |
| --- | --- | --- | --- | --- | --- | --- | --- | --- |
| Coverage = 5x | | | | | | | | |
| Precision | **90.63%** | 83.74% | 75.45% | 78.43% | **74.63%** | 46.15% | 62.46% | 43.80% |
| Recall | **74.44%** | 66.11% | 54.37% | 72.33% | **70.58%** | 51.81% | 49.66% | 59.34% |
| F1 | **81.74%** | 73.89% | 63.20% | 75.25% | **72.55%** | 48.82% | 55.33% | 50.40% |
| Coverage = 10x | | | | | | | | |
| Precision | **93.07%** | 87.66% | 81.86% | 81.11% | **83.23%** | 51.42% | 70.96% | 70.09% |
| Recall | **85.00%** | 74.94% | 78.20% | 81.99% | **83.52%** | 63.69% | 75.66% | 79.74% |
| F1 | **88.85%** | 80.80% | 79.99% | 81.55% | **83.37%** | 56.90% | 73.23% | 74.60% |
| Coverage = 20x | | | | | | | | |
| Precision | **92.22%** | 84.78% | 88.60% | 76.60% | **85.72%** | 52.21% | 77.75% | 67.49% |
| Recall | **94.49%** | 84.78% | 83.52% | 91.39% | **94.10%** | 77.43% | 81.64% | 90.34% |
| F1 | **93.34%** | 84.78% | 85.98% | 83.35% | **89.71%** | 62.37% | 79.65% | 77.26% |
| Coverage = 47x | | | | | | | | |
| Precision | **92.14%** | 84.63% | -- | 85.95% | **88.67%** | 52.69% | -- | 76.16% |
| Recall | **96.61%** | 89.46% | -- | 91.72% | **96.48%** | 84.09% | -- | 90.76% |
| F1 | **94.32%** | 86.98% | -- | 88.74% | **92.41%** | 64.78% | -- | 82.82% |

The best results have been marked in bold respectively. The best results have been marked in bold respectively. The evaluation was performed by using *Truvari* available at <https://github.com/spiralgenetics/truvari>.

**Table S13. Benchmark results of cuteSV callsets with various sequencing technologies datasets on HG002**

| **Dataset** | **Deletion** | **Insertion** | **Duplication** | **Inversion** | **Translocation** | **ALL** |
| --- | --- | --- | --- | --- | --- | --- |
| CLR | 15512 | 22539 | 2987 | 348 | 1346 | 42732 |
| ONT | 30389 | 18566 | 1390 | 145 | 1073 | 51563 |
| CCS | 20262 | 24468 | 2326 | 316 | 2684 | 50056 |
| CLR $\cap$ ONT | 13309 | 14346 | 497 | 115 | 445 | 28712 |
| CLR $\cap$ CCS | 14261 | 18126 | 979 | 164 | 831 | 34361 |
| ONT $\cap$ CCS | 14371 | 15647 | 684 | 130 | 648 | 31340 |
| CLR $\cap$ ONT $\cap$ CCS | 12897 | 13910 | 454 | 114 | 411 | 27786 |

CLR, ONT and CCS indicate the SV callsets of cuteSV generated from the corresponding sequencing platforms. The assessment was performed by using *multi_platform.py*, available at [https://github.com/tjiangHIT/cuteSV](https://github.com/tjiangHIT/cuteSV/src/benchmarks).

**Table S14. Benchmark results of callsets from different tools on various sequencing technologies**

| **Tool** | **HG002 CLR** | **HG002 CCS** | **HG002 ONT** |
| --- | --- | --- | --- |
| cuteSV | 42732 | 50056 | 51563 |
| Sniffles | 40205 | 47366 | 46792 |
| PBSV | 59029 | 67739 | -- |
| SVIM | 47453 | 63973 | 50987 |
| cuteSV$\cap$Sniffles | 32637 | 37625 | 38180 |
| cuteSV$\cap$PBSV | 27757 | 36222 | -- |
| cuteSV$\cap$SVIM | 34734 | 44986 | 42946 |
| Sniffles$\cap$PBSV | 24520 | 31697 | -- |
| Sniffles$\cap$SVIM | 32954 | 41933 | 39457 |
| PBSV$\cap$SVIM | 27644 | 41082 | -- |
| cuteSV $\cap$ Sniffles $\cap$ PBSV | 23306 | 30226 | -- |
| cuteSV $\cap$ Sniffles $\cap$ SVIM | 30226 | 36896 | 35592 |
| cuteSV $\cap$ PBSV $\cap$ SVIM | 25486 | 33945 | -- |
| Sniffles $\cap$PBSV $\cap$ SVIM | 23735 | 31108 | -- |
| cuteSV $\cap$ Sniffles $\cap$ PBSV $\cap$ SVIM | 22804 | 29773 | -- |

The assessment was performed by using *sta_venn.py*, available at [https://github.com/tjiangHIT/cuteSV](https://github.com/tjiangHIT/cuteSV/src/benchmarks).

**Table S15. The results of runtime and memory footprint of various sequencing technologies datasets on HG002**

| **Dataset** | **Thread** | **cuteSV**  **(skip GT)** | **Sniffles**  **(skip GT)** | **SVIM**  **(skip GT)** | **cuteSV** | **Sniffles** | **PBSV** | **SVIM** |
| --- | --- | --- | --- | --- | --- | --- | --- | --- |
| **Elapsed Time (Minute)** | | | | | | | | |
| PacBio CLR  Coverage = 69x | t1 | 882 | **511** | 641 | 1059 | **471** | 943 | 759 |
|  | t2 | **485** | 535 | -- | **557** | 565 | 912 | -- |
|  | t4 | **273** | 595 | -- | **304** | 440 | 906 | -- |
|  | t8 | **176** | 621 | -- | **194** | 621 | 892 | -- |
|  | t16 | **103** | 870 | -- | **132** | 513 | 888 | -- |
| ONT  Coverage = 47x | t1 | 368 | 381 | **246** | 683 | **348** | -- | 901 |
|  | t2 | **201** | 451 | -- | **380** | 422 | -- | -- |
|  | t4 | **115** | 318 | -- | **236** | 327 | -- | -- |
|  | t8 | **75** | 641 | -- | **145** | 484 | -- | -- |
|  | t16 | **64** | 816 | -- | **96** | 607 | -- | -- |
| PacBio CCS  Coverage = 28x | t1 | 50 | 79 | **48** | 110 | **79** | 143 | 96 |
|  | t2 | **34** | 76 | -- | **65** | 94 | 122 | -- |
|  | t4 | **18** | 89 | -- | **36** | 105 | 112 | -- |
|  | t8 | **10** | 84 | -- | **27** | 75 | 107 | -- |
|  | t16 | **6** | 94 | -- | **17** | 152 | 106 | -- |
| **Memory footprint (Gigabyte)** | | | | | | | | |
| PacBio CLR  Coverage = 69x | t1 | **0.37** | 23.74 | 18.23 | **0.37** | 23.72 | 9.78 | 18.23 |
|  | t2 | **0.37** | 25.44 | -- | **0.37** | 25.57 | 9.78 | -- |
|  | t4 | **0.37** | 24.42 | -- | **0.37** | 23.80 | 10.73 | -- |
|  | t8 | **0.37** | 24.43 | -- | **0.37** | 23.12 | 13.73 | -- |
|  | t16 | **0.37** | 23.62 | -- | **0.37** | 24.20 | 13.33 | -- |
| ONT  Coverage = 47x | t1 | **0.23** | 5.13 | 3.10 | **0.23** | 5.20 | -- | 3.10 |
|  | t2 | **0.23** | 5.13 | -- | **0.23** | 5.14 | -- | -- |
|  | t4 | **0.23** | 5.04 | -- | **0.23** | 5.15 | -- | -- |
|  | t8 | **0.23** | 5.10 | -- | **0.23** | 5.10 | -- | -- |
|  | t16 | **0.23** | 5.19 | -- | **0.23** | 5.12 | -- | -- |
| PacBio CCS  Coverage = 28x | t1 | **0.11** | 1.82 | 0.91 | **0.11** | 1.87 | 5.84 | 0.91 |
|  | t2 | **0.11** | 1.76 | -- | **0.11** | 1.82 | 5.85 | -- |
|  | t4 | **0.11** | 1.88 | -- | **0.11** | 1.76 | 5.85 | -- |
|  | t8 | **0.11** | 1.71 | -- | **0.11** | 1.72 | 5.85 | -- |
|  | t16 | **0.11** | 1.78 | -- | **0.11** | 1.83 | 5.85 | -- |

The best results have been marked in bold respectively. The evaluation was performed by using */usr/bin/time -v* in Linux operating system.

**Table S16. The number of false negative calls of HG002 sample under different methods**

| **SV size** | **PBMM2** | | | |  | **NGMLR** | | | |
| --- | --- | --- | --- | --- | --- | --- | --- | --- | --- |
|  | **cuteSV** | **Sniffles** | **PBSV** | **SVIM** |  | **cuteSV** | **Sniffles** | **PBSV** | **SVIM** |
| (-$\infty$, -10000] | 1 | 12 | 1 | 2 |  | 2 | 0 | 3 | 3 |
| (-10000, -9000] | 1 | 4 | 1 | 2 |  | 1 | 0 | 0 | 1 |
| (-9000, -8000] | 0 | 2 | 1 | 0 |  | 1 | 0 | 0 | 2 |
| (-8000, -7000] | 0 | 0 | 0 | 0 |  | 0 | 0 | 0 | 0 |
| (-7000, -6000] | 1 | 32 | 2 | 6 |  | 1 | 1 | 3 | 2 |
| (-6000, -5000] | 1 | 23 | 0 | 7 |  | 3 | 3 | 1 | 4 |
| (-5000, -4000] | 3 | 24 | 2 | 10 |  | 2 | 1 | 2 | 4 |
| (-4000, -3000] | 11 | 45 | 4 | 24 |  | 0 | 0 | 1 | 7 |
| (-3000, -2000] | 18 | 51 | 8 | 34 |  | 4 | 4 | 3 | 6 |
| (-2000, -1000] | 57 | 78 | 44 | 72 |  | 4 | 5 | 11 | 6 |
| (-1000, -900] | 4 | 5 | 5 | 4 |  | 1 | 2 | 4 | 2 |
| (-900, -800] | 1 | 2 | 6 | 1 |  | 0 | 1 | 2 | 2 |
| (-800, -700] | 5 | 4 | 10 | 7 |  | 3 | 0 | 4 | 1 |
| (-700, -600] | 4 | 5 | 8 | 4 |  | 0 | 2 | 2 | 1 |
| (-600, -500] | 3 | 4 | 6 | 2 |  | 2 | 2 | 2 | 2 |
| (-500, -400] | 0 | 2 | 9 | 5 |  | 0 | 2 | 2 | 3 |
| (-400, -300] | 5 | 8 | 32 | 7 |  | 6 | 14 | 28 | 7 |
| (-300, -200] | 6 | 6 | 20 | 5 |  | 5 | 5 | 10 | 5 |
| (-200, -100] | 9 | 19 | 29 | 8 |  | 9 | 28 | 43 | 11 |
| (-100, -50] | 25 | 185 | 93 | 30 |  | 38 | 260 | 101 | 38 |
| [50, 100) | 24 | 90 | 164 | 24 |  | 29 | 158 | 140 | 38 |
| [100, 200) | 16 | 36 | 85 | 27 |  | 20 | 45 | 90 | 26 |
| [200, 300) | 8 | 24 | 42 | 18 |  | 23 | 36 | 140 | 30 |
| [300, 400) | 6 | 21 | 104 | 20 |  | 43 | 46 | 375 | 46 |
| [400, 500) | 11 | 18 | 30 | 20 |  | 30 | 21 | 53 | 20 |
| [500, 600) | 8 | 19 | 14 | 12 |  | 22 | 17 | 38 | 16 |
| [600, 700) | 7 | 16 | 21 | 14 |  | 30 | 19 | 41 | 16 |
| [700, 800) | 13 | 13 | 17 | 15 |  | 15 | 17 | 25 | 20 |
| [800, 900) | 7 | 13 | 22 | 18 |  | 13 | 13 | 22 | 13 |
| [900, 1000) | 6 | 13 | 13 | 14 |  | 13 | 15 | 12 | 13 |
| [1000, 2000) | 110 | 169 | 154 | 204 |  | 83 | 181 | 89 | 207 |
| [2000, 3000) | 49 | 130 | 46 | 143 |  | 35 | 110 | 19 | 150 |
| [3000, 4000) | 35 | 80 | 28 | 78 |  | 29 | 68 | 20 | 78 |
| [4000, 5000) | 26 | 47 | 12 | 46 |  | 26 | 44 | 15 | 45 |
| [5000, 6000) | 9 | 18 | 6 | 18 |  | 9 | 17 | 9 | 18 |
| [6000, 7000) | 40 | 58 | 36 | 58 |  | 47 | 55 | 34 | 58 |
| [7000, 8000) | 5 | 14 | 6 | 14 |  | 11 | 15 | 13 | 14 |
| [8000, 9000) | 7 | 7 | 6 | 7 |  | 7 | 7 | 7 | 7 |
| [9000, 10000) | 5 | 5 | 5 | 5 |  | 5 | 5 | 5 | 5 |
| [10000, +$\infty$) | 23 | 22 | 24 | 22 |  | 23 | 22 | 24 | 22 |

The evaluation was performed using scripts refer to <https://github.com/PacificBiosciences/sv-benchmark>.

**Table S17. The number of false positive calls of HG002 sample under different methods**

| **SV size** | **PBMM2** | | | |  | **NGMLR** | | | |
| --- | --- | --- | --- | --- | --- | --- | --- | --- | --- |
|  | **cuteSV** | **Sniffles** | **PBSV** | **SVIM** |  | **cuteSV** | **Sniffles** | **PBSV** | **SVIM** |
| (-$\infty$, -10000] | 2 | 1 | 7 | 1 |  | 3 | 1 | 3 | 1 |
| (-10000, -9000] | 0 | 0 | 0 | 0 |  | 1 | 0 | 0 | 1 |
| (-9000, -8000] | 2 | 0 | 2 | 2 |  | 2 | 1 | 1 | 1 |
| (-8000, -7000] | 0 | 0 | 1 | 0 |  | 0 | 0 | 0 | 0 |
| (-7000, -6000] | 1 | 0 | 2 | 1 |  | 1 | 0 | 0 | 1 |
| (-6000, -5000] | 0 | 0 | 2 | 1 |  | 2 | 1 | 1 | 2 |
| (-5000, -4000] | 0 | 0 | 1 | 0 |  | 0 | 0 | 0 | 0 |
| (-4000, -3000] | 1 | 0 | 3 | 1 |  | 0 | 0 | 0 | 0 |
| (-3000, -2000] | 2 | 0 | 2 | 2 |  | 0 | 1 | 2 | 0 |
| (-2000, -1000] | 0 | 1 | 5 | 0 |  | 1 | 5 | 12 | 4 |
| (-1000, -900] | 1 | 1 | 1 | 1 |  | 0 | 0 | 1 | 0 |
| (-900, -800] | 0 | 2 | 4 | 0 |  | 1 | 0 | 5 | 1 |
| (-800, -700] | 1 | 0 | 2 | 0 |  | 0 | 0 | 0 | 0 |
| (-700, -600] | 1 | 2 | 5 | 3 |  | 0 | 0 | 3 | 3 |
| (-600, -500] | 0 | 1 | 5 | 1 |  | 0 | 0 | 3 | 2 |
| (-500, -400] | 1 | 3 | 4 | 3 |  | 1 | 1 | 4 | 5 |
| (-400, -300] | 4 | 4 | 16 | 2 |  | 0 | 0 | 18 | 3 |
| (-300, -200] | 4 | 11 | 13 | 11 |  | 5 | 4 | 14 | 4 |
| (-200, -100] | 15 | 25 | 41 | 30 |  | 9 | 18 | 22 | 19 |
| (-100, -50] | 69 | 59 | 97 | 77 |  | 47 | 46 | 77 | 60 |
| [50, 100) | 273 | 187 | 282 | 286 |  | 205 | 151 | 250 | 215 |
| [100, 200) | 67 | 120 | 51 | 121 |  | 48 | 74 | 40 | 109 |
| [200, 300) | 23 | 58 | 12 | 48 |  | 6 | 44 | 5 | 89 |
| [300, 400) | 17 | 33 | 12 | 34 |  | 9 | 64 | 2 | 57 |
| [400, 500) | 3 | 26 | 5 | 18 |  | 0 | 26 | 6 | 48 |
| [500, 600) | 4 | 10 | 10 | 11 |  | 1 | 24 | 27 | 16 |
| [600, 700) | 5 | 4 | 5 | 8 |  | 1 | 15 | 14 | 19 |
| [700, 800) | 0 | 3 | 4 | 4 |  | 0 | 17 | 19 | 12 |
| [800, 900) | 0 | 2 | 1 | 2 |  | 0 | 8 | 12 | 10 |
| [900, 1000) | 0 | 4 | 3 | 0 |  | 0 | 9 | 8 | 2 |
| [1000, 2000) | 3 | 2 | 17 | 6 |  | 7 | 22 | 27 | 13 |
| [2000, 3000) | 1 | 1 | 6 | 3 |  | 1 | 2 | 5 | 1 |
| [3000, 4000) | 0 | 0 | 5 | 0 |  | 0 | 3 | 1 | 0 |
| [4000, 5000) | 0 | 0 | 2 | 0 |  | 0 | 0 | 0 | 0 |
| [5000, 6000) | 0 | 1 | 0 | 2 |  | 0 | 1 | 0 | 1 |
| [6000, 7000) | 0 | 0 | 0 | 1 |  | 0 | 1 | 0 | 0 |
| [7000, 8000) | 0 | 0 | 0 | 0 |  | 0 | 0 | 0 | 0 |
| [8000, 9000) | 0 | 0 | 0 | 0 |  | 0 | 0 | 0 | 0 |
| [9000, 10000) | 0 | 0 | 0 | 0 |  | 0 | 0 | 0 | 0 |
| [10000, +$\infty$) | 0 | 0 | 0 | 0 |  | 0 | 0 | 0 | 0 |

The evaluation was performed using scripts refer to <https://github.com/PacificBiosciences/sv-benchmark>.

**Table S18. The precision and recall of HG002 sample for various sequencing coverages and the configurations of *--min_support* parameter**

| **Coverage** | **Support-read** | **Precision** | **Recall** |  | **Coverage** | **Support-read** | **Precision** | **Recall** |
| --- | --- | --- | --- | --- | --- | --- | --- | --- |
| 5× | -s1 | 2.38% | 83.27% |  | 30× | -s1 | 0.50% | 97.79% |
|  | -s2 | 80.50% | 56.45% |  |  | -s2 | 19.13% | 96.26% |
|  | -s3 | 98.27% | 35.95% |  |  | -s3 | 77.87% | 94.48% |
|  | -s4 | 99.27% | 21.16% |  |  | -s4 | 93.50% | 92.55% |
|  | -s5 | 99.30% | 11.75% |  |  | -s5 | 96.10% | 89.92% |
|  | -s6 | 99.08% | 6.69% |  |  | -s6 | 96.36% | 87.53% |
|  | -s7 | 98.53% | 3.47% |  |  | -s7 | 96.83% | 84.04% |
|  | -s8 | 99.39% | 1.70% |  |  | -s8 | 97.17% | 79.65% |
|  | -s9 | 98.41% | 0.64% |  |  | -s9 | 97.50% | 74.53% |
|  | -s10 | 96.97% | 0.32% |  |  | -s10 | 97.66% | 69.17% |
| 10× | -s1 | 1.37% | 93.72% |  | 40× | -s1 | 0.39% | 98.28% |
|  | -s2 | 62.30% | 82.48% |  |  | -s2 | 12.19% | 97.30% |
|  | -s3 | 96.19% | 69.12% |  |  | -s3 | 63.49% | 96.55% |
|  | -s4 | 98.13% | 55.66% |  |  | -s4 | 88.83% | 95.37% |
|  | -s5 | 98.62% | 42.91% |  |  | -s5 | 94.18% | 94.05% |
|  | -s6 | 98.75% | 33.72% |  |  | -s6 | 94.90% | 92.70% |
|  | -s7 | 98.81% | 25.06% |  |  | -s7 | 95.79% | 91.12% |
|  | -s8 | 99.19% | 17.81% |  |  | -s8 | 96.40% | 88.89% |
|  | -s9 | 99.25% | 12.42% |  |  | -s9 | 96.72% | 86.24% |
|  | -s10 | 99.38% | 8.34% |  |  | -s10 | 96.88% | 83.22% |
| 20× | -s1 | 0.72% | 96.99% |  | 69× | -s1 | 0.25% | 98.54% |
|  | -s2 | 33.46% | 94.05% |  |  | -s2 | 4.96% | 97.93% |
|  | -s3 | 89.70% | 90.48% |  |  | -s3 | 28.47% | 97.62% |
|  | -s4 | 96.21% | 85.21% |  |  | -s4 | 63.80% | 97.33% |
|  | -s5 | 97.36% | 78.83% |  |  | -s5 | 82.12% | 96.88% |
|  | -s6 | 97.63% | 72.92% |  |  | -s6 | 83.79% | 96.51% |
|  | -s7 | 97.85% | 65.69% |  |  | -s7 | 88.21% | 96.18% |
|  | -s8 | 98.03% | 58.21% |  |  | -s8 | 91.53% | 95.57% |
|  | -s9 | 98.33% | 50.69% |  |  | -s9 | 93.79% | 94.91% |
|  | -s10 | 98.43% | 43.49% |  |  | -s10 | 94.78% | 94.09% |

The evaluation was performed by using *Truvari* available at https://github.com/spiralgenetics/truvari.

**Table S19. Benchmark results on the 40× NA19240 PacBio CLR dataset**

| **SV type** | **Tool** | **Precision** | **Recall** | **F-measure** | **Total calls** | **TP calls** |
| --- | --- | --- | --- | --- | --- | --- |
| Deletion | cuteSV | **70.77%** | 57.37% | **63.37%** | 9236 | 6536 |
|  | Sniffles | 65.86% | 56.07% | 60.57% | 9787 | 6446 |
|  | PBSV | 66.44% | 59.12% | 62.57% | 10367 | 6888 |
|  | SVIM | 61.45% | **62.54%** | 61.99% | 12113 | 7444 |
| Insertion & Duplication | cuteSV | **62.19%** | 51.77% | **56.50%** | 12378 | 7698 |
|  | Sniffles | 59.75% | 51.94% | 55.57% | 12928 | 7725 |
|  | PBSV | 56.59% | 48.93% | 52.48% | 13111 | 7420 |
|  | SVIM | 53.22% | **58.95%** | 55.94% | 17883 | 9517 |
| Inversion | cuteSV | 18.69% | 10.22% | **13.22%** | 107 | 20 |
|  | Sniffles | 4.11% | **13.33%** | 6.28% | 633 | 26 |
|  | PBSV | 27.78% | 7.11% | 11.32% | 54 | 15 |
|  | SVIM | **46.43%** | 6.67% | 11.66% | 28 | 13 |
| Total | cuteSV | **65.62%** | 54.19% | **59.36%** | 21721 | 14254 |
|  | Sniffles | 60.23% | 53.67% | 56.76% | 23348 | 14197 |
|  | PBSV | 60.87% | 53.54% | 56.97% | 23532 | 14323 |
|  | SVIM | 56.53% | **60.35%** | 58.38% | 30024 | 16974 |

The best results have been marked in bold respectively. The evaluation was performed by using *cmp_NA19240.py* available at <https://github.com/tjiangHIT/cuteSV>.

**Table S20. Nomenclature**

| **Abbreviations** | **Description** |
| --- | --- |
| ${base}_{cnt}$ | The connection of the breakend partners of a call in the truth sets |
| ${base}_{e}$ | The end coordinate of a call in the truth sets |
| ${base}_{s}$ | The starting coordinate of a call in the truth sets |
| ${base}_{L}$ | The length of a call in the truth sets |
| ${base}_{t}$ | The type of a call in the truth sets |
| ${Bias}_{L}$ | The bias of length for deletion and insertion |
| $BK$ | The coordinate at each breakpoint |
| $Chr$ | The unique chromosome ID |
| ${comp}_{cnt}$ | The connection of the breakend partners of the prediction |
| ${comp}_{e}$ | The end coordinate of the prediction |
| ${comp}_{s}$ | The starting coordinate of the prediction |
| ${comp}_{L}$ | The length of the prediction |
| ${comp}_{t}$ | The type of the prediction |
| ${Diff}_{dis}$ | The distance of two split alignments on the read and on the reference genome |
| ${Diff}_{olp}$ | The overlapping length of two split alignments on the reference genome |
| $\left\vert{Group}_{Sig} \right\vert$ | The number of the signatures in the signature cluster |
| ${INV}_{hh}$ | Head-to-head inversion |
| ${INV}_{tt}$ | Tail-to-tail inversion |
| ${Len}_{k}$ | The length of *k*-th longest signature in ${Group}_{Sig}$ |
| $MDR$ | Mendelian-Discordance-Rate |
| ${Read}_{e}$ | The end coordinate on the read |
| ${Read}_{ID}$ | The unique read ID |
| ${Read}_{s}$ | The starting coordinate on the read |
| ${Ref}_{e}$ | The end coordinate on the reference genome |
| ${Ref}_{s}$ | The starting coordinate on the reference genome |
| $Seg$ | The record of a split alignment |
| $Sig$ | The signature of an SV |
| ${Sig}_{BND}$ | The signature of a translocation |
| ${Sig}_{DEL}$ | The signature of a deletion |
| ${Sig}_{dis}$ | The distance threshold of two signatures |
| ${Sig}_{DUP}$ | The signature of a duplication |
| ${Sig}_{INS}$ | The signature of an insertion |
| ${Sig}_{INV}$ | The signature of an inversion |
| ${Sig}_{M}$ | The merged signature |
| $SR$ | The number of supporting reads for a specific cluster |
| ${SR}_{ALT}$ | The number of supporting reads for an alternative allele |
| ${SR}_{first}$ | The number of supporting reads for the largest sub-cluster |
| ${SR}_{min}$ | The threshold of the minimum number of supporting reads |
| ${SR}_{Ref}$ | The number of supporting reads for a reference allele |
| ${SR}_{second}$ | The number of supporting reads for the second largest sub-cluster |
| ${SV}_{L}$ | The SV length of the signature |
| ${TH}_{type}$ | The threshold of the distances among the clustered signatures for a specific SV type |
| $TN/FN$ | The number of true/false negative calls |
| $TP/FP$ | The number of true/false positive calls |
| $\alpha/\mu$ | The weighting parameters |
| $\mathcal{L}$ | Genotype likelihood |
| $\varepsilon$ | The probability of a read mapped to a given zygosity erroneously |

**Supplementary Notes**

**1. Commands used for read alignment and SV calling**

**1.1 Extraction of raw sequencing long reads**

samtools fasta {in.bam} > {raw_long_reads.fa}

**1.2 Read alignment**

**PBMM2 alignment**

**Index**

pbmm2 index {reference.fa} {reference.mmi} (optional: --preset CCS)

**Align**

pbmm2 align {reference.mmi} {raw_long_reads.fa} {raw_long_reads_pbmm2.bam} --sort --rg '@RG\tID:${SAMPLE}' --sample {SAMPLE} (optional: --preset CCS)

**NGMLR alignment**

ngmlr -r {reference} -q {raw_long_reads.fa} -o {raw_long_reads_ngmlr.sam} (optional: -x ont)

**Minimap2 alignment**

minimap2 {reference} {raw_long_reads.fq} -a -z 600,200 -x map-ont –MD -Y -o {raw_long_reads_minimap2.sam} -R ‘@RG\tID:${SAMPLE}’

**1.3 Post-processing of read alignment**

**Alignment sorting**

samtools view -buS {raw_long_reads_ngmlr.sam} | samtools sort -O bam -T {tempdir} - > {raw_long_reads_ngmlr.bam} && samtools index {raw_long_reads_ngmlr.bam}

**Alignment down-sampling**

samtools view -bS -s {ratio} {raw_long_reads.bam} > {raw_long_reads_subset.bam} && samtools index {raw_long_reads_subset.bam}

**1.4 SV calling**

**cuteSV calling**

**For PacBio CLR and ONT data**

python3 cuteSV.py {raw_long_reads.bam} {raw_long_reads_cuteSV.vcf} {workdir} -s {min_support} -l {min_svsize} –max_cluster_bias_INS 100 –diff_ratio_merging_INS 0.2 –diff_ratio_filtering_INS 0.6 –diff_ratio_filtering_DEL 0.7

**For PacBio HiFi data**

python3 cuteSV.py {raw_long_reads.bam} {raw_long_reads_cuteSV.vcf} {workdir} -s {min_support} -l {min_svsize} –max_cluster_bias_INS 200 –diff_ratio_merging_INS 0.65 –diff_ratio_filtering_INS 0.65 –diff_ratio_filtering_DEL 0.35

**Sniffles calling**

sniffles -s {min_support} -l {min_svsize} -m {raw_long_reads.bam} -v {raw_long_reads_sniffles.vcf} (optional: --skip_parameter_estimation)

**PBSV calling**

pbsv discover {raw_long_reads.bam} {raw_long_reads.svsig.gz}

pbsv call {reference.fa} {raw_long_reads.svsig.gz} {raw_long_reads_pbsv.vcf} (optional: --ccs, -t INS,DEL)

**SVIM calling**

svim alignment –min_sv_size 30 {workdir} {raw_long_reads.bam} {reference}

cat {workdir/final_results.vcf} | awk '{ if($1 ~ /^#/) { print $0 } else { if($5=="<DEL>" || $5=="<INS>") { print $0 }}}' | grep -v 'SUPPORT=1;\|SUPPORT=2;\|SUPPORT=3;\|SUPPORT=4;\|SUPPORT=5;\|SUPPORT=6;\|SUPPORT=7;\|SUPPORT=8;\|SUPPORT=9;' | sed 's/DUP:INT/INS/g' | sed 's/DUP:TANDEM/INS/g' > {raw_long_reads_svim.vcf}

or

cat {workdir/final_results.vcf} | awk '{ if($1 ~ /^#/) { print $0 } else { if($5=="<DEL>" || $5=="<INS>") { print $0 }}}' | grep -v 'SUPPORT=1;\|SUPPORT=2;\|SUPPORT=3;\|SUPPORT=4;' | sed 's/DUP:INT/INS/g' | sed 's/DUP:TANDEM/INS/g' > {raw_long_reads_svim.vcf}

**2. Truvari benchmarking**

bgzip {raw_long_reads.vcf} && tabix {raw_long_reads.vcf.gz}

truvari -f {reference.fa} -b {HG002_SVs_Tier1_v0.6.vcf.gz} --includebed {HG002_SVs_Tier1_v0.6.bed} -o {benchmarkdir} –passonly --giabreport -r 1000 -p 0.00 -c {raw_long_reads.vcf.gz}

**3. Simulated error-prone long-read alignments generation**

VISOR HACk -bed {answer.bed} -g {reference.fa} -o {HACKDIR}

VISOR LASeR -s {HACKDIR} -bed {bias.bed} -g {reference.fa} -o {ALIGNMENTDIR} -c {coverage} --noaddtag
