# Supplementary material for: Long-read-based human genomic structural variation detection with cuteSV
Source: Genome Biol. 2020 Aug 3;21:189. doi: 10.1186/s13059-020-02107-y (PMC7477834; doi:10.1186/s13059-020-02107-y)
Supplement: Supplementary file 2 — Additional file 2. Review history. [file 13059_2020_2107_MOESM2_ESM.docx]

Review History

**First round of review**

**Reviewer 1**

**Were you able to assess all statistics in the manuscript, including the appropriateness of statistical tests used?**

Yes: No statistical tests were used.

**Were you able to directly test the methods?**

Yes

**Comments to author:**

Tao Jiang et al. present a computational method, called cuteSV that discovers structural variants in long-read sequencing data. The study is focused on an important area of research because multiple recent publications (e.g., PMID: 31747936) have shown that only long-reads are able to discover the full spectrum of SVs, often unravelling >2-fold more SVs compared to short read sequencing efforts. Overall, the method builds upon established principles and leverages split-read signatures as well as insertions and deletions present in the CIGAR string to collect candidate SVs. These candidate SVs are then clustered by size, SV type and genomic location as in many other short-read and long-read SV callers. The method includes a number of additional heuristics that take into account the short-comings of mapping error-prone long reads such as the joining of adjacent insertions or deletions into longer SVs. The presented results appear robust and the method outperforms other mapping-based tools for SV detection. I have a few concerns pertaining to the method detailed below. The manuscript itself can be largely improved by a more succinct and accurate presentation of the tool.

Major comments

* Although mapping-based SV detection tools have been the method of choice for short reads, the long read community has a strong interest in haplotype-resolved assembly approaches for SV detection (e.g., PMID: 30992455, PMID: 27895111, or https://doi.org/10.1101/664623). One obvious benefit of these approaches is that they are free of mapping artifacts and that they inherently unravel the haplotype configuration of SVs. The manuscript should briefly review these assembly approaches and clarify the limitations of the proposed mapping-based strategy in the discussion section.

* In addition to the lack of providing haplotype-resolved SVs, it appears to me that the method or at least the genotyping is tailored for diploid organisms. Long reads have huge advantages for repetitive plant genomes but these are often non-diploid. The authors should clarify if cuteSV can be applied to arbitrary ploidy levels and what the limitations are in these cases.

* In many places the manuscript is unnecessary wordy and verbose. The Figures can be largely improved. Formulas should use a consistent variable naming and the authors should check them for accuracy. Examples:

(a) Figure 2 and 3 provide precision, recall and F1 scores on various data sets at different coverage levels. The numbers at the bar plots are beyond readability and a simple Table with the best method in bold could capture all this information. Colors are also re-used in the same Figure which is confusing (e.g., green for SVIM in 2C and green for GT-F1 in 2AB).

(b) Figure 5: x-axis labels are not readable.

(c) Discussion section: The text mainly reiterates previous results and lacks a succinct description of strength and weaknesses of the method.

(d) Formulas: The authors should introduce a clear nomenclature for variables and use these consistently. For instance, reference positions are labelled as 'pos' in Formula (1), as Ref_s/e in (2) and then as base_s/e in (13).

(e) Some abbreviations are simply confusing. For instance, Formula 6 uses '1>=' which apparently stands for an inter-chromosomal SV in +- orientation where the first chromosome is alphabetically larger than the second chromosome.

(f) The authors repeatedly use Mendelian-Discordance-Rates (MDRs) to evaluate methods. This is fine but the definition of MDR in Formula (18) has nothing to do with the classical definition of Mendelian inconsistent genotypes in a trio (mother, father, and child).

* The authors spend a considerable effort on evaluating cuteSV using GIAB long read data sets. Two shortcomings of the GIAB ground truth call set are (1) that it excludes some highly repetitive areas of the human genome that are known to be SV-rich (in order to identify false positives) and (2) that it is limited to insertions and deletions only. I would therefore suggest to complement the solid benchmarking on real data with a simulation benchmark that randomly implants known SVs of *all* SV types (e.g., from CHM1) in simulated reference genomes that are then used to draw error-prone long reads using a standard read simulator (e.g., PMID: 29982625 or PMID: 31589307).

Minor comments

* The output VCF of cuteSV lacks some useful information.

(a) A QUAL value would be beneficial to filter subsets of SVs at higher precision.

(b) Breakpoint uncertainties are usually expressed as CIPOS and CIEND instead of BREAKPOINT_STD. Please check the VCF specification if that's still the preferred notation.

(c) The method would gain in appeal by providing genotype qualities and likelihoods.

(d) For applications in cancer, inversions are usually classified as head-to-head and tail-to-tail inversions.

* For the runtime and memory benchmark (Figure 4) it wasn't clear to me if these results were computed with or without genotyping. The method appears to take significantly longer if genotyping is used, please include discovery-only and discovery+genotyping in the benchmark.

**Reviewer 2**

**Were you able to assess all statistics in the manuscript, including the appropriateness of statistical tests used?**

No

**Were you able to directly test the methods?**

Yes

**Comments to author:**

Paper introduces a structural variation discovery tool that uses long reads to call deletions, insertions, duplications, inversions and translocations.

- Authors claim cuteSV is a scalable tool. However, no experiment in the paper backs up this claim. While paper claims lower memory footprint and comparable runtimes with the state-of-the-art structural variation discovery methods, it does not assess these metrics with respect to datasets of different sizes.

R- untime analysis shows Sniffles is the fastest when tools are run on a single core and authors claim cuteSV speeds up linearly with the number of threads and it is faster than Sniffles when more than 4 threads are used. Authors claim the number of threads used by Sniffles and PBSV doesn't improve runtime. Performance of Sniffles and PBSV with many cores should be shown in the figure 4.

- Usually the precision, recall and F1 measures are calculated through simulated data that carry the artifacts of instruments. In this manuscript, the precision and recall are calculated based on real datasets. This should be properly done through use of a simulated dataset.

- Following the previous point, the authors should still report on the real datasets precision and recall. One of the more important questions is what is the basis for ground through for NA19240, they have used the validation data from a previous study but for HG002, it is not clear how the ground truth has been obtained.

- Inversions are one of the more challenging SV types to discover, it is not clear why they are removed from the evaluation of HG002.

- Paper should explain what types of calls cuteSV can capture while other methods fail. It is not clear if the precision/recall improvement is due to method or the events not being targeted by the competitor tools.

- Paper should emphasize the novel ideas employed in cuteSV that lead to improved accuracy/recall.

- While low memory requirements of cuteSV is impressive, most analysis will be bottle-necked by the read alignment step. If trade off between memory usage and speed is possible, having an option to move disk usage to memory would improve the tool for users who have available memory.

- In the supplementary figure 4, the difference between cuteSV and other methods seems to be marginal.

- In equation 13 2nd condition seems to have a typo.

**Authors Response**

**Point-by-point responses to the reviewers’ comments:**

Reviewer: 1

Comments to the Author

Tao Jiang et al. present a computational method, called cuteSV that discovers structural variants in long-read sequencing data. The study is focused on an important area of research because multiple recent publications (e.g., PMID: 31747936) have shown that only long-reads are able to discover the full spectrum of SVs, often unravelling >2-fold more SVs compared to short read sequencing efforts. Overall, the method builds upon established principles and leverages split-read signatures as well as insertions and deletions present in the CIGAR string to collect candidate SVs. These candidate SVs are then clustered by size, SV type and genomic location as in many other short-read and long-read SV callers. The method includes a number of additional heuristics that take into account the short-comings of mapping error-prone long reads such as the joining of adjacent insertions or deletions into longer SVs. The presented results appear robust and the method outperforms other mapping-based tools for SV detection. I have a few concerns pertaining to the method detailed below. The manuscript itself can be largely improved by a more succinct and accurate presentation of the tool.

Major comments

* Although mapping-based SV detection tools have been the method of choice for short reads, the long read community has a strong interest in haplotype-resolved assembly approaches for SV detection (e.g., PMID: 30992455, PMID: 27895111, or https://doi.org/10.1101/664623). One obvious benefit of these approaches is that they are free of mapping artifacts and that they inherently unravel the haplotype configuration of SVs. The manuscript should briefly review these assembly approaches and clarify the limitations of the proposed mapping-based strategy in the discussion section.

*Response:*

*Thank you very much for reviewing our manuscript. Yes, the assembly-based approaches have their own advantages to the detection of SV, especially they are free of mapping artifacts. And they could be also complementary to alignment-based approaches. We have added a brief review and discussion in Background and Discussion sections.*

*We have mentioned in the current version of the manuscript:*

*"De novo assembly-based approaches [27-29] aim at assembling reads to longer genomic sequences (i.e., contigs and/or scaffolds) and discover SVs from the alignments between the assembled sequences and a reference. Such approaches are less influenced by the reference than that of read alignment-based approaches, especially free of read alignment artifacts. However, they are usually computational-intensive and still have some difficulties in the reconstruction of haplotype sequences of large genomes [30], which are also shortcomings to SV calling (a brief discussion is in Discussion section)." (line 27-34, page 2 of Background section)*

*"Moreover, from a higher-level view, de novo assembly-based approaches have their own advantages to cuteSV as well as other alignment-based SV callers. Firstly, although the mappability of long reads is much higher than that of short reads, their alignments are still heterogenous and have potential errors due to many factors, such as sequencing errors, SV complexity, repetitive sequences, etc. However, de novo assembly-based approaches are free of such alignment artifacts so that they could have less systematic errors. Secondly, de novo assembly-based approaches are able to well-handle large novel insertions by assembling them as distinctive contigs, however, this type of SV is still non-trivial to alignment-based approaches. Thirdly, haplotype-resolved assembly inherently helps to unravel the haplotype configuration of SVs, which is useful to many downstream analyses.*

*However, de novo assembly-based approaches also have some non-neglectable bottlenecks. Firstly, the assembly could be not complete, i.e., some of the haplotype sequences (especially contigs of SV alleles) are missing, which may affect the sensitivity of SV calling. Secondly, assembly mistakes still exist, which may cause false positives. Thirdly, de novo assembly is still computational-intensive, which is not cost-effective for large-scale data analysis tasks. Fourthly, it usually needs high-coverage sequencing or the combination of multiple types of data (such as short reads, long reads, Hi-C and optical map) to implement haplotype-resolved assembly [51], however, most of SVs can be detected with lower coverage data by alignment-based approaches like cuteSV. Considering their own advantages and shortcomings, we consider that alignment- and assembly-based approaches are complementary to each other, and it could be useful to integrate these two approaches to produce SV callsets with higher quality." (line 27-52, page 9 of Discussion section)*

* In addition to the lack of providing haplotype-resolved SVs, it appears to me that the method or at least the genotyping is tailored for diploid organisms. Long reads have huge advantages for repetitive plant genomes but these are often non-diploid. The authors should clarify if cuteSV can be applied to arbitrary ploidy levels and what the limitations are in these cases.

*Response:*

*Thank you very much for pointing this out. The current version of cuteSV is tailored for diploid genomes. For multi-ploidy genomes such as plant genomes, cuteSV can also extract the signatures to detect SVs. However, genotyping is still implemented with the diploid model. In this situation, cuteSV could have decreased yields since there could be more heterozygous SVs and less SV supporting reads for specific SV alleles, and the genotypes of SVs are more complicated. We have added a paragraph in Discussion section to clarify this issue. And it is also very important future works for us to develop more sensitive SV signature extraction methods and specifically designed genotyping models to handle the datasets from multi-ploidy genomes such as plant genomes.*

*We have mentioned in the current version of the manuscript:*

*"2) The current version of cuteSV is tailored for human genomes or diploid genomes. For multi-ploidy genomes such as many plant genomes, cuteSV can also extract the signatures to detect SVs, however, genotyping is still implemented with the diploid model. Such tasks are still non-trivial to cuteSV (as well as other state-of-the-art SV calling approaches), since there could be more heterozygous SVs and less SV supporting reads (as more alleles exist), and the genotypes of SVs are also more complicated as well. With the current implementation, cuteSV could miss some SV alleles in multi-ploidy genomes due to low numbers of supporting reads, meanwhile, it may make mistakes in genotyping. This is an important work for us to develop more sensitive SV signature extraction methods as well as specifically designed genotyping model to the SV detection of multi-ploidy genomes." (line 8-19, page 9 of Discussion section)*

* In many places the manuscript is unnecessary wordy and verbose. The Figures can be largely improved. Formulas should use a consistent variable naming and the authors should check them for accuracy. Examples:

*Response:*

*Thank you very much for reading the manuscript in detail and giving our helpful suggestions. To improve the quality of writing, we have revised some paragraphs, improved the quality of the figures and used consistent and accurate variable naming, according to your comments.*

(a) Figure 2 and 3 provide precision, recall and F1 scores on various data sets at different coverage levels. The numbers at the bar plots are beyond readability and a simple Table with the best method in bold could capture all this information. Colors are also re-used in the same Figure which is confusing (e.g., green for SVIM in 2C and green for GT-F1 in 2AB).

*Response:*

*Thank you very much for pointing this out. We have re-plotted these two figures. Radar charts are used to show F1 scores of SV callers on various HG002 sequencing datasets at different coverages. The detailed F1 scores as well as precision and recall rates are shown in Supplementary Tables 7, 8 and 10, and the best results have been marked bold as well. Meanwhile, we have used consistent coloring in the figures to avoid color misleading.*

(b) Figure 5: x-axis labels are not readable.

*Response:*

*We have adjusted the resolution of the figure to show all the elements clearly. It is also worthnoting that, in the current version of manuscript, this figure has been moved to Supplementary Figure 3.*

(c) Discussion section: The text mainly reiterates previous results and lacks a succinct description of strength and weaknesses of the method.

*Response:*

*Thank you very much for the suggestion. We have revised the Discussion section carefully to describe both of the strength and weaknesses of cuteSV. A brief discussion on the assembly-based SV detection approach is also added.*

*We have mentioned in the current version of the manuscript:*

*"Long-read sequencing technologies are promising to discover the SVs of sequenced sample comprehensively. However, it is still non-trivial to exert the advantages of long reads due to the high sequencing errors and the complexity of SVs. Herein, we propose cuteSV, a novel read alignment-based SVs calling approach, to show how to achieve higher yields, speed and scaling performance in SV calling with tailored signature extraction, clustering and refinement methods.*

*Mainly, the proposed approach has four advantages as follows.*

*1) The heuristic SV signature extraction methods enable to well-handle the fragile read alignments around SV breakpoints, which helps to build high quality SV evidence. This is one of the major reasons that cuteSV outperforms state-of-the-art tools. An example is in Supplementary Figure 6. It shows how a 272bp insertion event was successfully discovered by cuteSV, while other benchmarked SV callers were affected by the multiple insertions and deletions in read alignments.*

*2) The clustering-and-refinement method enables to distinguish the reads from the multiple SV alleles in the same loci and generate correct signature clusters. Thus, complex heterozygous SVs can be detected and genotyped sensitively and accurately, which also outperforms state-of-the-art tools. Two examples are shown in Supplementary Figure 7. One is a heterozygous insertion event (a 108bp and a 36bp insertion in the same region) and the other is a heterozygous deletion event (a 123bp and a 37 bp deletion in the same region). Only cuteSV and PBSV successfully discovered them.*

*3) With its novel heuristic methods, cuteSV improves the sensitivity of SV calling especially for lower coverage datasets. The benchmark results suggest that cuteSV can discover most of the SVs in 20× coverage datasets for human samples without loss of accuracy, meanwhile, the performance of genotyping is consistent with its yields as well. This is helpful to make more flexible and cost-effective sequencing plans in large-scale genomics studies.*

*4) With its block division-based implementation, cuteSV has outstanding scaling performance, which enables to achieve a nearly linear multiple-thread speedup during data processing. This is very suited to modern HPC resources and helpful to for large-scale genomics studies.*

*However, from the view of alignment-based SV calling, there are still a few shortcomings for cuteSV as following, which are also important future works to further improve the approach.*

*1) There are still some SVs that cannot be successfully detected by cuteSV. We investigated the intermediate results of cuteSV and found that most of the false negative calls were due to the read alignments being inaccurate or not informative enough. Two typical examples are shown in Supplementary Figures 8 and 9. In Supplementary Figure 8, a 98bp deletion case is shown in which deletion signatures emerge in nearly all the reads around the event. However, the size of the deletions in the alignment are not correct (i.e., most of them are around 50bp). In Supplementary Figure 9, a 707bp insertion case is shown in which insertion signatures also emerge and their sizes are close to the SV event, but the positions of the breakpoints in the reads are quite far from the ground truth breakpoint. Under such circumstances, all the benchmarked SV callers made SV calls, but the sizes and/or positions are incorrect since the read alignments are misleading.*

*2) The current version of cuteSV is tailored for human genomes or diploid genomes. For multi-ploidy genomes such as many plant genomes, cuteSV can also extract the signatures to detect SVs, however, genotyping is still implemented with the diploid model. Such tasks are still non-trivial to cuteSV (as well as other state-of-the-art SV calling approaches), since there could be more heterozygous SVs and less SV supporting reads (as more alleles exist), and the genotypes of SVs are also more complicated as well. With the current implementation, cuteSV could miss some SV alleles in multi-ploidy genomes due to low numbers of supporting reads, meanwhile, it may make mistakes in genotyping. This is an important work for us to develop more sensitive SV signature extraction methods as well as specifically designed genotyping model to the SV detection of multi-ploidy genomes.*

*3) Although it has higher scaling performance, the speed of cuteSV in single thread is still slower than some of state-of-the-art approaches. This is mainly due to the SV signature extraction of cuteSV is more complicated. An optimization on the data structures and operations of cuteSV could be feasible to further reduce the time cost.*

*Moreover, from a higher-level view, de novo assembly-based approaches have their own advantages to cuteSV as well as other alignment-based SV callers. Firstly, although the mappability of long reads is much higher than that of short reads, their alignments are still heterogenous and have potential errors due to many factors, such as sequencing errors, SV complexity, repetitive sequences, etc. However, de novo assembly-based approaches are free of such alignment artifacts so that they could have less systematic errors. Secondly, de novo assembly-based approaches are able to well-handle large novel insertions by assembling them as distinctive contigs, however, this type of SV is still non-trivial to alignment-based approaches. Thirdly, haplotype-resolved assembly inherently helps to unravel the haplotype configuration of SVs, which is useful to many downstream analyses.*

*However, de novo assembly-based approaches also have some non-neglectable bottlenecks. Firstly, the assembly could be not complete, i.e., some of the haplotype sequences (especially contigs of SV alleles) are missing, which may affect the sensitivity of SV calling. Secondly, assembly mistakes still exist, which may cause false positives. Thirdly, de novo assembly is still computational-intensive, which is not cost-effective for large-scale data analysis tasks. Fourthly, it usually needs high-coverage sequencing or the combination of multiple types of data (such as short reads, long reads, Hi-C and optical map) to implement haplotype-resolved assembly [51], however, most of SVs can be detected with lower coverage data by alignment-based approaches like cuteSV. Considering their own advantages and shortcomings, we consider that alignment- and assembly-based approaches are complementary to each other, and it could be useful to integrate these two approaches to produce SV callsets with higher quality." (line 11-59, page 8 and line 1-52, page 9 of Discussion section)*

(d) Formulas: The authors should introduce a clear nomenclature for variables and use these consistently. For instance, reference positions are labelled as 'pos' in Formula (1), as Ref_s/e in (2) and then as base_s/e in (13).

*Response:*

*Thank you very much for this helpful suggestion. We have added a nomenclature in Supplementary Table 17 to improve the readability. Moreover, consistent variable naming has been used in all the equations.*

*We have mentioned in the current version of the manuscript:*

*“Some details on the implementations of cuteSV approach and the benchmark are as following. Please also refer to Supplementary Table 17 for a nomenclature.” (line 9-10, page 10 of Materials and methods section)*

(e) Some abbreviations are simply confusing. For instance, Formula 6 uses '1>=' which apparently stands for an inter-chromosomal SV in +- orientation where the first chromosome is alphabetically larger than the second chromosome.

*Response:*

*We have carefully checked and corrected all the abbreviations in the equations. Eq. (6) has been revised as follows.*

*"*

*(Chr_1, Ref_1e, Chr_2, Ref_2s, Read_ID), if Chr_1 < Chr_2 and ++*

*(Chr_2, Ref_2s, Chr_1, Ref_1e, Read_ID), if Chr_2 < Chr_1 and ++*

*(Chr_1, Ref_1e, Chr_2, Ref_2e, Read_ID), if Chr_1 < Chr_2 and +-*

*SIG_BND = (Chr_2, Ref_2e, Chr_1, Ref_1e, Read_ID), if Chr_2 < Chr_1 and +- (6)*

*(Chr_1, Ref_1s, Chr_2, Ref_2s, Read_ID), if Chr_1 < Chr_2 and -+*

*(Chr_2, Ref_2s, Chr_1, Ref_1s, Read_ID), if Chr_2 < Chr_1 and -+*

*(Chr_1, Ref_1s, Chr_2, Ref_2e, Read_ID), if Chr_1 < Chr_2 and --*

*(Chr_2, Ref_2e, Chr_1, Ref_1s, Read_ID), if Chr_2 < Chr_1 and --*

*where < means the first chromosome is alphabetically smaller than the second chromosome and “++”, “+-”, “-+”, “--” indicate the combination of strands for an inter-chromosomal SV." (line 33-46, page 11 of Materials and methods section)*

(f) The authors repeatedly use Mendelian-Discordance-Rates (MDRs) to evaluate methods. This is fine but the definition of MDR in Formula (18) has nothing to do with the classical definition of Mendelian inconsistent genotypes in a trio (mother, father, and child).

*Response:*

*We have carefully referred to the assessment of trio data in Sniffles (PMID: 29713083) and largely improved our corresponding benchmarking by the following methods:*

*1) We added the recall rate (Formula 19), i.e., the percentage of homozygous parental variants found in offspring;*

*2) We corrected the definition of MDR (Formula 20), which indicates the percentage of SV calls unique to the offspring.*

*The new results are in “SV detection with HG002 PacBio data” of Results section, Figures 3C, 3D and Supplementary Table 9.*

*We have mentioned in the current version of the manuscript:*

*"To assess the ability to the detection of various types of SVs (i.e., insertions, deletions, inversions, duplications and translocations) more comprehensively, we further employed GIAB Ashkenazi Trio PacBio CLR datasets (HG002, HG003 and HG004) to assess the recall rates and Mendelian-Discordance-Rates (MDRs). cuteSV and SVIM obtained >95% mean recall rate, i.e., more than 95% homozygous parental SVs has been confirmed in the offspring (Figure 3C and Supplementary Table 9). cuteSV was 1% lower than SVIM on recall rate, however, we realized that this does not mean a lower sensitivity of cuteSV, but due to that about 15% SVs in parental callsets discovered by SVIM had no genotypes so that they cannot be assessed and decreased the total number of homozygous parental SVs. Meanwhile, the MDR of cuteSV is lowest (7.36%, Figure 3D and Supplementary Table 9), indicating that its callsets were more plausible. The MDRs of SVIM (7.62%) and PBSV (9.25%) are also comparable, and much lower than that of Sniffles (17.45%)." (line 41-55, page 5 of Results section)*

*"For the evaluation of the Ashkenazi trio, we used the homozygous SV calls in parents (i.e., HG003 and HG004) to measure the recall rate via Eq.13-15 as follows:*

*Recall_trio = (∑ SVs in the offspring) / (∑ homozygous SVs in parents) (19)*

*Then, we applied all parental SV calls as ground truth to assess the percentage of homozygous and heterozygous SV calls in the son (i.e., HG002) cannot be detected in its parents. Therefore, the ratio of MDR can be reckoned via:*

*MDR = (∑ offspring's SVs not reidentified in parents) / (∑ offspring's SVs) (20)*

*" (line 42-54, page 16 of Materials and methods section)*

* The authors spend a considerable effort on evaluating cuteSV using GIAB long read data sets. Two shortcomings of the GIAB ground truth call set are (1) that it excludes some highly repetitive areas of the human genome that are known to be SV-rich (in order to identify false positives) and (2) that it is limited to insertions and deletions only. I would therefore suggest to complement the solid benchmarking on real data with a simulation benchmark that randomly implants known SVs of *all* SV types (e.g., from CHM1) in simulated reference genomes that are then used to draw error-prone long reads using a standard read simulator (e.g., PMID: 29982625 or PMID: 31589307).

*Response:*

*Thank you for this helpful suggestion. Yes, the GIAB ground truth has its own limitations. We have added 20 PacBio-like simulated datasets with various types of SVs and various sequencing coverages to implement a simulation benchmark.*

*In details, we collected 20202 various types of known non-overlapping SVs, i.e., 6167, 9899 and 44 deletions, insertions and inversions from CHM1 sample callsets (nstd137 in dbVAR database), and 3712 and 380 non-overlapping duplications and translocations from KWS1 sample callsets (nstd106 in dbVAR database) were also extracted, respectively. The various types of SVs were separately input to VISOR with human reference genome (version: hs37d5) to generate five in silico donor genomes. And four PacBio-like datasets were simulated for each of the donor genomes (mean read length: 8000 bp; error model: default setting of PBSIM simulator; coverages: 5×, 10×, 20× and 30×, respectively), i.e., 20 datasets were simulated in total. cuteSV, Sniffles, PBSV and SVIM, were implemented on the simulated datasets for comparison. The trend of the SV detection results is similar to that of real sequencing datasets, that cuteSV has highest F1 scores on almost all the datasets. The results are given in the “SV detection with simulated data” of Results section, and the implementation details on the benchmark is given in “Implementation of simulation” of Material and methods section.*

*We have mentioned in “SV detection with simulated data” of Results section:*

*"We used simulated datasets in various sequencing coverages to assess the “baseline” sensitivity and accuracy of cuteSV. More precisely, we collected 20202 various types of known non-overlapping SVs, i.e., 6167, 9899 and 44 deletions, insertions and inversions from CHM1 sample callsets [41] (nstd137 in dbVAR database), and 3712 and 380 duplications and translocations from KWS1 sample callsets [42] (nstd106 in dbVAR database), respectively. The various types of SVs were separately input to VISOR [43] with human reference genome (version: hs37d5) to generate five in silico donor genomes. And four PacBio-like datasets were simulated for each of the donor genomes (mean read length: 8000 bp; error model: default setting of PBSIM simulator [44]; coverages: 5×, 10×, 20× and 30×, respectively), thus, 20 datasets were simulated in total. cuteSV and three state-of-the-art SV callers, i.e., Sniffles, PBSV and SVIM, were implemented on the simulated datasets for comparison. Refer to Materials and methods Section for more details on the implementation of the simulation.*

*The benchmark results (Figure 2A, 2B, 2C and Supplementary Table 1, 2, 3) indicate that cuteSV achieved highest F1 scores on the detection of deletions, insertions and duplications for almost all the coverages w/o genotyping. It also had highest F1 scores for discovering inversions (Figure 2D and Supplementary Table 4), but the best runner-up for genotyping due to slightly lower precision. For translocations, cuteSV and Sniffles were the best two callers at breakpoint level (Figure 2E and Supplementary Table 5), meanwhile, cuteSV and PBSV showed higher F1 scores at breakend level (Figure 2F and Supplementary Table 6) (refer to Materials and methods Section for the method of breakpoint- and breakend-level assessment for translocations). It is worth noting that SVIM did not assign genotypes for all the translocations and most of the duplications, which resulted in lower statistics. Overall, the results suggest that cuteSV is a good versatile SV caller, i.e., it is able to sensitively detect various types of SVs without lack of accuracy, and their genotypes can be correctly recognized as well." (line 24-54, page 4 of Results section)*

*We also mentioned in “Implementation of simulation” of Materials and methods section:*

*"6167, 9899 and 44 deletions, insertions and inversions were extracted from CHM1 sample callsets (nstd137 in dbVAR database). The extracted SVs are non-overlapping and meet the condition that experiment IDs are 1 or 2 and all of ACs, ANs and AFs are higher than 0. Similarly, 3712 and 380 non-overlapping duplications and translocations were extracted from KWS1 sample callsets (nstd106 in dbVAR database), respectively. Then, the five types of SVs were respectively integrated into human reference genome (hs37d5) to build five in silico donor genomes to generate simulated datasets with VISOR simulator. For a specific type of SVs, the simulation is done by the following two steps.*

*(1) The SVs were transformed into BED format according to the requirement of VISOR HACk. Deletions, insertions or inversions were directly spiked into the reference to generate haplotype in silico donor genomes correspondingly. Employed translocations and duplications were preprocessed as reciprocal translocations and tandem duplications with CN=2 before input to VISOR, respectively. It is also worth noting that, translocations in same strand orientations (e.g. forward-forward and reverse-reverse reciprocal translocations) have 2 kinds of breakpoint combinations and 4 kinds of breakend combinations, and translocations in different strand orientations (e.g. forward-reverse and reverse-forward reciprocal translocations) have 4 kinds of breakpoint combinations and 4 kinds of breakend combinations. A schematic illustration is in Supplementary Figure 11.*

*(2) For a given donor genome, its in silico chromosomes were randomly selected as “homozygous” and “heterozygous” chromosomes to mimic homozygous and heterozygous SVs. For a “homozygous” chromosome, all the reads were simulated from the in silico chromosome with SVs. For a “heterozygous” chromosome, 50% of the reads were simulated from the in silico chromosome and 50% from the corresponding reference chromosome. With this rule, four PacBio-like datasets in various coverages (5×, 10×, 20× and 30×) were produced by VISOR LASeR (using PBSIM and minimap2 with default setting). Thus, there are 20 datasets produced for the five donor genomes and the specific sets of SVs and their genotypes were used as ground truth.*

*The used command lines for data simulation are in Supplementary Notes." (line 9-44, page 14 of Materials and methods section)*

Minor comments

* The output VCF of cuteSV lacks some useful information.

(a) A QUAL value would be beneficial to filter subsets of SVs at higher precision.

*Response:*

*In the current version of SV callsets generated from cuteSV v1.0.6, each SV call contains variant quality score (i.e. QUAL), which is calculated via phred-scaled genotype likelihood.*

*We have mentioned in the current version of the manuscript:*

*"After the calculation of the original likelihoods, cuteSV normalizes these values by computing the log sum of exponentials. The final genotype is determined by the zygosity with the maximum genotype likelihood. After genotyping, cuteSV produces the phred-scaled genotype likelihoods, conditional genotype quality and phred-scaled quality score of SV in order to further quality control and higher accuracy callsets generation." (line 58-59, page 13 and line 1-5, page 14 of Materials and methods section)*

(b) Breakpoint uncertainties are usually expressed as CIPOS and CIEND instead of BREAKPOINT_STD. Please check the VCF specification if that's still the preferred notation.

*Response:*

*The new version of cuteSV discards the BREAKPOINT_STD values and adds CIPOS and CILEN values for deletions and insertions. This modification is consistent with the VCFv4.2 format specification and more suitable for describing the uncertainty.*

(c) The method would gain in appeal by providing genotype qualities and likelihoods.

*Response:*

*In the current version of SV callsets generated from cuteSV v1.0.6, each SV call contains phred-scaled genotype likelihood (i.e. PL) and genotype quality score (i.e. GQ). We have mentioned in the current version of the manuscript:*

*"After the calculation of the original likelihoods, cuteSV normalizes these values by computing the log sum of exponentials. The final genotype is determined by the zygosity with the maximum genotype likelihood. After genotyping, cuteSV produces the phred-scaled genotype likelihoods, conditional genotype quality and phred-scaled quality score of SV in order to further quality control and higher accuracy callsets generation." (line 58-59, page 13 and line 1-5, page 14 of Materials and methods section)*

(d) For applications in cancer, inversions are usually classified as head-to-head and tail-to-tail inversions.

*Response:*

*The new released cuteSV supports to the detection of head-to-head and tail-to-tail inversions, which are marked as “++” and “--” in the STRANDS info, respectively.*

* For the runtime and memory benchmark (Figure 4) it wasn't clear to me if these results were computed with or without genotyping. The method appears to take significantly longer if genotyping is used, please include discovery-only and discovery+genotyping in the benchmark.

*Response:*

*Yes, the genotyping step of cuteSV is to some extent time-consuming (used 17% to 55% of the total runtime on the various datasets in benchmarking), since it needs to re-check all the reads around the detected SV breakpoints to infer SV genotypes. We have added a thorough assessment on runtime and memory footprint by using 69x PacBio CLR, 28x PacBio CCS, and 47x ONT PromethION datasets (also according to the comment of Reviewer #2). For cuteSV, Sniffles and SVIM, both with and without genotyping were assessed. PBSV was only assessed with all the steps, as it does not have an option to skip genotyping. The results are given in “The performance of the SV callers” of Results section, Figure 5 and Supplementary Table 12.*

*We have mentioned in the section “the performance of the SV callers”:*

*"We assessed the speed and memory footprint of the SV callers on the three HG002 datasets (Figure 5 and Supplementary Table 12). Generally, using a single CPU thread, Sniffles with genotyping was faster than other benchmarked SV callers, i.e., Sniffles (without genotyping), SVIM (w/o genotyping), cuteSV (w/o genotyping) and PBSV (genotyping cannot be skipped). It is also worthnoting that the genotyping step of cuteSV costs 17% to 55% of the time on various datasets, since it needs to re-check all the reads around the detected SV breakpoints to infer SV genotypes. When using multiple CPU threads, only cuteSV has high scaling performance, i.e., it achieved a quasi linear speedup with the number of CPU threads and its wall clock time was greatly reduced. Neither Sniffles nor PBSV had an obvious speedup with more CPU threads while SVIM does not support multiple-thread computing. In this situation, cuteSV is faster than all the other callers when using 4 or more threads. On memory footprint, cuteSV (about 0.1GB to 0.4GB) was smaller than that of other approaches by one or two orders of magnitude. With its quasi linear multiple-threads speedup and low memory footprint, we realized that cuteSV is a highly scalable SV detection tool, which is suited to high performance computing platforms and large-scale data analysis tasks such as SV detection in many samples." (line 37-55, page 6 of Results section)*

---------------------------------------------------

Reviewer: 2

Paper introduces a structural variation discovery tool that uses long reads to call deletions, insertions, duplications, inversions and translocations.

- Authors claim cuteSV is a scalable tool. However, no experiment in the paper backs up this claim. While paper claims lower memory footprint and comparable runtimes with the state-of-the-art structural variation discovery methods, it does not assess these metrics with respect to datasets of different sizes.

*Response:*

*Thank you very much for pointing this out. cuteSV has good scaling performance, as it achieves a quasi linear speedup with the number of CPU threads, while other benchmarked SV callers do not have obvious speedup (i.e., PBSV and Sniffles) or does not support multiple thread computing (i.e., SVIM). It could be not clear to describe this point as only the runtime with single CPU thread of the SV callers other than cuteSV was given in Figure 4 of the original version of the manuscript.*

*In the current version of the manuscript, we added new assessments on three datasets, i.e., HG002 PacBio CLR 69x dataset, HG002 PacBio CCS 28x dataset and the HG002 ONT PromethION 47x dataset to backup this issue. All the runtimes of the SV callers with 1, 2, 4, 8 and 16 threads are given (except for SVIM as it does not support multiple thread). The figure (Figure 5 for the current version) has been completely updated, and the scaling performance of cuteSV is more obvious.*

*We also supply the memory footprints of the SV callers on the three datasets (also with various numbers of CPU threads). The three datasets are in different sizes (i.e., 69x, 28x and 47x, respectively), and cuteSV shows lower memory footprints on all of them.*

*We have mentioned in the current version of the manuscript:*

*"We assessed the speed and memory footprint of the SV callers on the three HG002 datasets (Figure 5 and Supplementary Table 12). Generally, using a single CPU thread, Sniffles with genotyping was faster than other benchmarked SV callers, i.e., Sniffles (without genotyping), SVIM (w/o genotyping), cuteSV (w/o genotyping) and PBSV (genotyping cannot be skipped). It is also worthnoting that the genotyping step of cuteSV costs 17% to 55% of the time on various datasets, since it needs to re-check all the reads around the detected SV breakpoints to infer SV genotypes. When using multiple CPU threads, only cuteSV has high scaling performance, i.e., it achieved a quasi linear speedup with the number of CPU threads and its wall clock time was greatly reduced. Neither Sniffles nor PBSV had an obvious speedup with more CPU threads while SVIM does not support multiple-thread computing. In this situation, cuteSV is faster than all the other callers when using 4 or more threads. On memory footprint, cuteSV (about 0.1GB to 0.4GB) was smaller than that of other approaches by one or two orders of magnitude. With its quasi linear multiple-threads speedup and low memory footprint, we realized that cuteSV is a highly scalable SV detection tool, which is suited to high performance computing platforms and large-scale data analysis tasks such as SV detection in many samples." (line 37-55, page 6 of Results section)*

- Runtime analysis shows Sniffles is the fastest when tools are run on a single core and authors claim cuteSV speeds up linearly with the number of threads and it is faster than Sniffles when more than 4 threads are used. Authors claim the number of threads used by Sniffles and PBSV doesn't improve runtime. Performance of Sniffles and PBSV with many cores should be shown in the figure 4.

*Response:*

*According to your suggestion, we have supplied the performance of Sniffles and PBSV with 1, 2, 4, 8 and 16 CPU threads in Figure 5.*

*We have mentioned in the current version of the manuscript:*

*“We assessed the speed and memory footprint of the SV callers on the three HG002 datasets (Figure 5 and Supplementary Table 12). Generally, using a single CPU thread, Sniffles with genotyping was faster than other benchmarked SV callers, i.e., Sniffles (without genotyping), SVIM (w/o genotyping), cuteSV (w/o genotyping) and PBSV (genotyping cannot be skipped).” (line 37-41, page 6 of Results section)*

- Usually the precision, recall and F1 measures are calculated through simulated data that carry the artifacts of instruments. In this manuscript, the precision and recall are calculated based on real datasets. This should be properly done through use of a simulated dataset.

*Response:*

*Thank you very much for the helpful suggestion. We have added 20 PacBio-like simulated datasets with various types of SVs and various sequencing coverages to implement a simulation benchmark.*

*Specifically, we collected 20202 various types of known non-overlapping SVs, i.e., 6167, 9899 and 44 deletions, insertions and inversions from CHM1 sample callsets (nstd137 in dbVAR database), and 3712 and 380 non-overlapping duplications and translocations from KWS1 sample callsets (nstd106 in dbVAR database) also collected, respectively. The various types of SVs were separately input to VISOR with human reference genome (version: hs37d5) to generate five in silico donor genomes. And four PacBio-like datasets were simulated for each of the donor genomes (mean read length: 8000 bp; error model: default setting of PBSIM simulator; coverages: 5×, 10×, 20× and 30×, respectively), i.e., 20 datasets were simulated in total. cuteSV, Sniffles, PBSV and SVIM, were implemented on the simulated datasets for comparison. The trend of the SV detection results is similar to that of real sequencing datasets, that cuteSV has highest F1 scores on almost all the datasets. The results are given in the “SV detection with simulated data” of Results section, and the implementation details on the benchmark is given in “Implementation of simulation” of Material and methods section.*

*We have mentioned in the current version of the manuscript:*

*"We used simulated datasets in various sequencing coverages to assess the “baseline” sensitivity and accuracy of cuteSV. More precisely, we collected 20202 various types of known non-overlapping SVs, i.e., 6167, 9899 and 44 deletions, insertions and inversions from CHM1 sample callsets [41] (nstd137 in dbVAR database), and 3712 and 380 duplications and translocations from KWS1 sample callsets [42] (nstd106 in dbVAR database), respectively. The various types of SVs were separately input to VISOR [43] with human reference genome (version: hs37d5) to generate five in silico donor genomes. And four PacBio-like datasets were simulated for each of the donor genomes (mean read length: 8000 bp; error model: default setting of PBSIM simulator [44]; coverages: 5×, 10×, 20× and 30×, respectively), thus, 20 datasets were simulated in total. cuteSV and three state-of-the-art SV callers, i.e., Sniffles, PBSV and SVIM, were implemented on the simulated datasets for comparison. Refer to Materials and methods Section for more details on the implementation of the simulation.*

*The benchmark results (Figure 2A, 2B, 2C and Supplementary Table 1, 2, 3) indicate that cuteSV achieved highest F1 scores on the detection of deletions, insertions and duplications for almost all the coverages w/o genotyping. It also had highest F1 scores for discovering inversions (Figure 2D and Supplementary Table 4), but the best runner-up for genotyping due to slightly lower precision. For translocations, cuteSV and Sniffles were the best two callers at breakpoint level (Figure 2E and Supplementary Table 5), meanwhile, cuteSV and PBSV showed higher F1 scores at breakend level (Figure 2F and Supplementary Table 6) (refer to Materials and methods Section for the method of breakpoint- and breakend-level assessment for translocations). It is worth noting that SVIM did not assign genotypes for all the translocations and most of the duplications, which resulted in lower statistics. Overall, the results suggest that cuteSV is a good versatile SV caller, i.e., it is able to sensitively detect various types of SVs without lack of accuracy, and their genotypes can be correctly recognized as well." (line 24-54, page 4 of Results section)*

*"6167, 9899 and 44 deletions, insertions and inversions were extracted from CHM1 sample callsets (nstd137 in dbVAR database). The extracted SVs are non-overlapping and meet the condition that experiment IDs are 1 or 2 and all of ACs, ANs and AFs are higher than 0. Similarly, 3712 and 380 non-overlapping duplications and translocations were extracted from KWS1 sample callsets (nstd106 in dbVAR database), respectively. Then, the five types of SVs were respectively integrated into human reference genome (hs37d5) to build five in silico donor genomes to generate simulated datasets with VISOR simulator. For a specific type of SVs, the simulation is done by the following two steps.*

*(1) The SVs were transformed into BED format according to the requirement of VISOR HACk. Deletions, insertions or inversions were directly spiked into the reference to generate haplotype in silico donor genomes correspondingly. Employed translocations and duplications were preprocessed as reciprocal translocations and tandem duplications with CN=2 before input to VISOR, respectively. It is also worth noting that, translocations in same strand orientations (e.g. forward-forward and reverse-reverse reciprocal translocations) have 2 kinds of breakpoint combinations and 4 kinds of breakend combinations, and translocations in different strand orientations (e.g. forward-reverse and reverse-forward reciprocal translocations) have 4 kinds of breakpoint combinations and 4 kinds of breakend combinations. A schematic illustration is in Supplementary Figure 11.*

*(2) For a given donor genome, its in silico chromosomes were randomly selected as “homozygous” and “heterozygous” chromosomes to mimic homozygous and heterozygous SVs. For a “homozygous” chromosome, all the reads were simulated from the in silico chromosome with SVs. For a “heterozygous” chromosome, 50% of the reads were simulated from the in silico chromosome and 50% from the corresponding reference chromosome. With this rule, four PacBio-like datasets in various coverages (5×, 10×, 20× and 30×) were produced by VISOR LASeR (using PBSIM and minimap2 with default setting). Thus, there are 20 datasets produced for the five donor genomes and the specific sets of SVs and their genotypes were used as ground truth.*

*The used command lines for data simulation are in Supplementary Notes." (line 9-44, page 14 of Materials and methods section)*

- Following the previous point, the authors should still report on the real datasets precision and recall. One of the more important questions is what is the basis for ground through for NA19240, they have used the validation data from a previous study but for HG002, it is not clear how the ground truth has been obtained.

*Response:*

*Following your suggestions, we used the ground truth of NA19240, which is from the previous study by Chaisson et al. (PMID: 30992455). We have cited this study in the manuscript.*

*We have mentioned in the current version of the manuscript:*

*“We collected a callset from a previous study [50] for this sample and considered it as the ground truth. It contains a total number of 37657 SVs (size > 50 bp), including 17950 deletions, 19482 insertions/duplications and 225 inversions.” (line 53-58, page 7 of Results section)*

*“50. Chaisson MJP, Sanders AD, Zhao X, Malhotra A, Porubsky D, Rausch T, Gardner EJ, Rodriguez OL, Guo L, Collins RL, et al: Multi-platform discovery of haplotype-resolved structural variation in human genomes. Nat Commun 2019, 10:1784.” (line 8-11, page 20 of References section)*

- Inversions are one of the more challenging SV types to discover, it is not clear why they are removed from the evaluation of HG002.

*Response:*

*Thanks for pointing this out. There are still only deletions and insertions in the current version of the SV callset of HG002 release by GIAB (https://doi.org/10.1101/664623). The authors of that article claimed a new version benchmark set consisting of more SV calls in complex genomic regions and of more challenging types. However, to our knowledge that this callset has not been published yet. Therefore, we employed the current version of GIAB HG002 callset as ground truth. We agree with you that the used callset has some limitations, for example, some important SV types such as inversions cannot be assessed.*

*To more comprehensively benchmark various types of SVs, we used GIAB Ashkenazi Trio PacBio CLR datasets (HG002, HG003 and HG004) to assess the recall rates and Mendelian-Discordance-Rates (MDRs). This assessment includes all types of SVs, i.e., insertions, deletions, inversions, duplications and translocations.*

*We have mentioned in the current version of the manuscript:*

*"To assess the ability to the detection of various types of SVs (i.e., insertions, deletions, inversions, duplications and translocations) more comprehensively, we further employed GIAB Ashkenazi Trio PacBio CLR datasets (HG002, HG003 and HG004) to assess the recall rates and Mendelian-Discordance-Rates (MDRs). cuteSV and SVIM obtained >95% mean recall rate, i.e., more than 95% homozygous parental SVs has been confirmed in the offspring (Figure 3C and Supplementary Table 9). cuteSV was 1% lower than SVIM on recall rate, however, we realized that this does not mean a lower sensitivity of cuteSV, but due to that about 15% SVs in parental callsets discovered by SVIM had no genotypes so that they cannot be assessed and decreased the total number of homozygous parental SVs. Meanwhile, the MDR of cuteSV is lowest (7.36%, Figure 3D and Supplementary Table 9), indicating that its callsets were more plausible. The MDRs of SVIM (7.62%) and PBSV (9.25%) are also comparable, and much lower than that of Sniffles (17.45%)." (line 41-55, page 5 of Results section)*

- Paper should explain what types of calls cuteSV can capture while other methods fail. It is not clear if the precision/recall improvement is due to method or the events not being targeted by the competitor tools.

*Response:*

*cuteSV is a versatile SV caller and the higher precision/recall is not because of that it detects some specific types of SVs which other state-of-the-art callers not being targeted. Mainly, the yields of cuteSV come from its tailored heuristic approaches to better handle some difficult SVs caused by fragile read alignments and heterozygosity. Those SVs are still hard to detect by state-of-the-art approaches. We explained this in Discussion section.*

*Following your suggestions, we have revised the manuscript:*

*"Mainly, the proposed approach has four advantages as follows.*

*1) The heuristic SV signature extraction methods enable to well-handle the fragile read alignments around SV breakpoints, which helps to build high quality SV evidence. This is one of the major reasons that cuteSV outperforms state-of-the-art tools. An example is in Supplementary Figure 6. It shows how a 272bp insertion event was successfully discovered by cuteSV, while other benchmarked SV callers were affected by the multiple insertions and deletions in read alignments.*

*2) The clustering-and-refinement method enables to distinguish the reads from the multiple SV alleles in the same loci and generate correct signature clusters. Thus, complex heterozygous SVs can be detected and genotyped sensitively and accurately, which also outperforms state-of-the-art tools. Two examples are shown in Supplementary Figure 7. One is a heterozygous insertion event (a 108bp and a 36bp insertion in the same region) and the other is a heterozygous deletion event (a 123bp and a 37 bp deletion in the same region). Only cuteSV and PBSV successfully discovered them." (line 19-36, page 8 of Results section)*

- Paper should emphasize the novel ideas employed in cuteSV that lead to improved accuracy/recall.

*Response:*

*Following your suggestions, we have revised “Overview of cuteSV” in the Results section to describe the motivations and main ideas of cuteSV more clearly.*

*We have mentioned in the current version of the manuscript:*

*"Using sorted BAM file(s) as input, cuteSV extracts large insertions/deletions and split alignments in aligned reads as SV signatures, and clusters and analyzes them to call SVs. The approach has three major steps as follows.*

*Step 1: cuteSV uses multiple signature extraction methods to comprehensively collect the signatures of various types of SVs. Furthermore, the insertions and deletions are heuristically combined to recover the evidence of real SVs from fragile alignments.*

*Step 2: cuteSV uses a specifically designed clustering-and-refinement approach to cluster the chimerically aligned reads in local regions, and further refines the clusters to precisely distinguish the SV signatures from heterozygous SVs.*

*Step 3: cuteSV uses several tailored rules to implement SV calling and genotyping based on the refined clusters of SV signatures.*

*A schematic illustration is in Figure 1 and more details are in Materials and methods Section.*

*The major contribution of cuteSV approach is that it uses tailored heuristics to address three difficult technical issues in the alignment-based SV calling.*

*Firstly, due to the scoring systems of read aligners, some large SV events could be divided into several smaller insertions/deletions in a local region. Such cases are usually treated as multiple smaller SVs mistakenly. However, in step 1 of cuteSV, a heuristic method is used to combine insertions/deletions in nearby genomic regions to unbroken signatures of larger SVs. This method not only reduces the errors caused by the fragile read alignments, but also enables to produce more homogenous SV signatures from various reads, which is beneficial to the processing of later steps.*

*Secondly, reads spanning the same SV usually have heterogeneous breakpoints in their alignments, which also cause false positive SV calls. In Step2 of cuteSV, the specifically designed clustering-and-refinement approach enables to adaptively cluster alignment breakpoints mapped to relatively large local regions but potentially belonging to identical SVs, so that heterogeneous breakpoints can be merged more effectively and more false positives can be prevented.*

*Thirdly, there are occasionally complex heterozygous SVs which multiple SV alleles are in same loci. Such SVs are difficult to detect and genotype. In Step2 of cuteSV, they are handled by a novel heuristic refinement on the SV signature clusters. That is, cuteSV investigates each of the clusters to check if there are heterogeneous signatures. If so, it implements a precise analysis to distinguish the reads potentially from various SV alleles and re-cluster them into more homogeneous sub-clusters. Taking the advantage of this approach, the complex SVs can be detected and genotyped correctly.*

*In addition, cuteSV uses a block division-based approach to process input data in a parallel way with multiple CPU threads. This implementation greatly helps it to achieve outstanding scaling performance, which has been demonstrated on several datasets in various sizes (see below for details)." (line 27-58, page 3 and line 1-20, page 4 of Results section)*

- While low memory requirements of cuteSV is impressive, most analysis will be bottle-necked by the read alignment step. If trade off between memory usage and speed is possible, having an option to move disk usage to memory would improve the tool for users who have available memory.

*Response:*

*cuteSV divides the input BAM file into blocks in same size by genomic coordinates, and the used multiple CPU threads separately process the blocks in a parallel way. In details, the aligned reads in a certain block are scanned from upstream to downstream, and cuteSV subsequently analyzes each of the reads to extract and cluster the SV signatures. During the processing, the main consumption of memory is the data structure to load detailed read alignments. However, the size of memory use is not closely related to the processing speed, unless the used memory is too small which may cause frequent I/O operations. Otherwise, the speed improvement with increasing memory use is marginal. Under such circumstance, cuteSV uses a tuned configuration which fully considers both of the speed and the memory footprint. However, considering the relatively slow speed of cuteSV in single thread, we also plan to develop faster SV signature extraction methods. Thank you very much for your suggestion, that the tradeoff between memory and speed could be a very useful starting point to re-think about the acceleration of cuteSV, especially the development of novel data structures.*

- In the supplementary figure 4, the difference between cuteSV and other methods seems to be marginal.

*Response:*

*To manifest the differences among the benchmarked SV callers more clearly and intuitively, we re-plotted this figure by histogram instead of bar chart, and adjusted the axis from 0~100% to 0~70% so as to enlarge the discrepancy in form. Please refer to Supplementary Figure 5 in the current version of the manuscript. Moreover, the detailed statistics are given in Supplementary Table 16.*

- In equation 13 2nd condition seems to have a typo.

*Response:*

*Thank you very much for pointing this out, we have corrected it as follows:*

*"*

*max(comp_s - 1kbp, base_s) ≤ min(comp_e + 1kbp, base_e)*

*min(comp_L, base_L) ⁄ max(comp_L, base_L) ≥ 0.7 (13)*

*comp_t = base_t*

*where comp_s, comp_e, comp_L and comp_t indicate start coordinate, stop coordinate, size and SV class of a prediction. And base_s, base_e, base_L and base_t are starting coordinate, end coordinate, size and SV class of a call in the truth set, respectively." (line 22-30, page 15 of Materials and methods section)*

**Second round of review**

**Reviewer 1**

Thanks for the revised manuscript. The authors followed my suggestions and added a simulation benchmark, genotype likelihoods, quality scores and a review of assembly-based approaches. The manuscript text and figures also improved considerably. The new runtime & memory benchmark suggests that the method is well-engineered and usable in practical applications. The added simulation benchmark revealed that the performance of SV site discovery is very similar among cuteSV, Sniffles, PBSV and SVIM but genotyping accuracy appears to be best for cuteSV. I was surprised to see a quite noticeable drop in F1 scores for all methods if genotypes are evaluated even at high depth (30x). That is likely related to an under-/overcalling of heterozygous variants due to the high error-rate. For final publication it would be informative to add genotype confusion matrices to the Supplement that show all ground-truth genotypes (homozygous reference, heterozygous, homozygous alternative) in relation to the called genotypes.

**Reviewer 2**

Manuscript is significantly improved since last revision. All the revision requests are added to the paper.

- A Venn diagram for overlaps of the calls from different tools, like the one provided for cuteSV calls on different sequencing technologies, may be interesting.

- Also making the distinction of accuracy between cigar only calls and split alignment calls would improve the manuscript.

**Authors Response**

**Point-by-point responses to the reviewers’ comments:**

Reviewer: 1

- Thanks for the revised manuscript. The authors followed my suggestions and added a simulation benchmark, genotype likelihoods, quality scores and a review of assembly-based approaches. The manuscript text and figures also improved considerably. The new runtime & memory benchmark suggests that the method is well-engineered and usable in practical applications. The added simulation benchmark revealed that the performance of SV site discovery is very similar among cuteSV, Sniffles, PBSV and SVIM but genotyping accuracy appears to be best for cuteSV. I was surprised to see a quite noticeable drop in F1 scores for all methods if genotypes are evaluated even at high depth (30x). That is likely related to an under-/overcalling of heterozygous variants due to the high error-rate. For final publication it would be informative to add genotype confusion matrices to the Supplement that show all ground-truth genotypes (homozygous reference, heterozygous, homozygous alternative) in relation to the called genotypes.

*Response:*

*Thanks for the suggestion. We have added genotype confusion matrices on the 30X simulated datasets for various SV callers in supplementary material (Additional file 1: Table S7). It is observed that there do be under- and over-calling of heterozygous SVs by various SV calling approaches.*

*We have mentioned this in the current version of the manuscript:*

*“Moreover, we assessed the accuracy of genotyping on the 30× simulated datasets with genotype confusion matrices (Additional file 1: Table S7). It is observed that cuteSV, PBSV and SVIM recognized heterozygous and homozygous variants more accurately. Sniffles often misclassified homozygous variants as heterozygous especially for deletions, insertions and duplications.” (line 53-57, page 4 of Results section)*

---------------------------------------------------

Reviewer: 2

- A Venn diagram for overlaps of the calls from different tools, like the one provided for cuteSV calls on different sequencing technologies, may be interesting.

*Response:*

*Thanks for the suggestion. We have added Venn diagrams of the callsets produced by various tools on PacBio CLR, CCS and ONT PromethION data, respectively (Fig. 4C, 4D and 4F), and the intersection statistics are in Additional file 1: Table S14. It is observed that, most of the calls are supported by two or more SV callers. However, there are still a small proportion of tool-unique calls. Moreover, on PacBio data, cuteSV had fewest unique calls. On ONT data, the number of unique calls produced by cuteSV is slightly higher, due to that it detected more deletions.*

*We have mentioned in the current version of the manuscript:*

*“Moreover, Venn diagrams of the SV callsets produced by various approaches on the HG002 PacBio CLR, PacBio CCS and ONT PromethION datasets were plotted (Fig. 4C, 4D, 4E and Additional file 1: Table S14). For PacBio CLR and CCS data, there are 22804 and 29733 > 30 bp SV calls made by all the approaches, respectively, and cuteSV has fewer unique calls (3818 for CLR and 2517 for CCS data) than other tools. For ONT PromethION data, only the callsets of cuteSV, Sniffles and SVIM were considered, and 35592 > 30 bp SV calls were made by all of the three tools, the trend is similar to that of PacBio data. However, the number of unique calls by cuteSV (i.e., 6029 of 51563) is slightly higher than that of the other two methods. This is mainly due to that cuteSV detected more deletions.” (line 52-59, page 6 and line 1-2, page 7 of Results section)*

- Also making the distinction of accuracy between cigar only calls and split alignment calls would improve the manuscript.

*Response:*

*We have assessed the callset of cuteSV on the 30x simulated datasets, to investigate the accurate calls made by CIGAR signatures and split-alignment signatures. The statistics are in Additional file 1: Table S8. Mainly, it is observed that most of deletions and insertions are detected with only CIGARs, and only a small proportion of the calls are produced with split-alignment signatures. This is partially due to that the employed aligner has a strong ability to handle deletions and insertions in reads to make informative CIGARs. It is also worthnoting that inversions, duplications and translocations can only be detected based on split-alignment signatures.*

*We have mentioned in the current version of the manuscript:*

*“We also investigated the distinction of accurate calls of cuteSV made by various kinds of signatures (on the 30x simulated datasets, Additional file 1: Table S8). It shows that most of accurate insertion (93.59%) and deletion (96.18%) calls are produced with only CIGAR signatures, and the proportions of accurate calls produced with only split-alignment signatures and mixed signatures (CIGAR and split alignment) are low. This indicates that the employed aligner has a strong ability to handle deletions and insertions in reads to make informative CIGARs. It is also worth noting that this assessment was only done for insertions and deletions, since other types of SVs (inversions, duplications and translocations) can only be detected based on split-alignment signatures.” (line 1-10, page 5 of Results section)*
